# Einkorn genomics sheds light on history of the oldest domesticated wheat

**DOI:** 10.1038/s41586-023-06389-7

**Published:** 2023-08-02

**Authors:** Hanin Ibrahim Ahmed, Matthias Heuberger, Adam Schoen, Dal-Hoe Koo, Jesus Quiroz-Chavez, Laxman Adhikari, John Raupp, Stéphane Cauet, Nathalie Rodde, Charlotte Cravero, Caroline Callot, Gerard R. Lazo, Nagarajan Kathiresan, Parva K. Sharma, Ian Moot, Inderjit Singh Yadav, Lovepreet Singh, Gautam Saripalli, Nidhi Rawat, Raju Datla, Naveenkumar Athiyannan, Ricardo H. Ramirez-Gonzalez, Cristobal Uauy, Thomas Wicker, Vijay K. Tiwari, Michael Abrouk, Jesse Poland, Simon G. Krattinger

**Affiliations:** 1 Plant Science Program, Biological and Environmental Science and Engineering Division, King Abdullah University of Science and Technology (KAUST), Thuwal, Saudi Arabia; 2 Center for Desert Agriculture, King Abdullah University of Science and Technology (KAUST), Thuwal, Saudi Arabia; 3 Department of Plant and Microbial Biology, University of Zurich, Zurich, Switzerland; 4 Department of Plant Science and Landscape Architecture, University of Maryland, College Park, MD USA; 5 Wheat Genetics Resource Center and Department of Plant Pathology, Kansas State University, Manhattan, KS USA; 6 John Innes Centre, Norwich Research Park, Norwich, UK; 7 INRAE, CNRGV French Plant Genomic Resource Center, Castanet-Tolosan, France; 8 Crop Improvement and Genetics Research Unit, Western Regional Research Center, Agricultural Research Service, United States Department of Agriculture, Albany, CA USA; 9 KAUST Supercomputing Core Lab (KSL), King Abdullah University of Science and Technology (KAUST), Thuwal, Saudi Arabia; 10 Global Institute for Food Security, University of Saskatchewan, Saskatoon, Saskatchewan Canada

**Keywords:** Plant genetics, Agricultural genetics, Evolutionary genetics, Genome evolution, Centromeres

## Abstract

Einkorn (*Triticum monococcum*) was the first domesticated wheat species, and was central to the birth of agriculture and the Neolithic Revolution in the Fertile Crescent around 10,000 years ago^1,2^. Here we generate and analyse 5.2-Gb genome assemblies for wild and domesticated einkorn, including completely assembled centromeres. Einkorn centromeres are highly dynamic, showing evidence of ancient and recent centromere shifts caused by structural rearrangements. Whole-genome sequencing analysis of a diversity panel uncovered the population structure and evolutionary history of einkorn, revealing complex patterns of hybridizations and introgressions after the dispersal of domesticated einkorn from the Fertile Crescent. We also show that around 1% of the modern bread wheat (*Triticum aestivum*) A subgenome originates from einkorn. These resources and findings highlight the history of einkorn evolution and provide a basis to accelerate the genomics-assisted improvement of einkorn and bread wheat.

## Abstract (web summary)

Around 1% of the A subgenome of modern bread wheat (*Triticum aestivum*) originates from einkorn (*Triticum monococcum*), the first domesticated wheat species.

## Main

Einkorn (*T. monococcum*) was the first wheat species that humans domesticated around 10,000 years ago in the Fertile Crescent, a region in the Near East that is often referred to as the Cradle of Civilization^1,2^. Wild einkorn was an ingredient of the oldest known bread-like products, baked by hunter-gatherers in modern-day Jordan four millennia before the dawn of agriculture^3^. Einkorn had a pivotal role in the establishment of agriculture in the Fertile Crescent and it is the only diploid wheat species (2*n* = 2*x* = 14, A^m^A^m^ genome) of which both wild and domesticated forms exist. A noticeable morphological difference between wild and domesticated einkorn is the grain dispersal system. Wild einkorn has a fragile rachis that facilitates seed dispersal, whereas the rachis in domesticated einkorn is non-brittle^4^. Einkorn is closely related to *Triticum urartu*, the A genome donor of tetraploid durum (*Triticum durum*) and hexaploid bread wheats (*T. aestivum*)^5^. In contrast to *T. urartu*, wild and domesticated einkorn have a long history of cultivation and human selection in diverse environmental conditions, which makes einkorn a valuable source of genetic variation for wheat breeding. Multiple natural and artificial einkorn introgressions into bread wheat containing agriculturally important genes have been described^6–10^. Population genetic analyses indicate that wild einkorn clusters into three distinct groups (races α, β and γ) and point to a region around the Karacadağ mountains in Southeastern Turkey as the site of einkorn domestication^11–17^.

Here we establish and analyse a comprehensive set of genomic resources for einkorn, including de novo annotated chromosome-scale reference assemblies of one wild and one domesticated einkorn accession, as well as whole-genome sequencing of an einkorn diversity panel. Our results unravel the complex evolutionary history of einkorn and offer insights into the genome dynamics of Triticeae, including the centromere structure, while establishing valuable resources that augment the genomic toolbox for wheat improvement.

## Chromosome-scale einkorn assemblies

**Fig. 1:**
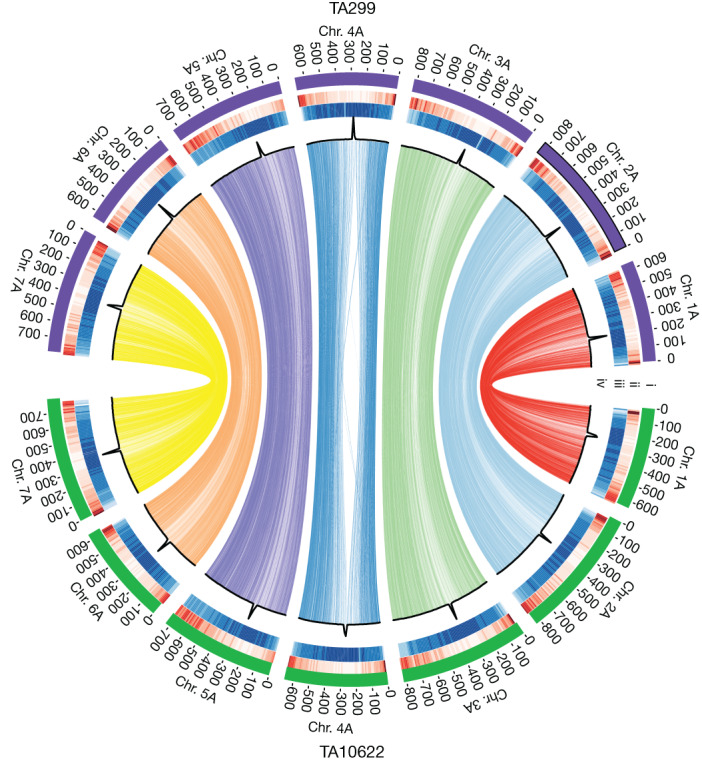
Einkorn genome structure and functional features. Circos plot showing synteny between the assemblies of wild einkorn (TA299) and domesticated einkorn (TA10622). The tracks depict structural and functional features of the two einkorn reference assemblies. The number and length of pseudomolecules (i), gene density along pseudomolecules (ii), repeat density along pseudomolecules (iii) and CENH3 ChIP–seq read coverage along pseudomolecules (iv) are shown. Peaks in each pseudomolecule define the centromeres (iv). The lines in the inner circle represent 17,586 orthologous high-confidence genes between TA299 and TA10622. Only relationships between the same chromosomes are shown.

We generated reference assemblies of two einkorn accessions using a combination of PacBio circular consensus sequencing^18^, optical mapping^19^ and chromosome conformation capture^20^ (Extended Data Table 1, Supplementary Table 1 and Supplementary Fig. 1). TA10622 is a domesticated einkorn landrace (*T. monococcum* L. subsp. *monococcum*) with non-brittle rachis that was collected in Albania at the beginning of the twentieth century. Wild einkorn accession TA299 (*T. monococcum* L. subsp. *aegilopoides*; race α) was collected during an expedition in 1972 in northern Iraq^21^ and has a brittle rachis. Assembly integrities were verified using an einkorn genetic map (Supplementary Tables 2 and 3). We observed a high degree of collinearity across the two sets of pseudomolecules (Fig. 1 and Supplementary Fig. 2) and between the two einkorn assemblies and the bread wheat A subgenome (Supplementary Fig. 3). The most obvious exceptions were the well-described rearrangements of bread wheat chromosome 4A, which experienced inversions and translocations in polyploid wheat^22^. We annotated 32,230 and 32,090 high-confidence gene models on the 7 pseudomolecules of TA299 and TA10622, respectively (BUSCO scores of 99.2% for TA299 and 99.4% for TA10622) (Supplementary Tables 4 and 5).

**Extended Data Fig. 1:**
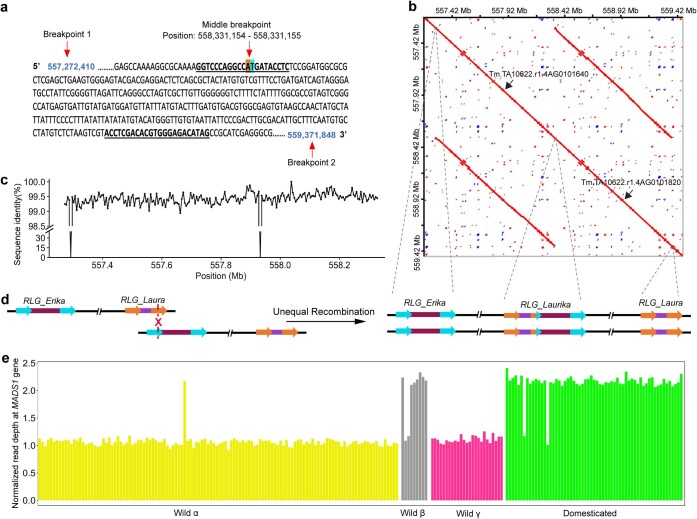
Characterization of a large tandem duplication in einkorn. **a**, Sequence around the middle breakpoint of the two tandemly duplicated segments on chromosome 4A of TA10622. The 1 Mb duplication was confirmed by designing a PCR marker across the breakpoint. Primer sequences are underlined and indicated in bold. The nucleotides at the breakpoint are highlighted in orange (located in the *RLG_Laura* element) and in blue (located in the *RLG_Erika* element). **b**, Dot plot showing a comparison of a 2.3 Mb region of chromosome 4A of TA10622 against itself. The two red lines indicate the megabase-sized tandem duplication. The positions of the MADS-box transcription factor genes are indicated by black arrows. A schematic representation of the retroelements is shown at the bottom. Arrows indicate long terminal repeats (LTRs). **c**, Sequence identity across the two duplicated segments, calculated in 5 kb non-overlapping windows. **d**, Schematic diagram showing the proposed unequal recombination between two retrotransposons that led to the tandem duplication. **e**, The presence - absence of the tandem duplication on chromosome 4A was estimated by normalized read coverage across the *MADS1* gene (including 2 kb of flanking sequence). Outliers: α: TA316, β: TA10573 and TA10910, domesticated einkorn: TA10548 and TA10577.

Previous short-read-based wheat assemblies often did not resolve large tandem and segmental duplications^23,24^. On chromosome 4A of TA10622, we identified an approximately 1 Mb tandem duplication (Extended Data Fig. 1a,b). The two segments were 1,058,744 bp and 1,040,693 bp in length, with 98.3% sequence identity on average (Extended Data Fig. 1b,c). The most plausible explanation for the origin of this large tandem duplication is an unequal recombination between retrotransposons^25^ (Extended Data Fig. 1d). Each of the duplicated segments contained one high-confidence gene encoding a MADS-box transcription factor (*Tm.TA10622.r1.4AG0101640* and *Tm.TA10622.r1.4AG0101820*) (Extended Data Fig. 1b), which have important roles in the regulation of plant growth and development^26–29^. The corresponding segment in the wild TA299 accession was not duplicated, carrying instead a single copy of the MADS-box gene. We estimated the presence/absence of the tandem duplication across a diversity panel comprising 218 wild and cultivated einkorn accessions (Extended Data Fig. 1e). All but one wild einkorn accession belonging to races α and γ had one copy of the corresponding segment, whereas most domesticated einkorn accessions carried the tandem duplication. Wild einkorn accessions belonging to race β, the proposed progenitor of domesticated einkorn, had two copies for this segment, indicating that the tandem duplication predated domestication (Extended Data Fig. 1e). The tandem duplication was specific to einkorn and was not found in bread wheat.

**Extended Data Fig. 2:**
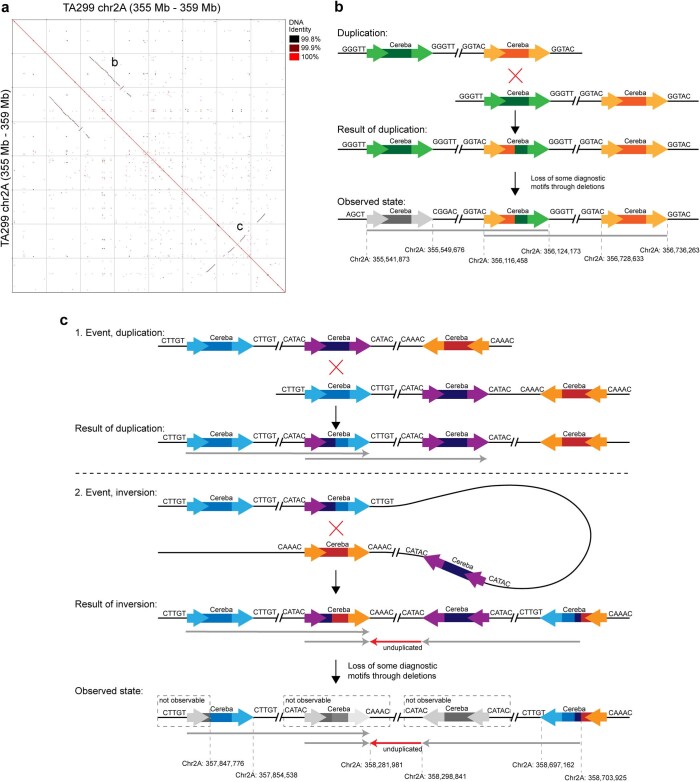
Evolutionary origin of large-scale duplications and inversions in the centromere of TA299 chromosome 2A. **a**, Dot plot alignment of a segment inside the centromere of chromosome 2A. A large duplication is labelled with “b” and a duplication/inversion with “c”. **b**, Evolutionary model for the duplication event b. We propose that the large duplication originated from unequal recombination between two *RLG_Cereba* retrotransposons that were ~700 kb apart, resulting in the duplication of the entire sequence between them. The *RLG_Cereba* elements that served as templates for the unequal recombination are shown in red and green. The 5 bp target site duplications (TSD) produced by their insertions were used as diagnostic sequences to identify the recombinant element in the centre of the two duplicated units. Parts of the duplication that were deleted in later events are shown in grey (this includes one of the *RLG_Cereba* copies that served as a template for the initial event). **c**, The second duplication/inversion “c” occurred following the same mechanisms. The duplication was followed by a second independent event resulting in an inversion that affected nearly the same region. The inversion was caused by recombination between different *RLG_Cereba* elements near the original duplication breakpoints but in the opposite orientation. Subsequently, deletions near the borders of the inverted segment removed some of the diagnostic motifs (indicated in grey). We emphasize that the presented model is based exclusively on homology-based recombination between different *RLG_Cereba* retrotransposons and that it is possible that events resulting in a duplication plus an inversion may also involve alternative mechanisms.

In addition to the megabase-sized tandem duplication on chromosome 4A, we successfully resolved large duplications in the highly repetitive centromere regions. The centromere of chromosome 2A in TA299 contained two large duplications of around 700 kb each, one of which was followed by an inversion. Both events can be traced to unequal recombination between *RLG_Cereba* retrotransposons (Extended Data Fig. 2). These results highlight the superiority of long-read-based genome assemblies to resolve and study the dynamics of large tandem duplications.

## Analysis of complete einkorn centromeres

Centromeres are critical regions of eukaryotic chromosomes for the assembly of the spindle apparatus and cell division^30^. Although centromere function is conserved across eukaryotes, there is considerable variation in the underlying genomic sequences. Centromeres often remain as persistent gaps in genome assemblies owing to their complex, highly repetitive sequences. Many centromeres are colonized by centromeric satellite repeats of 130–180 bp in size (corresponding to the length of DNA wrapped around a nucleosome) and/or centromere-specific transposable elements (TEs). Centromere identity is defined epigenetically by the presence of centromere-specific CENH3 histone variants (CENP-A in mammals)^31^.

**Fig. 2:**
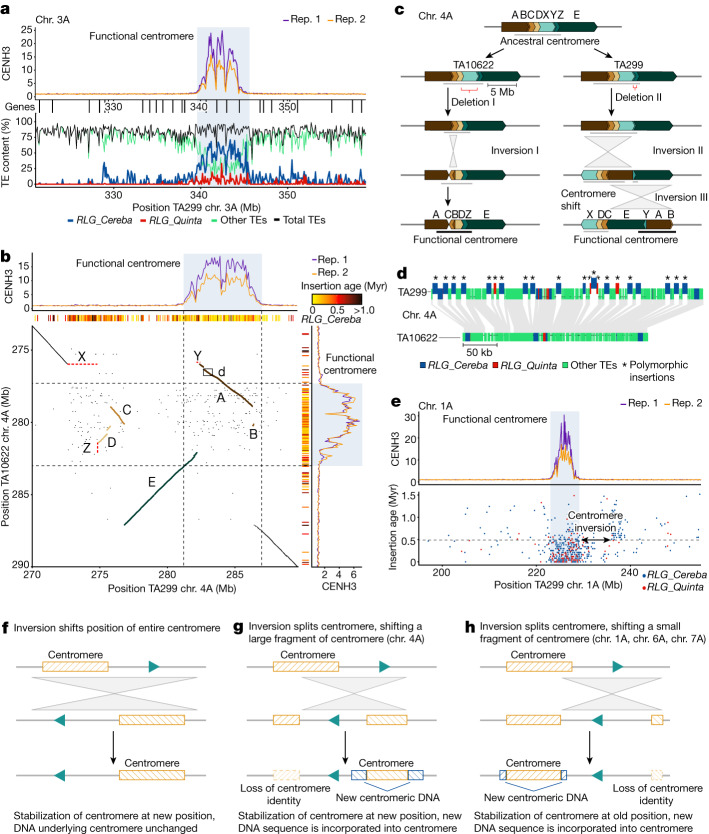
Dynamics of einkorn centromeres. **a**, The composition of the TA299 chromosome 3A centromere. The top track shows CENH3 ChIP–seq coverage. The vertical lines underneath the track indicate genes. The bottom track shows TE composition. The *x* axis indicates chromosomal positions in megabases. The functional centromere is highlighted (blue shading). **b**, Dot plot alignment of chromosome 4A centromeric regions of TA299 (horizontal) and TA10622 (vertical). CENH3 ChIP–seq coverage and positions of *RLG_Cereba* insertions are aligned with the dot plot. *RLG_Cereba* insertion age is colour-coded in million of years (Myr). Rearranged chromosomal segments are shown in colours that correspond to those in **c**. The small rectangle indicates an approximately 400 kb region that is shown in detail in **d**. **c**, Evolutionary model explaining the organization of chromosome 4A centromeres in TA10622 and TA299. A–E indicate segments that experienced inversions compared with the ancestral centromere. X–Z represent segments that were deleted in one of the two accessions. **d**, Comparison of the shifted TA299 chromosome 4A centromere with its counterpart in TA10622. Conserved sequences are connected by the shaded grey areas. New TE insertions are shown partially raised. All new TE insertions are of the *RLG_Cereba* and *RLG_Quinta* families. **e**, Evidence of an additional inversion of around 10 Mb in chromosome 1A that moved part of the functional centromere (indicated by the two-headed arrow). Top, CENH3 ChIP–seq coverage. Bottom, the chromosomal positions of *RLG_Cereba* and *RLG_Quinta* retrotransposons (*x* axis) and their insertion age (*y* axis). The distribution and insertion ages of retrotransposons indicate that the inversion occurred around 500,000 years ago (grey dashed line) in a common ancestor of TA10622 and TA299. **f**,**g**, Examples of how inversions can cause centromere shifts. **h**, Example of how a centromere remains at or near to its original location after a segment is moved by an inversion.

**Extended Data Fig. 3:**
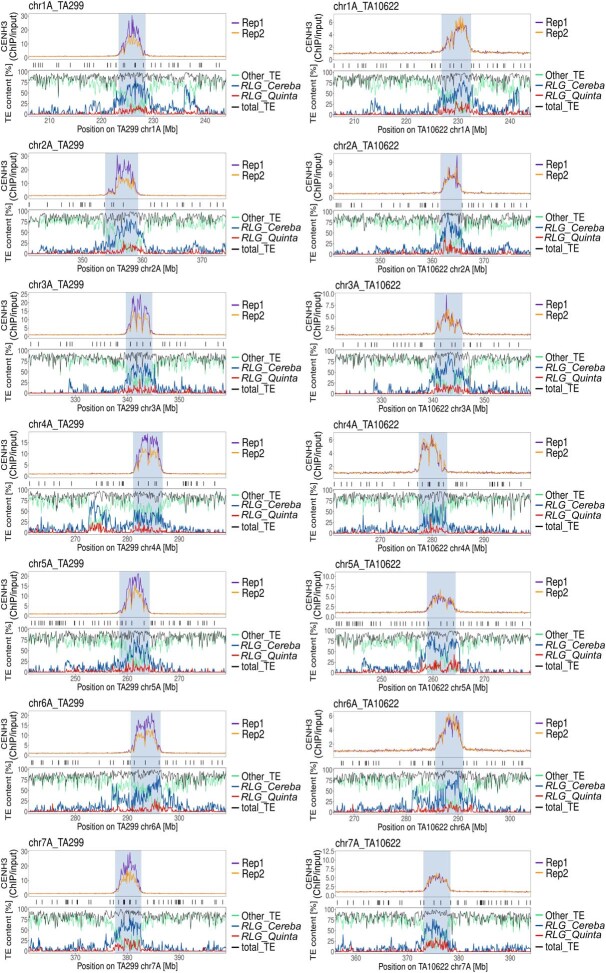
Definition of functional centromeres (shaded in blue) in *T. monococcum* accessions TA299 (left column) and TA10622 (right column) from CENH3 ChIP-Seq data. Centromeric regions plus ~15 Mb of flanking regions are shown. The top graph of each panel shows the ratio of CENH3 ChIP-Seq reads divided by input control reads. ChIP-Seq and control reactions were performed in duplicates, which are shown in purple (Rep1) and orange (Rep2). The region of functional centromeres was determined based on epic2 ChIP-Seq peak call density and is indicated by a shaded area. The middle track below shows the positions of genes as vertical lines. The bottom graph shows the average transposable element (TE) content in 100 kb windows. Note that *RLG_Cereba* and *RLG_Quinta* retrotransposons are highly enriched in functional centromeres, while other TE families dominate outside of centromeres.

We performed chromatin immunoprecipitation with sequencing (ChIP–seq) analysis of CENH3, which identified one distinct region per chromosome in TA10622 and TA299 with high read coverage, indicating the positions of functional centromeres (Fig. 2a, Extended Data Fig. 3, Supplementary Fig. 4 and Supplementary Tables 6 and 7). In addition to the high CENH3 coverage, we found that einkorn centromeres are local minima of CpG methylation and H3K4me3 histone modification (Supplementary Figs. 5 and 6). Crucial for our analysis was that centromeric regions in both accessions were assembled contiguously without sequence gaps (Supplementary Fig. 7) and validated by optical map data. The only exception was the chromosome 2A centromere of TA10622, which carried two small gaps. In contrast to previous studies that investigated small portions of individual wheat centromeres^32,33^ or highly fragmented assemblies^34^, the assembly of complete einkorn centromeres enabled us to perform a detailed analysis of the structure and dynamics across whole Triticeae centromeres. Functional einkorn centromeres ranged from 4 to 5.8 Mb in size, with an average of 5.46 Mb (TA299) and 5.24 Mb (TA10622). In both einkorn accessions, the functional centromeres contained only one to nine genes, except for chromosome 7A of accession TA299, which contained 39 annotated genes. Most genes residing in CENH3-enriched domains were not expressed, whereas genes located in functional centromeres, but outside of CENH3-enriched domains, showed varying expression levels (Supplementary Fig. 8).

**Extended Data Fig. 4:**
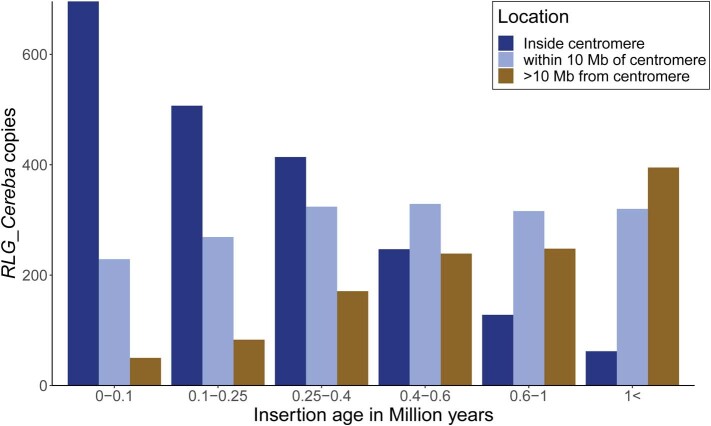
Comparison of *RLG_Cereba* insertion ages with physical distance from centromeres. Note that ~95% of *RLG_Cereba* elements younger than 1 million years are found inside functional centromeres.

**Extended Data Fig. 5:**
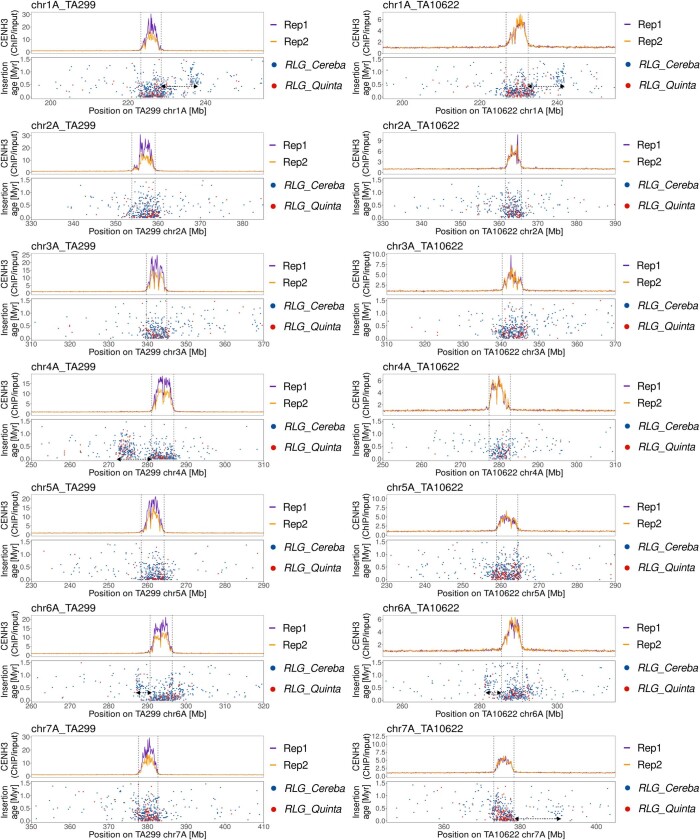
CENH3 ChIP-Seq read coverage in relation to insertion ages of centromere-specific retrotransposons in *T. monococcum* accessions TA299 (left column) and TA10622 (right column). The top panels show the ratio of CENH3 ChIP-Seq reads divided by input control reads. ChIP-Seq and control reactions were performed in duplicates, which are shown in purple (Rep1) and orange (Rep2). The bottom panels show the chromosomal positions of *RLG_Cereba* (blue) and *RLG_Quinta* (red) retrotransposons (x-axis) and their insertion age (y-axis). The youngest retrotransposon insertions are generally found in the functional centromeres. Retrotransposon insertions on chromosomes 1A, 4A, 6A and 7A indicate that parts of the functional centromeres were moved at different evolutionary time points. The observed patterns can be explained by large-scale inversions that moved parts of centromeres (indicated by two-headed arrows, with y positions indicating the approximate time of the inversion event).

In *Arabidopsis thaliana*, centromeres contain megabase-scale arrays of approximately 178 bp tandem satellite repeats^35^. Similar centromeric satellite repeat arrays have been reported in different grass species^36–38^. By contrast, we did not detect high-copy, centromere-specific satellite repeats in einkorn (Supplementary Fig. 9 and Supplementary Note 1). The majority of the functional einkorn centromeres comprised TEs (91.25–97.35%). Although the TE proportions in functional einkorn centromeres were comparable to the chromosome arms, the exact TE composition differed markedly. The centromere-associated retrotransposon family *RLG_Cereba* was predominant in the einkorn functional centromeres (around 70%), followed by *RLG_Quinta* elements (around 20%). Outside the functional centromeres, these two retrotransposon families were rare and other TE families dominated^39^ (Fig. 2a and Extended Data Fig. 3). We identified approximately 2,500 full-length *RLG_Cereba* and around 1,000 full-length *RLG_Quinta* retrotransposons in each of the two einkorn assemblies. The youngest *RLG_Cereba* and *RLG_Quinta* insertions mapped inside the currently functional centromeres, supporting a model that the integrase enzyme encoded by *RLG_Cereba* elements recognize CENH3-containing nucleosomes^40^. The evolutionary youngest *RLG_Cereba* subpopulation was almost exclusively found in functional centromeres (Extended Data Fig. 4 and Supplementary Fig. 10). Older elements were ‘pushed away’ from functional centromeres by the more recent insertions (Extended Data Fig. 4). These results indicate that *RLG_Cereba* and *RLG_Quinta* retrotransposons are suitable markers for the location of current and past centromeres (Extended Data Fig. 5).

**Extended Data Fig. 6:**
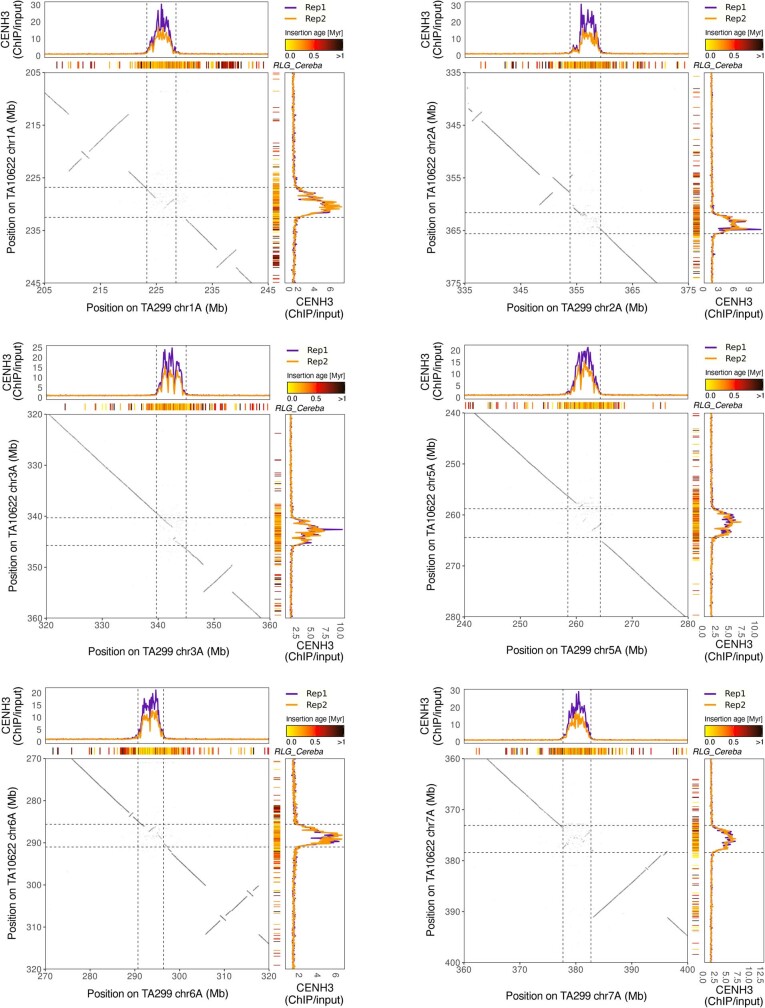
Dot plot comparisons of centromeric and peri-centromeric regions of *T. monococcum* accessions TA299 (horizontal) and TA10622 (vertical). Aligned with the dot plots are plots of the average coverage of CENH3 ChIP-Seq reads and positions of *RLG_Cereba* retrotransposon insertions colour-coded according to their insertion ages. The plot for chromosome 4A is shown in the main Fig. 2.

We found that functional einkorn centromeres are highly dynamic and evolving rapidly (Extended Data Figs. 5 and 6). This is in agreement with observations made in other grass species, indicating that centromeres can shift positions over time^23,41^. Sequence collinearity between TA299 and TA10622 was low or completely absent across functional centromeres, while chromosome segments adjacent to the functional centromeres aligned well (Extended Data Fig. 6). We found multiple inversions inside or in the immediate vicinity of centromeres (Fig. 2 and Extended Data Fig. 6), and we hypothesize that inversions are major drivers of centromere evolution. Inversions can displace parts of functional centromeres, resulting in centromere shifts.

The most notable example of a centromere shift was found on chromosome 4A, which was highly rearranged between TA299 and TA10622 (Fig. 2b, Extended Data Fig. 6 and Supplementary Fig. 11). TA10622 had one distinct region with a high density of *RLG_Cereba* insertions. The youngest of these insertions coincided with the highest CENH3 signals. By contrast, TA299 showed two regions with high *RLG_Cereba* insertion densities separated by around 10 Mb. The region with the youngest insertions overlapped with the current functional centromere (as identified by high CENH3 signals), whereas the second region contained a fragment of the ancestral centromere (Fig. 2b). We deduced a model to explain the differences in this centromere between TA299 and TA10622 (Fig. 2c). We propose that a series of inversions divided the ancestral centromere into two fragments, after which the centromere was re-established at the site of the larger ancestral centromere fragment in TA299 (Fig. 2c). A detailed comparison of around 400 kb of the new TA299 centromere with its (ancestral) counterpart in TA10622 showed that all new TE insertions in the new TA299 centromere were of the *RLG_Cereba* and *RLG_Quinta* family (Fig. 2d). On the basis of *RLG_Cereba* insertion ages, we estimated that this centromere shift in TA299 occurred between 20,000 and 100,000 generations ago (Extended Data Fig. 5). Moreover, an independent deletion of around 2 Mb in TA10622 was followed by a centromere shift of about 1 Mb (Fig. 2c). We found additional evidence for inversions in the centromeric regions of chromosomes 1A, 6A and 7A (Fig. 2e and Extended Data Fig. 5). In chromosomes 1A and 6A, *RLG_Cereba* and *RLG_Quinta* insertion sites and ages indicated that parts of functional centromeres had moved in both accessions around 500,000 and 300,000 generations ago, respectively (Extended Data Fig. 5). On chromosome 7A, an approximately 13 Mb segment was inverted, thereby removing about 1 Mb of the functional centromere in TA10622. We estimated that this event took place around 100,000 generations ago (Extended Data Figs. 5 and 6). In chromosomes 1A, 6A and 7A, centromeres appeared to be only partially impacted, resulting in a re-establishment of functional centromeres in the same chromosomal region (Extended Data Fig. 6).

Our data revealed a substantial number of centromere rearrangements. We propose that inversions can cause major centromere shifts if more than half of the functional centromere is displaced by an inversion (Fig. 2f,g). In such cases, the functional centromere is re-established in a new location (Fig. 2g), as illustrated with chromosome 4A. By contrast, when only small portions of a centromere are moved, the functional centromere appears to remain at or near its original position and its original size is re-established over time (Fig. 2h). This also supports a model of optimal centromere size as compromised centromeres appear to settle at a consistent size across chromosomes, despite significant disruption over time.

## Einkorn population genomics

To investigate einkorn genetic diversity and evolutionary history, we generated whole-genome sequencing data (around tenfold coverage) for a diversity panel comprising 219 einkorn accessions. We selected the constituent accessions of the panel to optimally represent diversity on the basis of geographical origin and genotyping data^42^, with 158 wild (124 α race, nine β race and 25 γ race) and 61 domesticated einkorn accessions (Supplementary Table 8). In total, 121,459,674 high-quality single-nucleotide polymorphisms (SNPs) were retained and we observed a low false-positive error rate of variant calling^43–46^ (Methods). Nucleotide diversity (*π*) was highest in the γ races (*π* = 0.0023) and similar across the other three groups (α, *π* = 0.0011; β, *π* = 0.0018; domesticated, *π* = 0.0014) (Supplementary Fig. 12a). Notably, but consistent with previous observations^13^, we did not observe a large reduction in nucleotide diversity in domesticated einkorn.

**Fig. 3:**
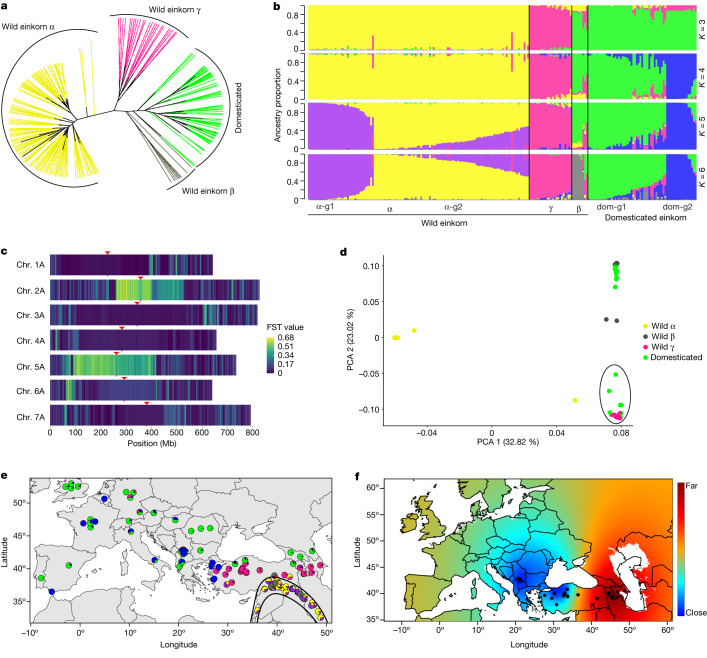
Einkorn population genomics. **a**, Unrooted neighbour-joining tree. **b**, Population structure (from *K* = 3 to *K* = 6). Each vertical bar represents one accession, and the bars are filled with colours representing the proportion of each ancestry. Einkorn groups were assigned considering *K* = 6 (on the basis of the cross-entropy value) based on the maximal local contribution of ancestry except for β (all β accessions were assigned as one group, regardless of the contribution of an ancestry). α group 1 (α-g1, *n* = 37) is shown in purple, α group 2 (α-g2, *n* = 87) is shown in yellow, γ (*n* = 24), β (*n* = 9), domesticated einkorn group 1 (dom-g1, *n* = 44) is shown in green and domesticated einkorn group 2 (dom-g2, *n* = 17) is shown in blue. A detailed list of accessions is provided in Supplementary Table 9. **c**, The mean fixation index (*F*_ST_) between the two domesticated einkorn groups calculated in 1 Mb sliding windows. Only accessions with 80% ancestry threshold at *K* = 4 were considered. Centromere midpoints are indicated by red arrowheads. **d**, PCA using only variants that are present on the introgressed segment on chromosome 5A. Accessions were coloured according to the structure analysis in **b**. Circled accessions include wild γ accessions and some domesticated einkorn accessions. **e**, The geographical location of einkorn collection sites. The colours in pie charts correspond to the ancestry at *K* = 6. The Fertile Crescent is indicated by black lines. Only accessions with known collection sites are shown. **f**, Geographical projection of the first PCA axis for γ accessions on the basis of the introgressed segment on chromosome 2A (this analysis was performed excluding α and β accessions). The black dots represent the location of γ accessions. Blue colour represents the collection sites of γ accessions that were genetically the least diverged from the γ introgression found in domesticated einkorn. The Karacadağ region (K) is indicated on the map.

Phylogeny and principal component analysis (PCA) confirmed that wild einkorn clusters into α, β and γ races^13,42^ (Fig. 3a and Supplementary Fig. 12b). The domesticated einkorn accessions clustered together with race β, most of which were collected in the Karacadağ area in southeastern Turkey (Supplementary Table 8). This supports the hypothesis that einkorn was domesticated from a small and restricted wild population closely related to present-day β accessions.

We estimated the ancestry coefficient for each accession to examine the evolutionary history of einkorn. Consistent with the PCA, the three groups of wild einkorn races separated into three distinct clusters at *K* = 3 (where *K* is the number of putative ancestral populations), and domesticated einkorn grouped with the β accessions (Fig. 3b). At *K* = 4, domesticated einkorn split into two separate groups (Fig. 3b and Supplementary Fig. 13), which was not observed in a previous analysis using genotyping-by-sequencing data^42^. The cross-entropy values reached a plateau starting at *K* = 6 (Supplementary Fig. 14). Compared with *K* = 4, race β separated from domesticated einkorn, whereas wild α race accessions formed two distinct groups at *K* = 6 (Fig. 3b and Supplementary Table 9).

**Extended Data Fig. 7:**
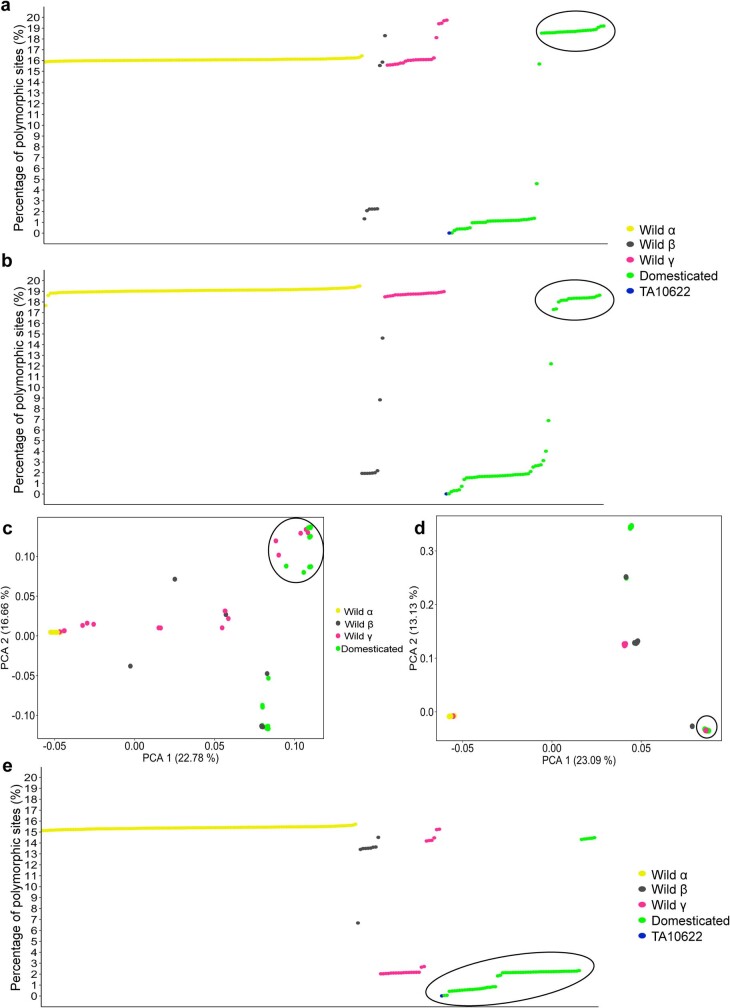
Population genomic analyses on diverged genomic regions. (**a**-**b**), The percentage of polymorphic sites of each einkorn accession compared to the TA10622 assembly considering SNPs present only in the two highly diverged genomic segments on chromosomes 2A (**a**) and 5A (**b**). The circles highlight domesticated einkorn accession that diverged from the TA10622 reference assembly. (**c**–**d**), Principal component analyses (PCA) based on SNPs found only in the two large introgressed segments on chromosomes 2A (**c**) and chromosome 7A (**d**) revealed that γ accessions cluster with some domesticated einkorn accessions. The circles highlight domesticated einkorn accessions that cluster with wild γ accessions instead of β. **e**, The percentage of polymorphic sites compared to the TA10622 assembly of the introgressed region on chromosome 7A showed that the majority of the domesticated einkorn accessions (highlighted in the circle) are not diverged from TA10622, which also carries the introgression on chromosome 7A.

To test for admixture and whether specific genomic segments contributed to the split of domesticated einkorn, we estimated genetic differentiation (*F*_ST_) between the two domesticated einkorn groups in sliding windows across the seven chromosomes, revealing two large segments that were highly differentiated between the two groups. These two blocks spanned the centromeric and pericentromeric regions of chromosomes 2A (around 266 Mb) and 5A (around 329 Mb) (Fig. 3c). Divergence analyses across these two segments confirmed a strong separation of domesticated einkorn accessions (Extended Data Fig. 7a,b). We performed PCA considering only variants that were located within these two diverged segments, revealing the clustering of some domesticated einkorn accessions with wild γ rather than β accessions (Fig. 3d and Extended Data Fig. 7c). These results suggest an introgression of genetic material from race γ into the domesticated einkorn gene pool. Pericentromeric regions show low recombination frequency in wheat^24,47,48^, explaining why they can persist as large blocks.

To obtain a more complete estimate of the proportions of γ introgressions in domesticated einkorn, we performed pairwise comparisons of nucleotide diversity across chromosomes between one γ accession and each of the domesticated einkorn accessions. In addition to the two large segments on chromosomes 2A and 5A, we determined that most (92%) domesticated einkorn accessions carry an approximately 150 Mb γ genomic segment in the pericentromeric region of chromosome 7A (Extended Data Fig. 7d,e, Supplementary Fig. 15 and Supplementary Tables 10 and 11). Owing to its high frequency in the domesticated einkorn gene pool, we did not detect this segment by *F*_ST_ analysis. TreeMix^49^ analysis supported the influx of genetic material from wild einkorn race γ into the domesticated einkorn gene pool (Supplementary Fig. 16). Overall, we estimate that the introgressions from race γ accounted for an average of 6.7% (range 0.3–13.1%) of the domesticated einkorn genome (Supplementary Table 10), resulting in an increased nucleotide diversity within the domesticated gene pool (Supplementary Fig. 17).

**Extended Data Fig. 8:**
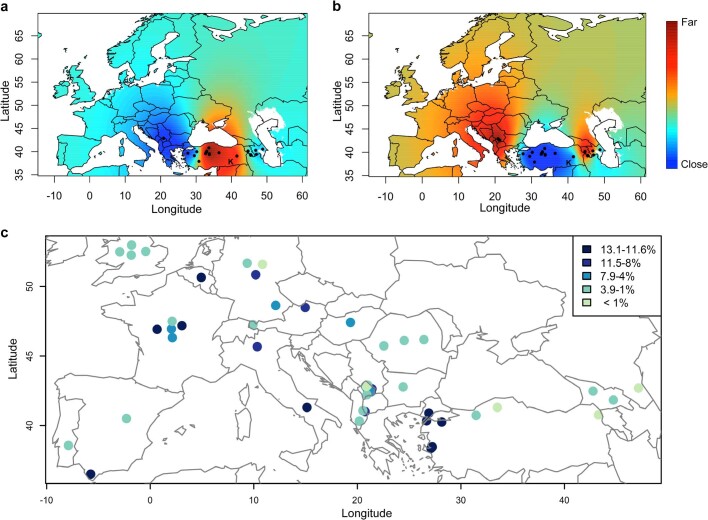
Wild einkorn γ race introgression into domesticated einkorn. **a**, Geographical projection of the second PCA axis based on variants found only in the large introgressed segment on chromosomes 5A. **b**, Geographical projection of the first PCA axis based on variants found only in the large introgressed segment on chromosomes 7A. Blue colour indicates γ accessions with close genetic relatedness to the introgressed segments found in domesticated einkorn accessions. Black dots represent the coordinates of γ accessions. The analysis was done excluding α and β accessions. Maps in both a and b were created using the Kriging function in the fields v10.3 R package (https://cran.r-project.org/web/packages/fields). **c**, The proportion of γ race introgression in domesticated einkorn. Each dot represents the coordinates of a domesticated einkorn accessions. Dark blue and light green represent the highest and the lowest proportions of γ introgressions in domesticated einkorn, respectively (legend at top right). The map was created using the graphic plot function in R.

Wild einkorn accessions found in the Fertile Crescent mainly belong to race α, with race β being restricted to an area around the Karacadağ mountains. Race γ is not present in the Fertile Crescent^13,42^ and is mainly found in central and northwestern Turkey (Fig. 3e). We hypothesize that the probable geographical site of the γ introgression can be inferred by comparing the genetic relatedness of the introgressed segments in domesticated einkorn to wild γ accessions. The geographical projection of the first and the second PCA axes using the introgressed segments identified one group of γ accessions from central and northwestern Turkey that showed the closest genetic relatedness to the introgressed segments in domesticated einkorn. We propose that this geographical region, which is several hundred kilometres away from the site of einkorn domestication, is the most likely region where the hybridization between ancestral domesticated einkorn and a wild γ accession might have occurred (Fig. 3f and Extended Data Fig. 8a,b).

From the Fertile Crescent, cultivation of domesticated crops rapidly expanded following two main migration routes: one to the west into central Turkey (and later Europe) and a second to the east into Transcaucasia^50^. Notably, the domesticated einkorn accessions with the highest proportions of γ introgression were collected from western Turkey (coinciding with the most likely site of hybridization), whereas domesticated einkorn accessions collected in eastern Turkey and Georgia had a very low proportion of γ introgressions (Extended Data Fig. 8c and Supplementary Table 10). This pattern may reflect the different migration routes of domesticated einkorn. After domestication in the Karacadağ region, einkorn was most likely cultivated in close proximity to wild γ populations in central and northwestern Turkey, leading to the influx of genetic material from race γ. By contrast, the domesticated einkorn populations that were moved to the east were not impacted by this hybridization, which would explain the low proportion of γ introgressions in domesticated einkorn accessions from eastern Turkey and Georgia. The apparent lack of a strong domestication bottleneck in domesticated einkorn was explained with a ‘dispersed-specific’ model of einkorn domestication, including multiple domestication events from geographically dispersed wild β populations^13^. A hallmark of domesticated einkorn is the non-fragile rachis. In our diversity panel, all domesticated einkorn accessions had the same haplotype in the *non-brittle rachis1* (*btr1*) gene, including a critical alanine to threonine amino acid substitution^4^, indicating that this key domestication gene has a single origin in domesticated einkorn. The lack of a strong diversity reduction in domesticated einkorn could therefore also be the result of gene flow after domestication, as demonstrated for the introgressions from wild γ accessions. Recent population and pan-genome analyses confirmed that hybridizations had an important role in increasing genetic diversity in wheat after domestication^23,51^. The introgression of genetic material from wild γ accessions may have had an important role in the adaptation of domesticated einkorn to new climatic conditions outside the Fertile Crescent.

## Einkorn introgressions into bread wheat

**Fig. 4:**
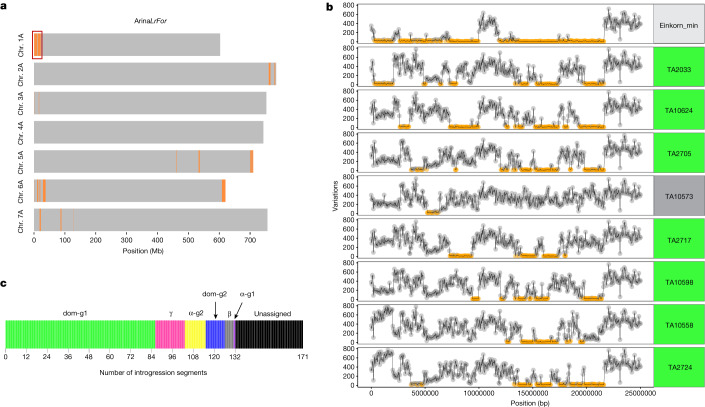
Einkorn introgression into bread wheat. **a**, Einkorn introgressions (highlighted in orange) into Arina*LrFor* identified using the *k*-mer variation approach (IBSpy; Supplementary Note 2). The red square on chromosome arm 1AS corresponds to the region shown in detail in **b**. **b**, IBSpy variations between Arina*LrFor* (chromosome 1A, position 0–25 Mb) and einkorn. Regions with variation scores of ≤30 (identical by state) are indicated in orange, corresponding to einkorn introgressions. Einkorn_min represents a consensus that shows the lowest variation scores across all resequenced einkorn accessions. The remaining plots illustrate the variation scores between Arina*LrFor* and eight selected einkorn accessions. Accession names highlighted in green and grey belong to domesticated groups 1 (dom-g1) and β, respectively. **c**, The number of introgression segments that could be assigned to a particular einkorn group.

**Extended Data Fig. 9:**
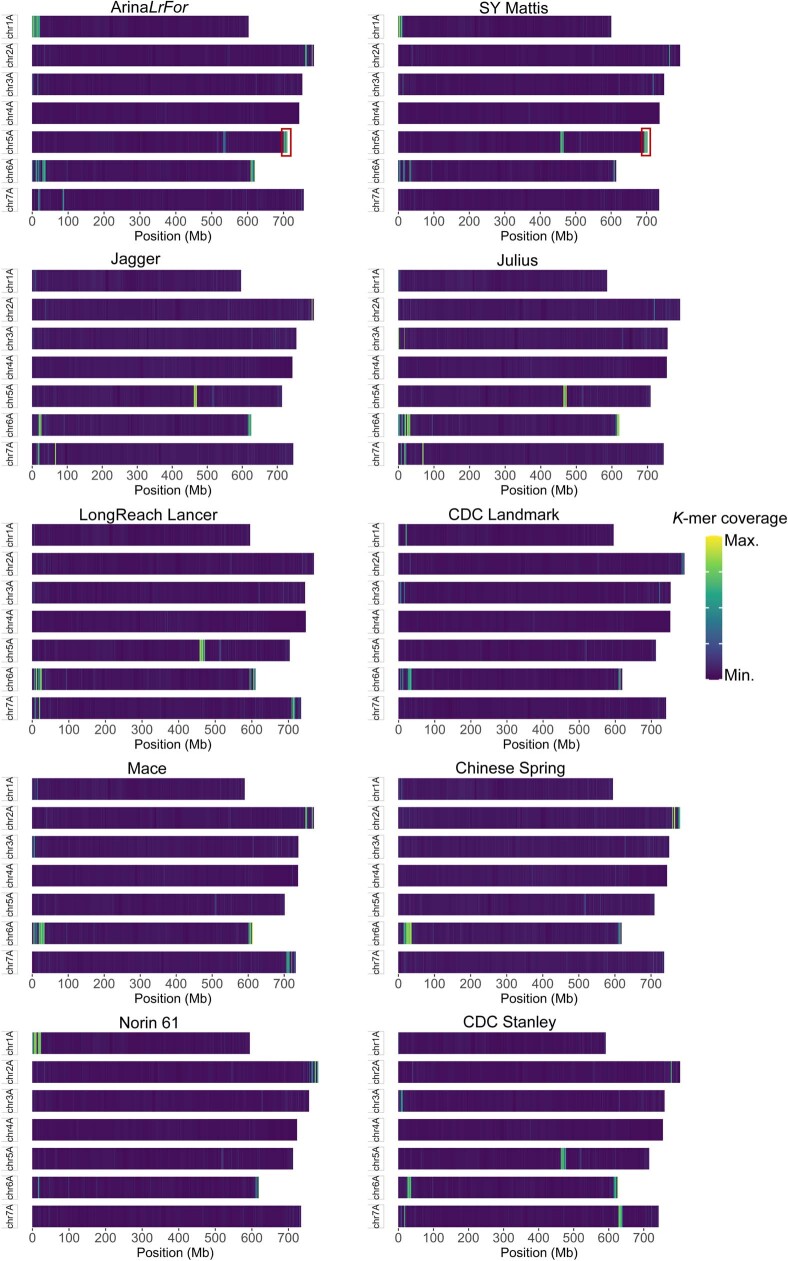
Einkorn introgression into 10 chromosome-scale bread wheat assemblies based on *k*-mer mapping approach. Putative introgressions are identified as regions with increased coverage of mapped *k*-mers from *T. monococcum* and visualized in the blue–yellow heat map (legend at the right). Red squares around chromosome 5AL in both Arina*LrFor* and SY Mattis indicate the *Yr34*-carrying region that was used as control. Source data

Although einkorn is not the donor of the hexaploid bread wheat A subgenome, several einkorn introgressions have been described in hexaploid wheat^6,9,52–54^. To gain a comprehensive estimate of the proportions of einkorn introgressions in the modern bread wheat gene pool, we used *k*-mer-based approaches to detect einkorn introgressions in ten chromosome-scale bread wheat assemblies^23,24^ (Supplementary Note 2). For a positive control, we tested a known einkorn translocation carrying the *Yr34* stripe-rust-resistance gene located at the distal end of chromosome arm 5AL in bread wheat lines Arina*LrFor* and SY Mattis^6^. We detected an approximately 8–10 Mb einkorn segment at the expected position in the two wheat lines (Fig. 4a, Extended Data Fig. 9 and Supplementary Table 12), supporting the idea that our *k*-mer-based approaches are suitable to detect einkorn introgressions. On average, we determined that the bread wheat A subgenomes contain around 1% of einkorn introgressions, ranging from 0.7% in cultivar CDC Landmark to 1.9% in Arina*LrFor* (Fig. 4a and Supplementary Table 12).

We identified 171 einkorn segments with a cumulative size of 472.8 Mb across the 10 bread wheat cultivars. The average segment size was 2.8 Mb, ranging from 50 kb to 16.7 Mb. In most cases, the introgressed einkorn segment could not be assigned to a single einkorn accession from our diversity panel. Instead, different regions of an introgressed segment showed close relatedness to various einkorn accessions of the diversity panel (Fig. 4b), suggesting that the direct donor of the introgressions was not present in the panel. However, 132 out of the 171 introgressed segments (with a cumulative size of 431.8 Mb) could be assigned to a specific group of einkorn accessions. The majority of the 132 segments with a clear origin (86 segments with a cumulative size of 287.1 Mb) were assigned to domesticated einkorn group 1 (dom-g1) (Fig. 4c and Supplementary Table 12). The remaining segments originated from the other domesticated and wild einkorn groups (Fig. 4c). These results indicate that most of the einkorn introgressions in the elite bread wheat genome originated from hybridizations between ancient tetraploid or hexaploid wheats and domesticated einkorn.

## Mapping of a plant architecture gene

**Fig. 5:**
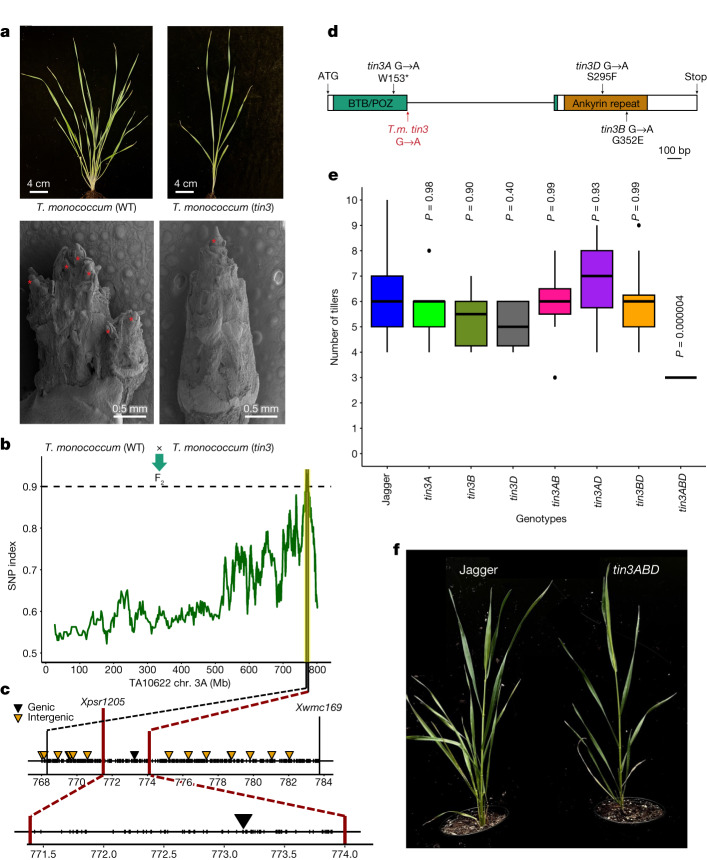
Positional cloning of *tin3* and translational research in hexaploid bread wheat. **a**, Phenotypes of wild-type *T. monococcum* accession TA4342-L96 (top left) and *tin3* (top right) at the tillering stage. Bottom left, scanning electron microscopy (SEM) images of seedlings showing primary shoot bud and axillary tiller bud formation (indicated by asterisks) in TA4342-L96 after leaf removal at the eight-leaf stage. Bottom right, SEM image of a seedling showing only the primary bud (indicated by an asterisk) at the shoot apex with no axillary buds in the *tin3* mutant after leaf removal at the six-leaf stage. The SEM experiment was repeated three times. **b**, The SNP index in a mutant *tin3* bulk (*n* = 30 F_2_ plants) across einkorn chromosome 3A. The TA10622 reference assembly was used for read mapping. **c**, The *tin3* target interval in TA10622. *Xpsr1205* and *Xwmc169* indicate the positions of previously identified *tin3*-flanking markers. The triangles indicate the positions of EMS-induced point mutations. **d**, *Tin3* (*Tm.TA10622.r1.3AG0164370*) gene structure. The boxes represent exons and the line represents the intron. The G to A point mutation in *tin3* is indicated by a red arrow. The locations of SNPs found within the Jagger TILLING population for all three homeologous *tin3* copies is indicated in black. *T.m*., *T. monococcum*. **e**, Tiller numbers in bread wheat cultivar Jagger (*n* = 20), *tin3A* (*n* = 12), *tin3B* (*n* = 6), *tin3D* (*n* = 14), *tin3AB* (*n* = 7), *tin3AD* (*n* = 8), *tin3BD* (*n* = 12) and *tin3ABD* (*n* = 8). All eight *tin3ABD* triple mutants developed exactly three tillers. The box boundaries indicate the first and third quartiles. The lines extending from the boxes (whiskers) indicate the variability outside the lower and upper quartiles. The lines in the middle of the boxes represent the median values of *π*. Outliers are plotted as individual points. *P* values were calculated using two-sided Tukey’s honest significant difference tests, comparing with Jagger. **f**, Representative images showing the tillering phenotypes of Jagger (left) and *tin3* triple mutants (right).

Diploid einkorn can be used as a model to map and clone agriculturally important genes and to translate this knowledge into polyploid bread wheat breeding. A diploid model species is particularly useful to clone recessive genes, of which the phenotypic effects are masked in a polyploid. Here we used the einkorn genomic resources to map the *tiller inhibition* (*tin3*) gene. Tillering is a key shoot architecture trait in cereals, contributing to spike number and grain yield. *tin3* was originally identified as a recessive ethyl-methanesulfonate (EMS)-induced mutation in the domesticated einkorn accession TA4342-L96. The *tin3* mutant showed a reduced tiller number (Fig. 5a) and the causal gene was mapped to chromosome arm 3AL^55,56^. To further map *tin3*, we used a MutMap-based approach^57^ with the TA10622 reference assembly to identify EMS-induced point mutations associated with *tin3*. MutMap and the physical positioning of previously identified *tin3-*flanking markers^56^ revealed an approximately 2.5 Mb target interval, in which only one EMS-type (G to A) point mutation was found (Fig. 5b,c). This SNP disrupted the exon–intron junction of the gene *Tm.TA10622r1.3AG0164370*, resulting in intron retention and the formation of an aberrant protein (Fig. 5d). A kompetitive allele-specific PCR (KASP) marker derived on this SNP co-segregated with the *tin3* phenotype in a ‘TA4342-L96 × *tin3*’ mapping population of 750 F_2_ gametes. *Tm.TA10622r1.3AG0164370* encodes a putative co-transcription factor with an N-terminal BTB/POZ domain and an ankyrin-repeat domain at the C terminus. The *tin3* candidate is orthologous to the *Uniculme4* (*Cul4*) gene that controls tillering in barley^58^. We next identified mutations in the *tin3* bread wheat orthologues using a hexaploid wheat TILLING population^59^ (Fig. 5d). Although bread wheat mutants containing point mutations in only one or two of the *tin3* homeologues showed normal tillering (Fig. 5e,f), triple mutants affecting all three bread wheat subgenomes showed a significant decrease in tiller number (Fig. 5e,f). In summary, the einkorn genomic resources facilitated rapid identification of the gene underlying the *tin3* mutant in both diploid and polyploid wheat. We demonstrate that the knowledge gained in genetics from diploid wheat can be rapidly transferred to hexaploid bread wheat biology and improvement.

## Methods

### Plant materials

The plant material used in this study was selected from a collection of 733 accessions of wild and domesticated einkorn held at the Wheat Genetics Resource Center (WGRC) at Kansas State University^42^. To generate reference assemblies, we selected one domesticated einkorn accession (*T. monococcum* L. subsp*. monococcum*; TA10622) collected in Albania and one wild accession (*T. monococcum* L. subsp. *aegilopoides*; TA299) collected in northern Iraq. These two accessions were selected on the basis of genotyping-by-sequencing data^42^ as representative accessions within the respective clade (domesticated and wild race α), had low heterozygosity and available passport information. For whole-genome sequencing, a panel of 219 accessions (Supplementary Table 8) was selected on the basis of diversity and geographical origin^42^.

### Reference genome sequencing, assembly, and validation

#### PacBio HiFi library preparation and sequencing

High-molecular-mass (HMM) genomic DNA was isolated from young leaves obtained from 3-week-old seedlings after dark treatment for 48 h. DNA was extracted according to a HMM DNA extraction protocol for long-read sequencing^60^. DNA quantification was performed using the Qubit dsDNA HS Assay (Q32851, Thermo Fisher Scientific), purity was confirmed using the Nanodrop spectrophotometer by checking the 260/280 and 260/230 ratios, and the DNA size was validated by using the FemtoPulse system (Agilent). HiFi libraries were then prepared according to the manual ‘Procedure & Checklist - Preparing HiFi SMRTbell Libraries using the SMRTbell Express Template Prep Kit 2.0’ (PN 101-853-100, Pacific Biosciences) with 10 µg DNA sheared by using the Megaruptor 2 system (Diagenode) to obtain a 15–20 kb average size. Size-selected libraries were sequenced on the PacBio Sequel II system in CCS mode for 30 h. For each accession, we obtained 121 Gb PacBio HiFi reads, corresponding to a coverage of around ~21-fold (Supplementary Table 13).

#### Bionano optical map

Einkorn grains were germinated on filter paper in the dark for 4 days at 4 °C followed by 3 days at 25 °C. Ultra HMM DNA was isolated from fresh root meristem tissue using the Plant DNA Isolation Kit protocol (Bionano Genomics). Labelling was performed using direct labelling enzyme (DLE1) and staining of the HMM DNA according to the Bionano Prep Direct Label and Stain (DLS) protocol (30206-Bionano Genomics). Optical maps were generated using the Bionano Genomics Saphyr System (Saphyr Chip G1.2) according to the Saphyr System User Guide (3024-Bionano Genomics). Data processing was performed using the Bionano Solve v.3.6 software (https://bionanogenomics.com/support/software-downloads).

#### Omni-C library construction and sequencing

Omni-C libraries were prepared using the Dovetail Omni-C Kit for plant tissues according to the manufacturer’s protocol. In brief, chromatin was fixed in place in the nucleus from 100 mg of dark-treated young leaves. An in situ digestion of the fixed chromatin was performed using an endonuclease enzyme. Chromatin was then released by lysing the cells. Chromatin ends were repaired and ligated to a biotinylated bridge adapter to capture contacts, before proximity ligation of the adapter-containing ends. After proximity ligation, cross-link-reversal and DNA purification from proteins was performed. The purified DNA was treated to remove biotin that was not internal to ligated fragments. Two sequencing libraries were generated using Illumina-compatible adapters for each accession. Biotin-containing fragments were isolated using Streptavidin beads before PCR enrichment of the library. The two libraries were sequenced on the Illumina HiSeq X platform. Around 400 million paired-end reads (2 × 150 bp) were generated.

#### Genome assembly

PacBio HiFi reads were assembled using hifiasm (v.15.1)^61^ with the default parameters (https://github.com/chhylp123/hifiasm/) to generate primary contig assemblies. We generated hybrid scaffolds by combining contig assemblies and optical maps using the hybridScaffold pipeline (Bionano Solve v.3.6) with the default parameters. We then integrated Omni-C read data to produce pseudomolecule assemblies using Juicer (v.1.6; https://github.com/aidenlab/juicer)^62^ and the 3D-DNA pipeline (https://github.com/aidenlab/3d-dna)^63^. First, to generate Hi-C contacts (with duplicates removed), Omni-C Illumina short reads were processed with juicer.sh (parameter: -s none). The output file ‘merged_nodups.txt’, the hybrid scaffolds and contigs that were not integrated into hybrid scaffolds (in one fasta file) were then used to produce an assembly with 3D-DNA^63^ (using run-asm-pipeline.sh with -r 0 parameter). We used Juicebox (v.1.11.08)^64^ to visualize the Hi-C contact matrix, and to manually curate the assembly (orient and order hybrid scaffolds and pseudomolecules). The final Hi-C contact maps and assemblies were saved using run-asm-pipeline-post-review.sh from the 3D-DNA pipeline.

#### Assembly validation and quality control

We validated the two einkorn assemblies by mapping PacBio HiFi reads (with minmap2 v.2.21), Illumina short reads (with bwa mem v.0.7.17) and the optical map (Bionano Solve v.3.6) to the final assemblies and we found no major discrepancies. Assemblies were further validated using a genetic map constructed from a recombinant inbred line (RIL) population (see below). Using the genetic map, we manually corrected three misorientations in the telomeric regions (chromosomes 2A and 4A in TA299 and chromosome 2A in TA10622), and introduced a 1.04 Mb segment into chromosome 4A of TA10622 that was placed in the unanchored chromosome (Supplementary Table 14). We revalidated the corrected assembly by (1) re-calling SNPs from the RILs, (2) mapping raw-reads and optical maps and (3) mapping the individual contigs to both assemblies to ensure correct orientation of the regions. Assembly completeness was evaluated using BUSCO (v.5.0.0)^65^ with the plant dataset (poales_odb10). Moreover, we generated Illumina short reads (150 bp paired-end reads, ~40-fold coverage) from leaf tissues of TA299 and TA10622 to evaluate the assemblies. Merqury (v.1.3)^66^ was used to estimate the assembly consensus quality (QV) and completeness on the basis of the comparison of *k*-mers.

### Genetic map construction

#### RIL population

We constructed a genetic map using a recombinant inbred line (RIL) population consisting of 827 lines resulting from a cross between a wild (TA291, also identified as TA4342_L95) and a domesticated (TA10868, also identified as TA4342_L96) einkorn accession. The two parents of the RIL population were sequenced at high depth (9.1-fold) using a TruSeq library, whereas the RILs were sequenced using a low-coverage (0.03-fold) skim-sequencing (skim-seq) protocol that used a low-volume Illumina Nextera library^67^. In the skim-seq panel, we also included five replicates of each parent.

#### Marker discovery and genotyping of RILs

Both the TruSeq and Nextera libraries were sequenced on the Illumina HiSeq X10 system with 2 × 150 bp reads (Psomagen). We used custom Perl scripts for demultiplexing raw FASTQ files (https://github.com/sandeshsth/SkimSeq_Method) obtained from Nextera sequencing^67^ and TruSeq (https://github.com/sandeshsth/Fastq). Adapters and primers were trimmed using fastp^68^. Trimmed high-quality reads from the two parents were aligned to the TA299 and TA10622 assemblies separately using SAMtools^69^ (v.1.8) and variants were called using BCFtools (v.1.9)^70^. Variants were filtered for minimum and maximum filtered read depths of ≥6 and ≤100, respectively, and reference and alternative allele depths of ≥3. Missing and heterozygous genotypes called in either RIL parents were removed. The remaining homozygous SNPs were then called on the RIL population^67^. Owing to the low sequence coverage, we used a bin mapping approach in 1 Mb sliding windows to call consensus genotypes^71^. Genotypes called on RILs were coded according to the parental SNPs replacing the genotypes as either wild (P1) or domesticated (P2). A consensus genotype was called within the 1 Mb sliding windows on the basis of the proportions of P1 and P2 within the window. If P1/P2 ≥ 0.7, then we coded the window as P1, if P2/P1 ≥ 0.7 we coded the window as P2, otherwise as heterozygous (H). A custom Python script (Data availability) was used to genotype the 1 Mb windows and to identify the recombination breakpoints. The genotyping file with filtered recombination bins for missing and heterozygous loci and individual RILs was used to construct the genetic linkage maps.

#### Genetic linkage map construction

The genetic linkage maps were constructed using JoinMap (v.5.0; https://www.kyazma.nl/index.php/JoinMap/) using the bins as markers. We filtered the markers for missing data (removed >30% missing) at the population level. In JoinMap, we removed identical markers (similarity = 1) and mapped only one marker of the identical pair. We grouped the markers using minimum LOD of 6 and the markers were mapped using a regression mapping approach and the Kosambi function. The linkage maps were visualized using Mapchart (v.2.32; https://www.wur.nl/en/show/mapchart.htm). Linkage maps were constructed using this approach with both wild and domesticated einkorn assemblies.

#### Comparing genetic maps to TA299 and TA10622 assemblies

We visually compared the marker order of the genetic map to the two einkorn assemblies and corrected discrepancies if all of the following three criteria were met: (1) The genetic distance between two mis-ordered loci was ≥0.5 cM to ensure it is not a mistake in the genetic map; (2) the correction does not require breaking of a hybrid scaffold; and (3) the reorientation was supported by the mapping of raw reads and the optical maps.

### Genome annotation

#### Transcriptome sequencing

Around 100 mg of frozen and ground tissues from roots, whole aerial parts at the seedling stage, flag leaves, fully emerged spikes, glumes and grains were used for RNA isolation using the Maxwell RSC Plant RNA Kit (AS1500) and the Maxwell RSC 48 instrument according to the kit protocol (Promega). For RNA-seq, around 10 Gb of Illumina 150 bp paired-end reads were generated for each tissue. PacBio Iso-seq SMRTbell libraries were constructed according to the standard isoform sequencing protocol (Pacific Biosciences, 101-763-800). Full-length complementary DNA was synthesized from total RNA from the six tissues separately using NEBNext Single Cell/Low Input cDNA Synthesis & Amplification Module (New England Biolabs, E6421S). The ProNex Size-Selective Purification System (Promega, NG2001) was used for size selection. One SMRT Cell 8M (Pacific Biosciences, 101-389-001) was sequenced on the PacBio Sequel II system using the Sequencing Kit 2.0 (Pacific Biosciences, 101-820-200).

#### Gene model prediction

For both TA299 and TA10622, gene model prediction was performed according to a previously described method^72^ with minor modifications, combining transcriptomics data, ab initio prediction and protein homology. First, TA299 and TA10622 RNA-seq data from the six tissues were mapped to their respective reference assemblies using STAR^73^ (v.2.7.0f; parameters: --outFilterMismatchNoverReadLmax 0.02) and assembled into transcripts with StringTie^74^ (v.2.1.4; parameters : --rf -m 150 -f 0.3 -t). Iso-seq data were mapped using minimap2^75^ (v.2.21; parameters: -ax splice -uf –secondary=no -C5) and the redundant isoforms were further collapsed into transcript loci using cDNA_Cupcake (v.12.4.0; http://github.com/Magdoll/cDNA_Cupcake; parameters: --dun-merge-5-shorter). The RNA-seq and Iso-seq transcripts were merged using StringTie^74^ (v.2.1.4; parameters: --merge -m 150) for each accession into a pool of candidate transcripts and Transdecoder (v.5.5.0; https://github.com/TransDecoder/TransDecoder) was used to find potential open reading frames and to predict protein sequences within the candidate transcript set. For the ab initio gene predictions, we used BRAKER2 (v.2.1.2)^76^ and FgeneSH (v.8.0.0; http://www.softberry.com). In brief, BRAKER2 gene prediction was trained supported by RNA-seq and Iso-seq data (parameters: --softmasking --gff3 --cores=48 --nocleanup --bam=‘list of BAM files’). For the FgeneSH prediction, the TA299 and TA10622 pseudomolecules were repeat masked using a de novo repeat library constructed using the EDTA pipeline^77^ and the TREP database^78^ (v.19). FgeneSH annotation was performed using the monocot matrix for the gene prediction. For the protein homology evidences, we used the translated proteins from gene annotations of *T. urartu*^79^, *Aegilops tauschii*^80^, wild emmer (Zavitan)^81^, hexaploid wheat (Kariega^72^ and ArinaLrFor^23^), barley (Morex v.3)^82^, the related grass species *Brachypodium distachyon*^36^ and rice^83^, and the Triticeae and Poaceae protein sequences downloaded from the UniProt database (2021_03). All protein sequences were mapped against the TA299 and TA10622 assemblies using GenomeThreader^84^ (v.1.7.1; parameters: -startcodon -finalstopcodon -species rice -gcmincoverage 70 -prseedlength 7 -prhdist 4 -gff3out). We used EVidenceModeler^85^ (v.1.1.1) to join all of the gene evidences from transcriptomics, ab initio predictions and protein alignments with weights adjusted according to the input source (FgeneSH = 2; BRAKER2 = 1; protein homology = 6; transcriptomics = 12). Finally, we performed two rounds of isoform and UTR prediction using the PASA pipeline (v.2.5.1)^86^ with the default parameters. Gene models were classified into high and low confidence according to the classification criteria used by the International Wheat Genome Sequencing Consortium^24^ and a previous study^82^. In brief, protein-encoding gene models were considered to be complete when start and stop codons were present. A comparison against PTREP^78^, UniPoa (Poaceae database of annotated proteins from UniProt_2021_03) and UniViri (Viridiplantae database) was performed using DIAMOND^87^ (v.2.0.9) and a BUSCO (v.5.2.2) analysis against the poales database (v.10; parameters: -m prot -c 20 -l poales). Gene candidates were further classified using the following criteria: a high-confidence (HC) gene model is complete with a hit in the UniViri database and/or in UniPoa and/or BUSCO poales database and not PTREP. A low-confidence (LC) gene model is incomplete and has a hit in the UniViri or UniPoa or BUSCO poales database but not in PTREP, or the protein sequence is complete with no hit in UniViri, UniPoa, BUSCO poales and PTREP. Putative functional annotations were assigned to HC and LC transcripts using a protein comparison against the UniProt database (2021_03) and PFAM domain signatures and Gene Ontology were assigned using InterproScan^88^ (v.5.55–88.0).

### Circos and synteny

Dot plot comparisons were performed using chromeister^89^. For circos, we performed a BLAST search using HC protein sequences with DIAMOND^87^ (v.2.0.9; parameter -e 1e-10) and syntenic blocks were identified with MCScanX^90^ between TA299 and TA10622. Only relationships between the same chromosomes were retained. The Circos plot was generated using the Circos software^91^. The density of genes and repeat elements as well as CENH3 read coverage shown in the ciros plot were calculated in non-overlapping 10 Mb windows.

### Identification of 1 Mb tandem duplication

The 1 Mb tandem duplication was initially identified because of a conflict in the long arm of chromosome 4A between the contig-level assembly of TA10622 and the hybrid scaffolds after integrating the optical map. The contig-level assembly suggested an approximately 1 Mb tandem duplication that was collapsed in the optical map. Manual inspection revealed that multiple PacBio reads spanned the putative duplication breakpoints (Supplementary Fig. 18a–c). We designed a primer pair across the junction between the duplicated segments, which amplified in TA10622, but not in TA299 (Supplementary Fig. 18d), confirming that the tandem duplication in TA10622 is real. We manually corrected the disagreement based on the genetic map and read mapping, which supported the contig-level assembly. We defined the breakpoints to the base-pair level and analysed the sequences located at the breakpoints using Gepard (v.1.40)^92^ by generating dot plots. A PCR marker (forward primer, 5′-GGTCCCAGGCCATGATACCTC-3′; reverse primer, 5′-CTATGTCTCCCACGTGTCGAGGT-3′) was developed to validate the presence of the duplication in T10622. The amplicon (361 bp) was verified by Sanger sequencing. To inspect the sequence similarity between the two segments, we aligned one segment (558331155–559371848 bp) against the other (557272410–558331154 bp) using minimap2 (v.2.21)^75^, and we counted the number of SNPs and the covered sequences in 5 Mb genomic windows. We assessed the einkorn diversity panel for the duplication using whole-genome alignments and analysed the read depths at the MADS-box gene from sequencing reads of all 218 einkorn accessions using SAMtools^69^ depth command (v.1.8). The variant frequency between the duplicated segments was above the HiFi sequence read accuracy (mean read QV = 30), an important factor that probably enabled the differentiation and assembly of the two highly similar segments.

### Centromere analysis

#### ChIP–seq

ChIP was performed according to a described previously method^93^, standardized with anti-wheat-CENH3 antibodies^94^. An antigen with the peptide sequence ‘RTKHPAVRKTKALPKK’ corresponding to the N terminus of wheat CENH3 was used to produce the antibody using the custom-antibody production facility provided by Thermo Fisher Scientific. In total, 0.396 mg of customized antibody was purified and obtained as a pellet. The pellet was dissolved in 2 ml of PBS buffer, pH 7.4 resulting in 198 ng μl^−1^ of CENH3 antibody. Antibodies against H3K4me3 (04-475) were purchased from Sigma-Aldrich. The specificity of the anti-CENH3 antibodies was validated using immunofluorescence assays on mitotic and meiotic chromosomes of diploid (*T. monococcum*) and hexaploid (*T. aestivum*) wheat. Nuclei were isolated from 2-week-old seedlings and digested with micrococcal nuclease (Sigma-Aldrich) to liberate nucleosomes. The digested mixture was incubated overnight with 3 μg of antibody at 4 °C. Target antibodies were captured from the mixture using Dynabeads Protein G (Invitrogen) to obtain ChIP DNA. Mock DNA control was maintained with the input DNA using the same conditions as described above without antibodies. The ChIP experiments were performed in two biological replicates. Library construction was performed using the TruSeq ChIP Sample Prep Kit (Illumina) according to the manufacturer’s instructions.

#### PacBio DNA methylation analysis

Methylation in the CpG context for TA299 and TA10622 was inferred with ccsmeth (v.0.3.2)^95^, a deep-learning method to detect DNA 5mCpGs using kinetics features from PacBio CCS reads. The methylation prediction for CCS reads was called using the model ‘model_ccsmeth_5mCpG_call_mods_attbigru2s_b21.v1.ckpt’ and then aligned to their respective genome using BWA (v.0.7.17)^96^ and reads were filtered for hard/soft clips and quality (MAPQ ≥ 60) using SAMtools (v.1.8)^69^. The methylation frequencies were calculated at the genome level from their respective modbam files and using the aggregate mode of ccsmeth with the model ‘model_ccsmeth_5mCpG_aggregate_attbigru_b11.v2.ckpt’. To generate meta plots of *RLG_Cereba* elements, the bedmethyl file resulting from methylation frequency call was converted to bedgraph format. The bedgraph file was converted to bigwig using the script bedGraphToBigWig^97^. CpG methylation for *RLC_Cereba* copies was then calculated using the deeptools computeMatrix function and visualized using the deeptools plotProfile function^98^.

#### ChIP–seq data analysis

Raw ChIP and input control sequencing reads were quality filtered and adapter sequences were removed with trimmomatic^99^ using LEADING:3 TRAILING:3 SLIDINGWINDOW:4:20 MINLEN:50. Trimmed reads were then mapped to the respective genomes using bowtie2^100^ with the default parameters. The SAM output file was converted to .bam format, the reads were sorted by position and duplicates were removed using SAMtools (v.1.8)^69^. Secondary alignments (that is, multi-mapping reads) were removed using the flag -F 0x0100. The resulting .bam file was filtered using SAMtools view to retain only reads that align over their full read length. The filtered .bam files were indexed using SAMtools index with the -c flag. The ratio of ChIP/input coverage was calculated using the deeptools function bamCompare using MAPQ ≥ 30 as a threshold^98^. To define centromere boundaries, the epic2 peak caller was used to identify peaks of CENH3 enrichment with a MAPQ ≥ 30 filtering^101^. Centromere boundaries were then defined by the density of epic2 peaks with a resolution of 100 kb (Supplementary Fig. 4). To assess the contiguous assembly of the centromeres, we obtained the breakpoints (start and end) of each contig in the pseudomolecule assemblies using MUMmer (v.4.0.0.2)^102^. We then compared contig breakpoints to the CENH3 read density plots to see whether the centromeres were present on a single contig.

#### Tandem repeat annotation

Tandem repeats were identified and annotated using tandem repeats finder^103^ using the recommended standard settings but with 2,000 bp max periods size (match = 2, mismatch = 7, delta = 7, PM = 80, PI = 10, minscore = 50, maxperiod = 2000). To complement the analysis with tandem repeats finder, we also searched for instances of tandemly repeated *RLG_Cereba* elements (for example, *RLG_Cereba* elements with three long terminal repeats and two internal domains, which resulted from unequal recombination between LTRs) using BLASTN queries against the TA299 genome assembly. In this way, 61 instances of tandemly repeated *RLG_Cereba* elements were identified. As a control, we also searched for such recombinant *RLC_Angela* elements, which are around ten times more abundant than *RLG_Cereba* elements, but largely absent from functional centromeres. This revealed 620 tandemly repeated *RLC_Angela* elements. From this analysis, we conclude that, although there are tandemly repeated *RLG_Cereba* elements, the number is in the range of what could be expected from other non-centromeric TEs, revealing no higher-order structure of *RLG_Cereba* elements.

#### RNA-seq read mapping and feature counting

RNA-seq reads from the six tissues of TA299 and TA10622 were mapped to the respective genomes using STAR aligner (v.2.5.2a)^73^ with the flags --outFilterMultimapNmax 20000, --outFilterMismatchNoverLmax 0.0, --alignIntronMax 1000. Features were counted using featureCounts (v.2.0.0)^104^.

#### Genome-wide TE annotation

TE annotation was performed using EDTA with the settings --overwrite 1 --sensitive 1 --anno 1 --evaluate 1 and using the current version of TREP (v.19) as a curated input library. The identified TEs were subsequently extracted from the assemblies and BLAST searched against TREP to make family-level classifications (because, for full-length elements, EDTA is sometimes hesitant to assign a TE family and instead gives a unique identifier and superfamily tag). To determine the TE content of einkorn centromeres, centromeric DNA was annotated using the automated TE annotation software EDTA^77^ with TREP as curated input library^78^. For the analysis of genes in centromeres, all annotated genes that showed homology to TEs were removed, as it is very probable that they are TEs that were annotated as genes by mistake.

#### *RLG_Cereba* dating

Full-length copies of *RLG_Cereba* and *RLG_Quinta* retrotransposons were identified using our previously described pipeline^105^. This was done in addition to the automated TE annotation described above to extract high-quality datasets of full-length TEs. The insertion ages of full-length LTR retrotransposons were determined on the basis of the divergence of the two LTRs, which are identical at the time of insertion, and which accumulate mutations over time^106^. This produced information on insertion age and precise chromosomal location for each full-length *RLG_Cereba* retrotransposon.

#### Chromosome collinearity and similarity analysis

Collinearity of chromosomal segments was visualized using 1 kb sequence segments of one chromosome in BLASTN searches against the other chromosome. The positions of the top BLASTN hits were used for the dot plot alignments. A sliding step of 10 kb was used. The chromosome comparison was done using the original Perl script blast_compare_chromosome that is available at GitHub (https://github.com/Wicker-Lab/Monococcum_genome_scripts). To complement our homology and annotation-based repeat analysis, we used the ChIP-Seq mapper tool, which is part of the RepeatExplorer2 software collection. First, repeats were identified by clustering short sequencing reads with RepeatExplorer2, which does not depend on the reference genome and would therefore also identify repetitive elements missing from the reference. For this, around 20× coverage Illumina short reads were downsampled to the recommended coverage equivalent of 0.5× using seqkit (https://github.com/shenwei356/seqkit). CENH3 ChIP and control reads were then mapped onto the identified repeat clusters using the ChIP-Seq mapper tool. Two repeat clusters passed the threshold of greater than fivefold enrichment. The unique sequences contained in these two clusters were then queried using BLASTN searches against the nrTREP20 repeat database. In one of the clusters, all 38 sequences showed very high homology to either the *RLG_Cereba* or *RLG_Quinta* consensus sequence, whereas, in the other cluster, 37 out of 42 sequences were *RLG_Cereba* or *RLG_Quinta* sequences. Thus, ChIP-Seq mapper did not identify any additional repeat clusters enriched in CENH3 that were not found in the homology and annotation-based repeat analysis.

### Whole-genome sequencing of the einkorn diversity panel

#### Illumina short-read sequencing

We extracted genomic DNA from one or two young leaves of 219 einkorn accessions using CTAB extraction^46^. DNA quantification was performed using the Qubit dsDNA HS Assay (Q32851, Thermo Fisher Scientific), the purity was assessed using the Nanodrop spectrophotometer by checking the 260/280 and 260/230 ratios and the integrity was confirmed by analysing 1 μl per sample on a 1% TAE agarose gel. Library preparation and sequencing (150 bp paired-end libraries) were performed by Novogene using the Illumina NovaSeq 6000 system.

#### Read mapping and SNP calling

Whole-genome sequencing data for the 219 einkorn accessions were first trimmed using trimmomatic^99^ (v.0.38; parameters: LEADING:20 TRAILING:20 SLIDINGWINDOW:5:20 MINLEN:5). Reads were mapped to the TA299 reference assembly using BWA^96^ mem (v.0.7.17). The mapped reads were then sorted according to genomic coordinates using the SAMtools^69^ command sort (v.1.8). Duplicated reads were marked and read groups were assigned using the Picard tools (http://broadinstitute.github.io/picard/). HaplotypeCaller from GATK^107^ (v.4.1.8.0) was used to identify variants and generate individual-specific .gvcf files followed by a joint calling of variants performed by GenotypeGVCFs. We extracted SNPs using the GATK SelectVariants command. SNPs were hard filtered using VariantFiltration removing putative variants according to the following criteria: ‘QD < 2.0 || FS > 60.0 || MQ < 40.00 || MQRankSum < −12.5 || ReadPosRankSum < −8.0 || SOR > 3.0’. In total, 208,855,939 SNPs were called from 219 einkorn accessions. After quality control using VCFtools^108^ (v.0.1.17), the raw SNPs were filtered using GATK^107^ (v.4.1.8.0) and VCFtools^108^ (v.0.1.17) as follows: SNP clusters, defined as three or more SNPs located within 10 bp; low and high average SNP depth (4 ≤ DP ≥ 15); and SNPs located in the unanchored chromosome were removed. Moreover, one misclassified accession (TA574; initially was classified as γ) was removed on the basis of PCA and divergence analysis. Finally, only biallelic SNPs were retained for further analyses, representing a final VCF file of 121,459,674 SNPs (Supplementary Table 15). These SNPs were annotated using snpEff^109^ (v.5.0e) with TA299 HC gene models. The false-positive error rate of variant calling (percentage of polymorphic sites in a resequenced TA299 sample compared with the TA299 reference) was 0.008%, which is comparable to the error rates of other studies^43–46^ (Supplementary Fig. 19a). Variants were evenly distributed across the seven chromosomes, except for the centromeres that showed a marked reduction in variant densities due to reduced read mapping (Supplementary Fig. 19b, Supplementary Fig. 20 and Supplementary Table 16). Approximately 2.2% of the total SNPs were gene-proximal (2 kb upstream and downstream of a coding sequence). An additional 0.8% of the SNPs were located in introns and 0.5% in exons. Of the exonic SNPs, 317,023 (53.4%) were non-synonymous affecting 26,505 genes, of which 9,145 SNPs resulted in a disruption of coding sequences (premature stop codon) in 5,726 genes. Furthermore, 45.7% of the total SNPs (55,558,212 SNPs) represented rare variants with a minor allele frequency below 1% (Supplementary Fig. 19c and Supplementary Table 17). Variant calling using the TA10622 assembly revealed very similar results on the basis of population divergence, PCA and nucleotide diversity (α, *π* = 0.0012; β, *π* = 0.0017; γ, *π* = 0.0022; domesticated, *π* = 0.0012; Supplementary Fig. 21a–c), confirming the high accuracy of variant calling and the independence of population structure analyses from which reference assembly is used. The SNP calling against the TA10622 reference assembly was used for the analyses presented in Extended Data Fig. 7a,b,e.

#### Mapped reads and SNP data statistics

Mapping statistics for each accession were calculated from the BAM files using SAMtools^69^ (v.1.8; option ‘flag-stat’) to get the number of mapped reads, and the mapping rate was then calculated as follows: (the number of mapped reads/the total number of reads) × 100 . The false-positive error rate of SNP calling was calculated as the proportion of segregating sites in a resequenced TA299 sample compared with the TA299 reference assembly. The numbers of homozygous reference, heterozygous reference and alternative alleles were obtained using VCFtools^108^ (v.0.1.17) and awk command-line. The SNP density was calculated in bin sizes of 1 Mb, and the nucleotide diversity was calculated in sliding windows of 10,000 bp per chromosome and then averaged across the entire genome to measure the degree of polymorphism within each einkorn population using VCFtools^108^ (v.0.1.17).

#### Population diversity and structure

We assessed the genetic relationships between accessions with PCA using all SNPs (121,459,674) with PLINK^110^ (v.1.90). The unrooted neighbour-joining phylogenetic tree was generated from filtered SNPs (missing data > 10% and 5% randomly sampled SNPs; total SNPs = 5,318,268). First, the genetic distances were computed using Euclidean distances with the ‘dist’ function in the stats R package. The distance matrix was converted to a phylo object using the R package ape and the tree was generated using the phyclus R package. For estimating individual ancestry coefficients, the R package LEA ‘snmf’ function was used with the entropy option and with 10 independent runs for each *K* (*K* is the number of putative ancestral populations) from *K* = 1 to *K* = 10 using the same SNP subset used to generate the phylogenetic tree. The cross-entropy value decreased with increasing *K* and reached a plateau starting from *K* = 6 (Supplementary Fig. 14).

#### *F*_ST_ calculation

We defined the two domesticated einkorn groups on the basis of an ancestry threshold of 80% at *K* = 4 (because the split of the two domesticated einkorn groups occurred at *K* = 4). We then calculated the mean fixation index (*F*_ST_) between these two domesticated einkorn groups in 1 Mb non-overlapping genomic windows using VCFtools^108^ (v.0.1.17).

#### Wild einkorn γ race introgression analyses

We evaluated the divergence of domesticated einkorn accessions from the TA10622 reference assembly by calculating the proportion of segregating sites using only the diverged blocks (chromosome 2A: 261–406 Mb; chromosome 5A: 92–409 Mb; and chromosome 7A: 301–448 Mb). Moreover, we performed PCA using all einkorn accessions with only SNPs located in the diverged regions. To estimate the proportion of γ race introgression into all domesticated einkorn accessions, we calculated pairwise nucleotide diversity in 1 Mb non-overlapping windows with VCFtools^108^ (v.0.1.17) between one γ accession (TA10600) and each of the domesticated einkorn accessions. Accession TA10600 was selected because it showed a low divergence from the introgressed segments. A region was considered as introgression if (1) there was a continuous nucleotide diversity reduction for ≥10 Mb; (2) nucleotide diversity reduction in the region was not observed between an α race accession and a domesticated accession; and (3) the reduction of nucleotide diversity was not due to the lack of mapped reads. To identify γ accessions with the closest genetic relatedness to the introgressed segments in domesticated einkorn, we first performed a PCA using only wild γ race and domesticated einkorn accessions with the introgressed genomic regions (chromosome 2A: 261–406 Mb; chromosome 5A: 92–409 Mb; and chromosome 7A: 301–448 Mb, separately). On the basis of the clustering of accessions in the PCA, the geographical projection of the first (for the regions on chromosome 2A and 7A) and the second PCA axes (for the region on chromosome 5A) was done and visualized using the Kriging function in the fields v.10.3 R package (https://cran.r-project.org/web/packages/fields).

#### TreeMix analysis

We included *T. urartu* whole-genome sequencing data^44^ in the analysis, and SNP calling was performed as described above. We filtered all missing SNPs, and we used PLINK^110^ (v.1.90) to remove SNPs in linkage disequilibrium (parameter: indep-pairwise 100 5 0.2). The total number of SNPs retained for the TreeMix analysis was 1,042,531. To infer splits and admixture events, we first obtained a list of einkorn groups considering 80% ancestry threshold at *K* = 6. We then used TreeMix (v.1.13)^49^ using jackknife blocks of 1,000 SNPs and modelling 5 migration events.

### *k*-mer based approaches to detect einkorn introgressions in bread wheat

We used two *k-*mer-based approaches to identify putative einkorn introgressions into bread wheat.

#### *k*-mer mapping approach

For generating *k*-mer datasets, we used the whole-genome sequencing data from all domesticated einkorn accessions in the panel and *T. urartu* accessions^44^. *k*-mers (*k* = 51) were counted from the Illumina raw data per accession using jellyfish (v.2.2.10)^111^. We extracted the *k*-mer nucleotide sequences from each accession of *T. monococcum* and *T. urartu*. We concatenated all *k*-mer sequences from all *T. monococcum* accessions and *T. urartu* accessions into one separate file for each species and retained one representative per *k*-mer. We removed common *k*-mers between *T. monococcum* and *T. urartu* and obtained a list of specific einkorn and *T. urartu k*-mer sequences, respectively. The lists of specific *k*-mers were later converted into fasta files. Each fasta file (*T. monococcum* and *T. urartu*) was mapped against the bread wheat genomes^23^ using BWA^96^ mem (v.0.7.17), requiring mapping of only full-length *k*-mers with no mismatches. Mapped *k*-mers in each .bam file (*T. monococcum* and *T. urartu*) were analysed for the coverage in genomic windows of 1 Mb using mosdepth^112^ and visualized in R (v.4.0.4) using ggplot2. Putative introgressions were identified as an increased coverage of mapped *k*-mers from *T. monococcum* (with an average coverage of ≥5), but depleted mapping of *T. urartu*-specific *k*-mers. Two or more regions were grouped into one if they were no more than 1 Mb apart.

#### *k*-mer variation approach

We implemented Identity-by-State Python (IBSpy; https://github.com/Uauy-Lab/IBSpy), a *k-*mer-based pipeline that counts variations in 50 kb windows, and used it to detect *T. monococcum* introgressions into the ten bread wheat genomes^23^ (Supplementary Note 2). We first used KMC3^113^ to build a *k-*mer (*k* = 31) databases from the Illumina raw data of 218 *T. monococcum* accessions, the two *T. monococcum* chromosome-scale assemblies and ten genome assemblies of wheat. We next compared the *k-*mers of the bread wheat reference sequence to the *k-*mers of each database and counted the number of variations within each 50 kb window. We used variations ≤ 30 as a cut-off (Supplementary Fig. 22 and Supplementary Note 2). We considered the six STRUCTURE groups to identify the putative donors for each introgressed segment (Fig. 3b, Supplementary Fig. 13 and Supplementary Note 2).

### Positional cloning of *tin3*

#### Phenotyping and DNA isolation of tin3 mutants

A cross between the *tin3* mutant and the parental accession TA4342-L96 was made. A total of 375 F_2_ plants were grown and phenotyped for tiller number (Zadoks growth scale ~Z29). Leaf tissues were harvested and DNA was extracted using the BioSprint 96 DNA Plant Kit (Qiagen, 576) on a KingFisher Flex robot (Thermo Fisher Scientific, 5400610) according to the manufacturer’s instructions. Equimolar concentrations of 30 F_2_ plants showing the *tin3* mutant phenotype were pooled to create a mutant bulk. SEM samples were fixed in 3% glutaraldehyde, post-fixed in 1% osmium tetroxide and dehydrated in a graded acetone series, and these samples were processed and SEM images were captured as described previously^114,115^.

#### Whole-genome sequencing, alignment and SNP calling

Sequencing libraries were prepared, and sequencing was performed by Novogene using the Illumina NovaSeq 6000 platform. Samples were sequenced to >13-fold coverage. We used the Illumina reads of TA4342-L96 (Sequence Read Archive: SRR21543761) as the parental control. We followed the MutMap protocol with minor modifications^57^. High-quality filtered reads were aligned to the *T. monococcum* accession TA10622 using BWA^96^. SAM files were converted into .bam files using SAMtools^69^. SAMtools (markdup option) was used to mark and remove PCR duplicates. Improperly mapped read pairs were removed from the .bam files retaining only concordantly aligned reads with MAPQ ≥ 30. The BCFtools mpileup tool was used for SNP calling^70^. SNPs were filtered on the basis of the following criteria: minQ ≥ 30, Fisher Strand (FS) > 40, mapping quality (MQ < 40), minDP > 3 and genotype quality (GQ < 20). SNPs within 10 bp proximity of indels were removed and only the biallelic SNPs were retained. SNP positions with an identical allele in both TA4342-L96 and the *tin3* mutant bulk were treated as varietal SNPs and were removed from the analysis. SnpSift^109^ was used to select EMS-type (G/C to A/T) transitions from the VCF file. We considered the positions with a SNP index of ≥0.9 to be homozygous, whereas SNPs with an SNP index of <0.3 were removed, and the rest were considered to be heterozygous. We used the mutplot tool (https://github.com/VivianBailey/Mutplot) to calculate the average SNP index using a window size of 100 kb^116^. The average SNP index was plotted along the chromosomes using ggplot2^117^. SnpEff 5.0c (build 2020-11-25 14:23) was used to calculate the effect of the variants on genes.

#### KASP assay

A KASP marker was designed on the basis of the SNP in *Tm.TA10622.r1.3AG0164370* using Primer3 software (primer F1, 5′-**GAAGGTGACCAAGTTCATGC**TCGCCGTCCTCACCCAGa-3′; primer F2, 5′-**GAAGGTCGGAGTCAACGGAT**TCGCCGTCCTCACCCAGg-3′-; primer R, 5′-ATCCCAACATAACCGACCCC-3′; the HEX and FAM tails are marked in bold, and the lower case base indicates the allele-specific SNP). KASP assays were performed using KASP-TF V4.0 2× Master Mix (LGC Bioscience Technologies, KBS-1050-102) with a 5 μl reaction in the Bio-Rad C1000 thermal cycler with a CFX96 module according to the manufacturer’s instructions. Allelic discrimination was called using CFX Maestro Software (v.1.1).

#### TILLING in hexaploid wheat cv. ‘Jagger’

The TILLING population used in this study was first reported previously^59^. Markers were designed for the A, B and D subgenome homeologous copies of *tin3* (*tin3A*, *tin3B*, *tin3D*) using GSP^118^ and tested for specificity using nullisomic tetrasomic wheat lines. PCR products were amplified for each target gene on 4× pools. These products were heteroduplexed to form base pair mismatches and digested using homemade Cel-1 endonuclease. Individual target genes were then amplified from positive pools. Heterozygous mutants, such as in *tin3D*, were grown for an additional generation to retrieve homozygous mutants. Zygosity was confirmed by isolating DNA from the segregating plants and Sanger sequencing. Crosses between the different mutants were made and plants identified as homozygous for single mutations (that is, *tin3A*, *tin3B*, *tin3D*), double mutations (*tin3AB*, *tin3AD*, *tin3BD*) and triple mutations (*tin3ABD*) were selected. Plants were phenotyped for tiller numbers 8 weeks after vernalization.

### Germplasm availability

The *T. monococcum* accessions used in this study are listed in Supplementary Table 8 and are available on request from the WGRC, Kansas State University (www.k-state.edu/wgrc).

### Reporting summary

Further information on research design is available in the Nature Portfolio Reporting Summary linked to this article.

## Online content

Any methods, additional references, Nature Portfolio reporting summaries, source data, extended data, supplementary information, acknowledgements, peer review information; details of author contributions and competing interests; and statements of data and code availability are available at 10.1038/s41586-023-06389-7.

### Supplementary information

Supplementary Information Supplementary Notes 1 and 2, Supplementary Figs. 1–22, Supplementary Tables 1, 5, 6, 14 and 15, and Supplementary References 119–132.

Reporting Summary

Peer Review File

Supplementary Tables Supplementary Tables 2–4, 7–13 16 and 17.

### Source data

Source Data Extended Data Fig. 9

## Figures and Tables

**Extended Data Table 1:**
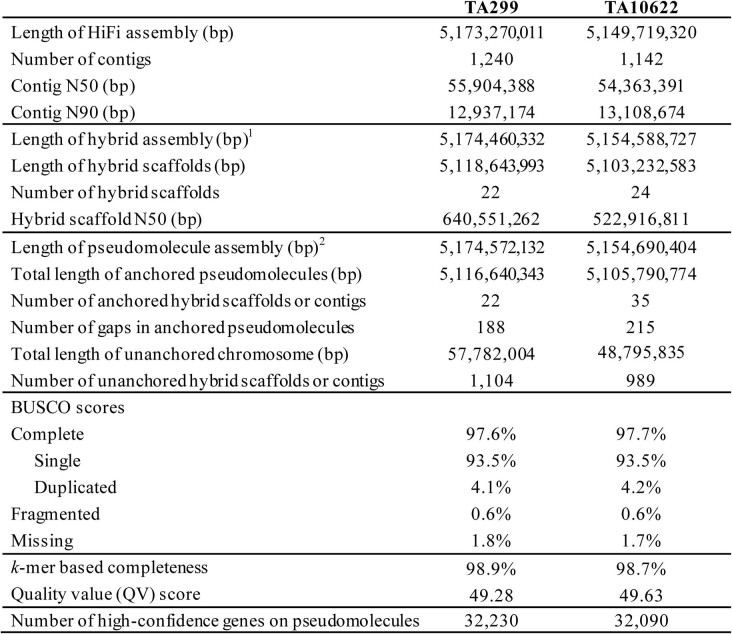
Summary of einkorn assemblies and annotations

^1^Hybrid scaffolds were created by integrating optical maps into the contig-level assembly. The hybrid assembly includes all hybrid scaffolds and contigs that were not integrated into hybrid scaffolds.

^2^Includes contig-level assembly, optical map, and Omni-C data.

## Data Availability

The raw sequencing data used for de novo genome assemblies, the RNA-seq and Iso-seq data for the annotation, the ChIP–seq reads and the whole-genome sequencing reads of 218 einkorn accessions are available at the EBI-ENA under study number PRJEB61155. The two reference assemblies, annotations, VCF files and CpG methylation frequencies are available at DRYAD (10.5061/dryad.v41ns1rxj). Raw fastq files and demultiplexed fastq files of the RIL population have been deposited at the National Center for Biotechnology Information (NCBI) SRA database under BioProject accession PRJNA879879. The barcode indices key file with required information for demultiplexing can be obtained at DRYAD (10.5061/dryad.v41ns1rxj). CENH3 BED files (CENH3 peaks) and mapped files (.bam) of CENH3 and H3K4me3 for all replicates are available through the Dryad database (10.5061/dryad.0p2ngf24b). Whole-genome sequencing data of the *tin3*-mutant bulk have been deposited at GenBank under BioProject PRJNA938447. IBSpy variations tables of the 218 einkorn accessions, and MUMer alignments of the two einkorn assemblies against the ten bread wheat assemblies are available online (https://opendata.earlham.ac.uk/wheat/under_license/toronto/Uauy_2022-09-24_IBSpy_Triticum_monococcum_introgressions/). An interactive webpage has been developed for this study to visualize various genome characteristics of TA299 and TA10622. The webpage features a JBrowse 2 explorer enabling visualization of the whole genome, gene models, transposable elements, variants positions and synteny. For the identification of homologous sequences in other wheat varieties, a BLAST server has been set up. This BLAST server enables searches against individual wheat subgenomes and chromosomes independently. Synteny between TA10622 and other wheat genomes can be visualized in the synteny tab located on the webpage. The database can be accessed online (https://wheat.pw.usda.gov/GG3/pangenome). Source data are provided with this paper.

## Code availability

Custom scripts for variant calling are available at GitHub (https://github.com/IBEXCluster/Wheat-SNPCaller). The necessary codes and steps to genotype the RIL population were published previously^67^ and are available in a separate file on the DRYAD database (https://datadryad.org/stash/share/v20dkVsSTj3toGn-CHG92eUSgre17uMT5AH_6LE2GDM). Codes and custom scripts for analysing einkorn introgressions into bread wheat are available at GitHub (https://github.com/Uauy-Lab/monococcum_introgressions).
