# Supplementary material for: Einkorn genomics sheds light on history of the oldest domesticated wheat
Source: Nature. 2023 Aug 2;620(7975):830–8. doi: 10.1038/s41586-023-06389-7 (PMC10447253; doi:10.1038/s41586-023-06389-7)
Supplement: Supplementary file 1 — Supplementary Notes 1 and 2, Supplementary Figs. 1–22, Supplementary Tables 1, 5, 6, 14 and 15, and Supplementary References 119–132. [file 41586_2023_6389_MOESM1_ESM.pdf]

nature portfolio
https://doi.org/10.1038/s41586-023-06389-7

Supplementary information

Einkorn genomics sheds light on history of the oldest domesticated wheat

In the format provided by the authors and unedited

Table of Contents

Supplementary Note 1 - Einkorn does not contain major non-TE derived centromeric tandem repeats.
Supplementary Note 2 - Implementation of IBSpy to identify *T. monococcum* introgressions in bread wheat.
Supplementary Figures 1 - 22
Supplementary Table 1 - Statistics of the Bionano optical maps and hybrid assemblies.
Supplementary Table 5 - Number of transcripts expressed in the different tissues considering only high-confidence gene models on the seven pseudomolecules. The estimation of expression was done based on TPM (Transcripts Per Million) using the RSEM pipeline.
Supplementary Table 6 - Boundaries of functional centromeres as defined by CENH3 sequence read coverage in chromosome assemblies of *T. monococcum* accessions TA299 and TA10622.
Supplementary Table 14 - Corrected genomic regions in both TA299 and TA10622 genome assemblies based on the genetic linkage maps.
Supplementary Table 15 - Number of SNPs retained after each filtering step.

Supplementary Note 1

Einkorn does not contain major non-TE derived centromeric tandem repeats

To identify tandem repeats (TRs) occurring in the centromeres of einkorn, tandem repeats finder[119] was run on the centromeric sequences of each chromosome. Centromeric tandem repeats in most sequenced plant genomes have a size of ~150-180 bp[120-124], giving them the approximate length of a single wrap around a nucleosome. In hexaploid wheat however, tandem repeats of up to 566 bp were reported in centromeres[125]. We therefore filtered the resulting tandem repeats for minimal number of 3 repeats in tandem, minimal period length of 50 bp, maximal period length of 600 bp and a minimal array size of 500 bp. The filtered 56 (TA10622) and 81 (TA299) TRs were used in BLASTN searches against the TREP database (https://www.botinst.uzh.ch/en/research/genetics/thomasWicker/trep-db.html). This showed that 47 (TA10622) and 67 (TA299) of the filtered TRs are either part of a transposable element or arose from partial sequences of transposable elements. This is in line with previous findings in wheat, reporting the presence of TRs inside *RLG_Cereba* retrotransposons[126]. Such TR arrays may arise spontaneously through slipped strand mispairing[127] (for short repeats) and, for longer repeats, through unequal recombination or unequal synthesis-dependent strand annealing[128]. TR arrays have long been known to be frequent also in other, non-centromeric TEs[129]. The TRs that were not clearly part of TEs were used in BLASTN searches against all the centromeres of the respective genome, showing that many of them occur in multiple centromeres, but none of them in more than 5 of the 7 centromeres. Furthermore, the TRs were used in BLASTN searches against the entire genome of origin, which showed that all of them are also found abundantly in chromosome arms and not predominantly in the centromeres. We thus conclude that these TRs are not counterparts of the ~150-180bp centromeric TRs reported in other plant species. Additionally, we specifically searched for the previously reported centromere-specific wheat TRs CENT566 and CENT550[125] satellite repeats. We found several dozen loci containing tandem arrays of these two repeats. However, practically all were found outside centromeres and most contained less than 10 repeat units. Thus, our data do not indicate that CENT566 and CENT550 are major centromere-specific repeats in einkorn. The only TRs we found in some abundance in centromeres were simple sequence repeats such as AAC, which are not specific to centromeres.

Despite the low fraction of TRs in centromeres, we cannot exclude the possibility that some could nevertheless contribute to centromere function. However, we did not find any association between those TRs and high CENH3 ChIP-seq read coverage.

Supplementary Note 2

Implementation of IBSpy to identify *T. monococcum* introgressions in bread wheat

We implemented the Identity-by-State python (IBSpy: https://github.com/Uauy-Lab/IBSpy) pipeline and used it to identify *T. monococcum* introgressions in ten hexaploid wheat genome assemblies representing a pan-genome of wheat cultivars. Briefly, we used KMC3[130] to build a *k*-mer database from multiple genotypes, including the Illumina raw data of 218 *T. monococcum* accessions, two *T. monococcum* chromosome-scale assemblies, and ten genome assemblies of wheat[131]. Using 50 kb windows, we compared the *k*-mers in the reference sequence to the *k*-mers of each query database and counted the number of variations within each window. A variation is defined as a set of continuous overlapping *k*-mers (k = 31; see GitHub page for more details https://github.com/Uauy-Lab/monococcum_introgressions) from the reference completely absent in the query. Low variation counts indicate high similarity between the 50 kb reference window in the assembly and the query accession (hence can be used to identify introgressions), whereas high variation counts indicate lower sequence similarities.

To define the variation cut-off for *T. monococcum* introgressions into the hexaploid pan-genome cultivars, we compared the output of sequence alignments between fully assembled references to the IBSpy variations data. We compared the published[132] pairwise MUMmer alignments among the pan-genome cultivars (Arina*LrFor*, Chinese Spring, Jagger, Julius, LongReach Lancer, CDC Landmark, Mace, Norin 61, CDC Stanley, SY Mattis) with the corresponding variations counts from IBSpy to compare the sequence identity with the variations count. In total, there were 110 pairwise alignments analyzed, and we focused on the seven A subgenome chromosomes.

We analyzed the data in 500 kb windows (a total of 890,793 windows) and kept those windows with at least a 60% breadth of alignment in the MUMmer output (77.8%; 693,102 500-kb windows). For each 500 kb window, we had the average sequence identity between the pan-genome reference and the other nine pan-genome query samples (if over 60% breadth of alignment), alongside the IBSpy variations for the equivalent comparisons using the pan-genome reference assembly and the *k*-mer database. We grouped the data based on the number of variations (in increments of 10 variations per bin) and determined the distribution of the sequence identity in each bin (Supplementary Fig. 22). We identified that most of the 500 kb windows (532,248; 76.8%) had 30 or less variations per 50 kb. On average, these windows with 30 or less variations determined by the IBSpy method had sequence identity of 99.95% when the corresponding full genome assemblies were compared for the respective region. The data distribution was such that a 500 kb window with ≤30 IBSpy variations has a 0.926 probability that the alignment of this window will have at least 99.9% sequence identity; and a 0.997 probability that the sequence identity will be at least 99.8%.

We performed a similar analysis by generating pairwise MUMmer (v4.0.0.2) (parameters: --mum --delta and deta-filter -l 20000 for filtering; i.e., retain only alignments ≥ 20 kbp in length) alignments per chromosome between the assemblies from the ten pan-genome cultivars[13] and the two *T. monococcum* assemblies generated here (TA299 and TA10622). As expected, across the two pairwise alignments, few 500 kb windows had alignments which covered at least 60% of the window (n= 238 windows; 0.3% of a total of 197,954 windows). Using the ≤30 cut-off, we identified 201 windows that had on average 99.91% sequence identity; and a 0.970 probability that the sequence identity between pairwise alignments will be at least 99.8%. As such, we consider pairwise comparisons with IBSpy values of ≤30 variations per 50 kb as being identical or near-identical by state, both in hexaploid wheat and between hexaploid and *T. monococcum* comparisons.

Next, we used the threshold of variation count ≤30 to identify continuous windows that belong to an introgression segment across the A subgenome of the ten wheat genome assemblies. For each 50 kb window, we determined the minimum number of variations in the raw data of the 218 accessions and the two assemblies of *T. monococcum*. Based on this "einkorn minimum" value, we identified 50 kb windows in which this value was equal to or lower than the 30 variations cut-off. These windows were considered as *T. monococcum* introgressions into the corresponding reference sequence. We next called introgressions segments (Supplementary Table 12) by stitching together 50 kb windows with variations ≤30 that were separated by less than ten non-introgression windows (i.e., with variations >30). This was done as the "non-introgression" windows often had values just above the 30 variations cut-off.

For each introgression segment, we determined the numbers and lists of *T. monococcum* accessions that contributed to the introgression based on the groups defined at *K*=6 by STRUCTURE analysis (Supplementary Table 9). An accession was assigned as contributing to an introgression segment if it had at least 20% of the 50 kb windows within the segment with variations values ≤30. For example, if an introgression segment has 60 windows, an accession would be classified as contributing to the introgression if 12 or more 50 kb windows had variation values of 30 or less. We further obtained the number of introgression segments that could be assigned to a particular einkorn group based on the top accession contributing the most to the introgression segment (Supplementary Table 12).

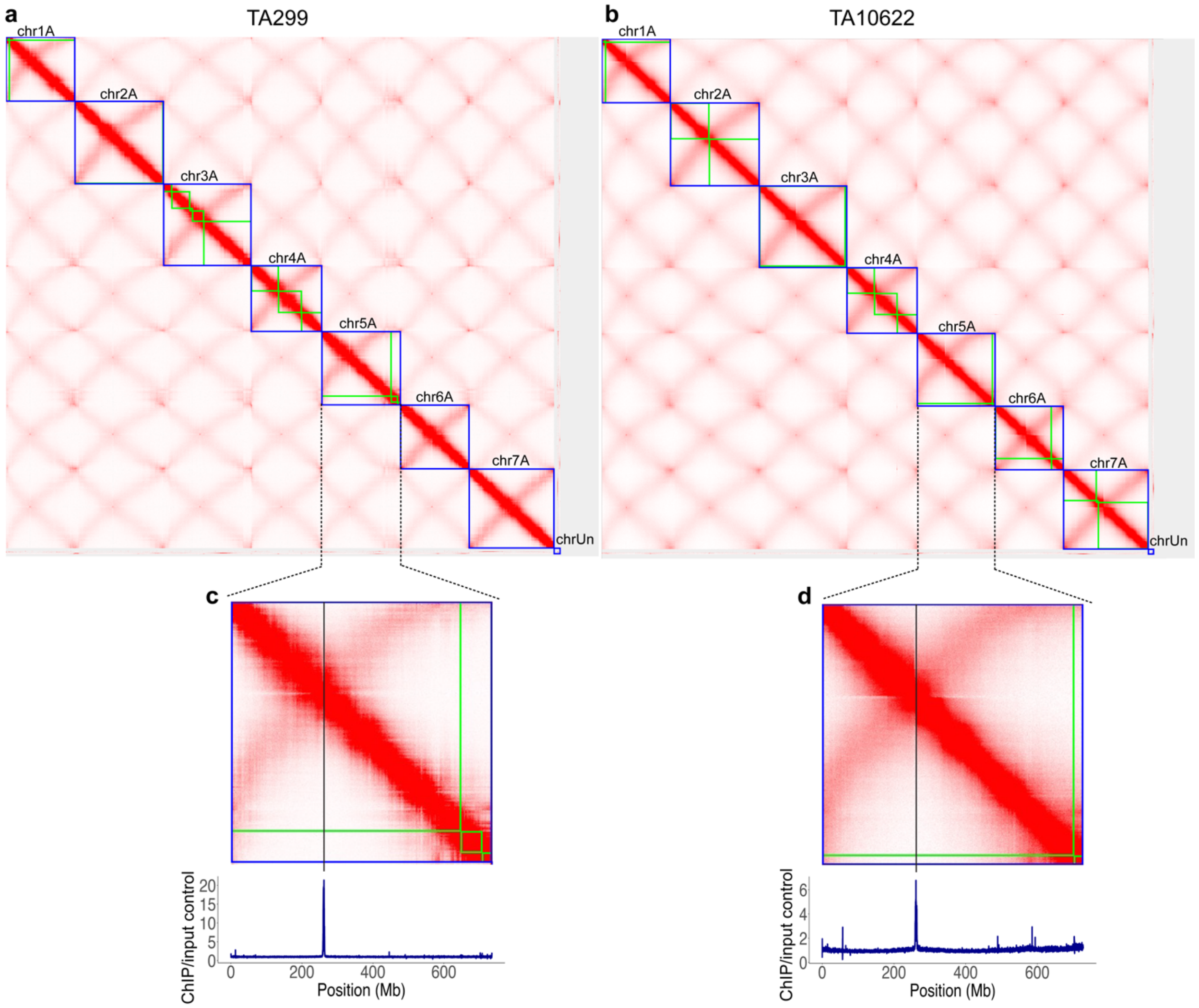

Supplementary Fig. 1. **Chromosome contact maps across the seven pseudomolecules of TA299 (a) and TA10622 (b).** The anti-diagonal reflects the Rabl configuration of wheat chromosomes, where individual chromosomes fold back, resulting in juxtaposed long and short arms. Green boxes represent individual hybrid scaffolds. Blue boxes indicate chromosomes. **c, d,** Enlarged plots of chromosomes 5A of TA299 (**c**) and TA10622 (**d**). Shown below the contact plots are the CENH3 read counts along the chromosomes. CENH3 peaks (functional centromeres) overlap with the intersection of the diagonal and anti-diagonal (black vertical lines).

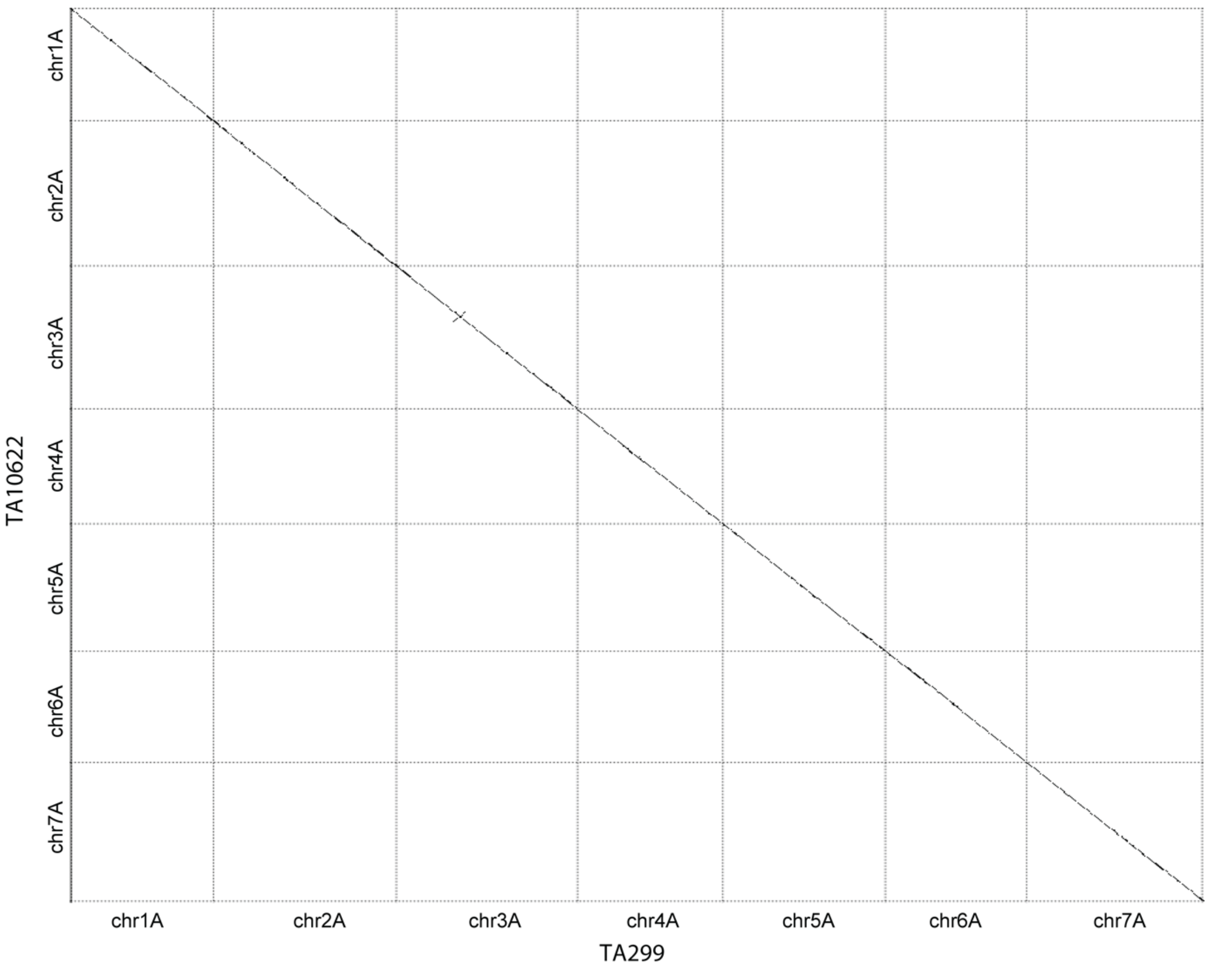

Supplementary Fig. 2. Dot plot comparison between the TA299 and TA10622 reference assemblies.

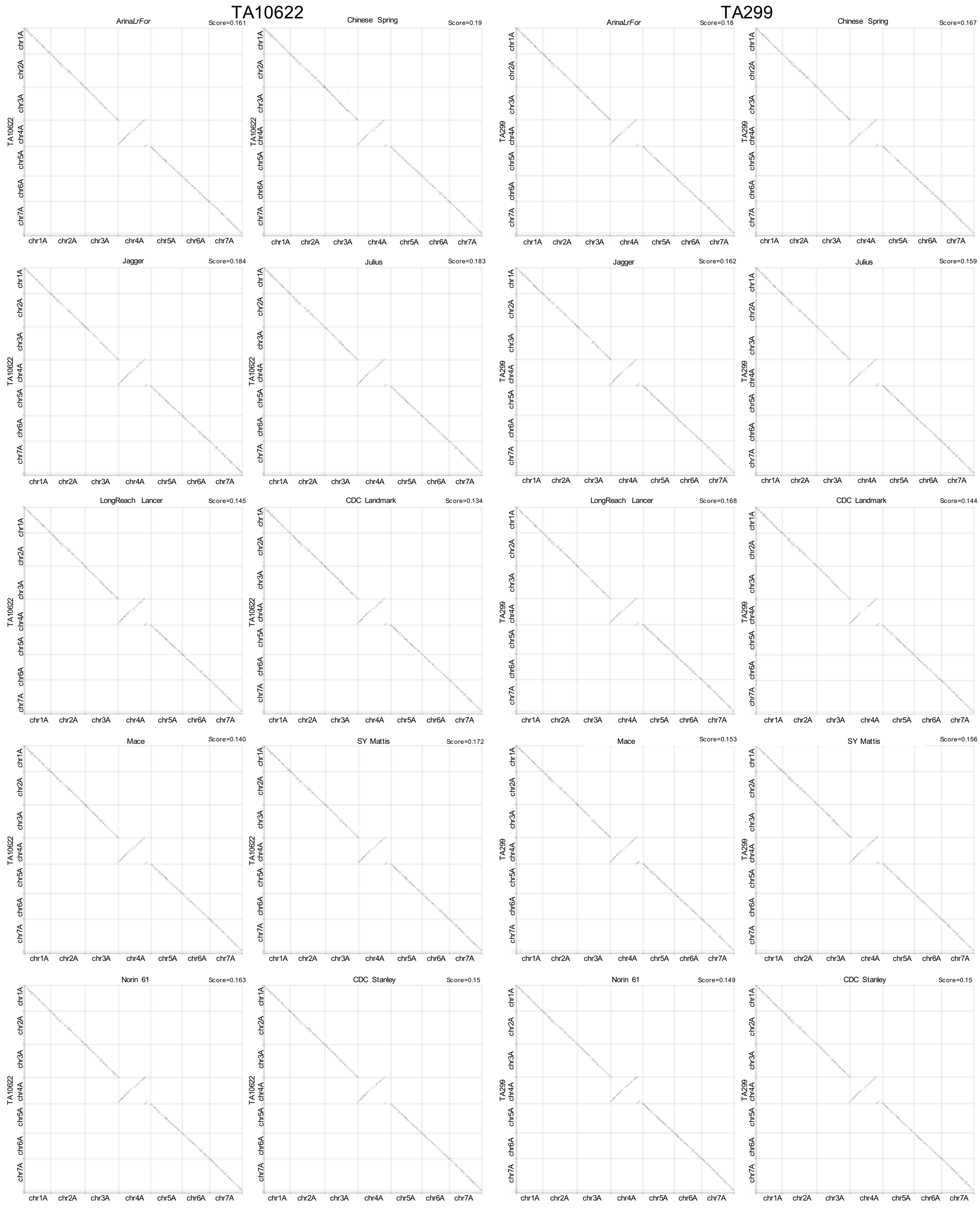

Supplementary Fig. 3. Dot plot comparisons between the two einkorn reference assemblies and ten chromosome-scale bread wheat reference genomes.

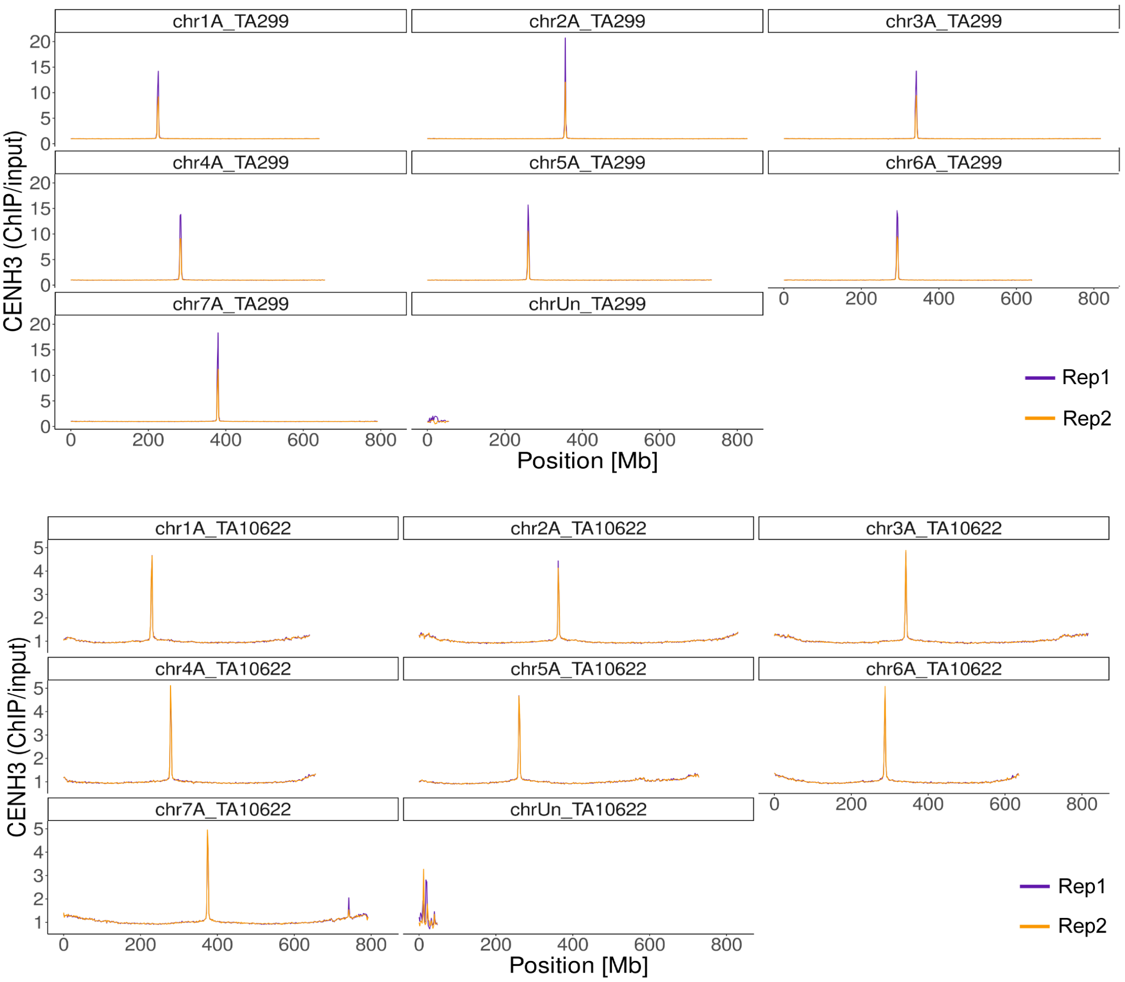

Supplementary Fig. 4. CENH3 ChIP-Seq read coverage divided by input read coverage across all chromosomes TA299 (upper three rows) and TA10622 (lower three rows). CENH3 ChIP-Seq read coverage normalized by input control across all chromosomes including unplaced contigs (chrUn). ChIP/input ratio was calculated using bamCompare in 2 Mb bins. ChIP-Seq and controls were performed in duplicates. Replicate 1 is shown in purple and replicate 2 is depicted in orange color. In the unplaced contigs of TA299 there is little coverage of CENH3 reads. The CENH3 peaks in the unplaced contigs of TA10622 correspond to the gaps in the centromere assembly of chromosome 2A. In the genome assembly of TA299, one *RLG_Cereba* element is located in the unplaced contigs, while there are 17 *RLG_Cereba* and 7 *RLG_Quinta* elements situated in the unplaced contigs of accession TA10622.

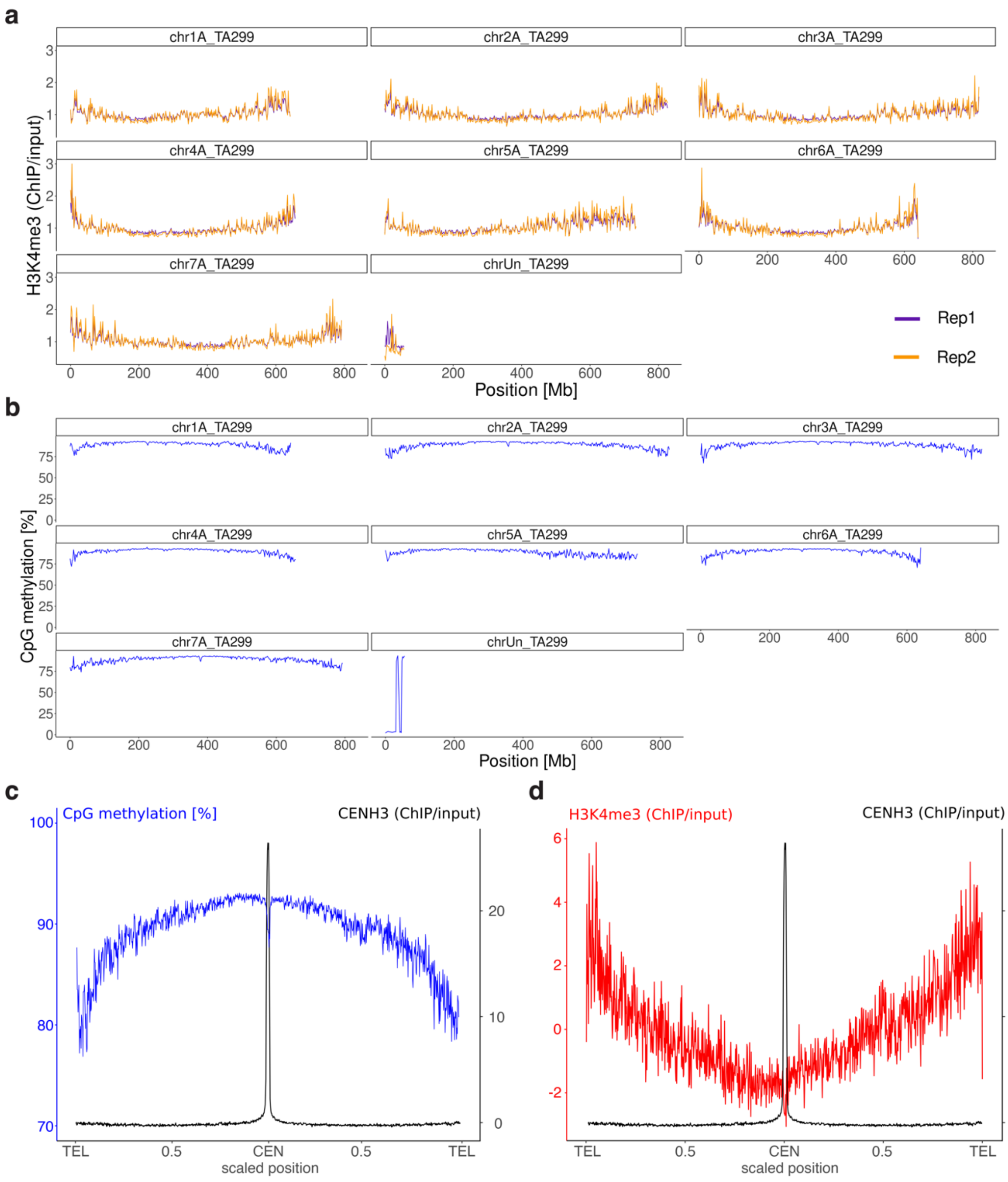

Supplementary Fig. 5. Analysis of epigenetic modifications across einkorn chromosomes. a, H3K4me3 ChIP-Seq read coverage divided by input read coverage across all chromosomes of TA299. H3K4me3 ChIP-Seq read coverage normalized by input control across all chromosomes including unplaced contigs (chrUn). ChIP/input ratio was calculated using bamCompare in 2 Mb bins. ChIP-Seq and controls were performed in duplicates. Replicate 1 is shown in purple and replicate 2 is depicted in orange color. H3K4me3 is highest in the distal parts of the chromosomes and is lowest in the centromeric and pericentromeric regions. **b,** CpG methylation across all chromosomes of TA299. CpG methylation was obtained from PacBio CCS reads using the ccsmeth package. The CpG methylation percentage per site was then averaged in 2 Mb bins, after removing sites with coverage <10. CpG methylation decreases towards the distal parts of the chromosome arms and increases towards the centromere, in the centromere there is a decrease in CpG methylation. **c-d,** CENH3, H3K4me3 and CpG methylation scaled and averaged across all chromosomes. For this, the chromosome arms of all chromosomes were separated into 500 bins of varying sizes (depending on the chromosome arm length).

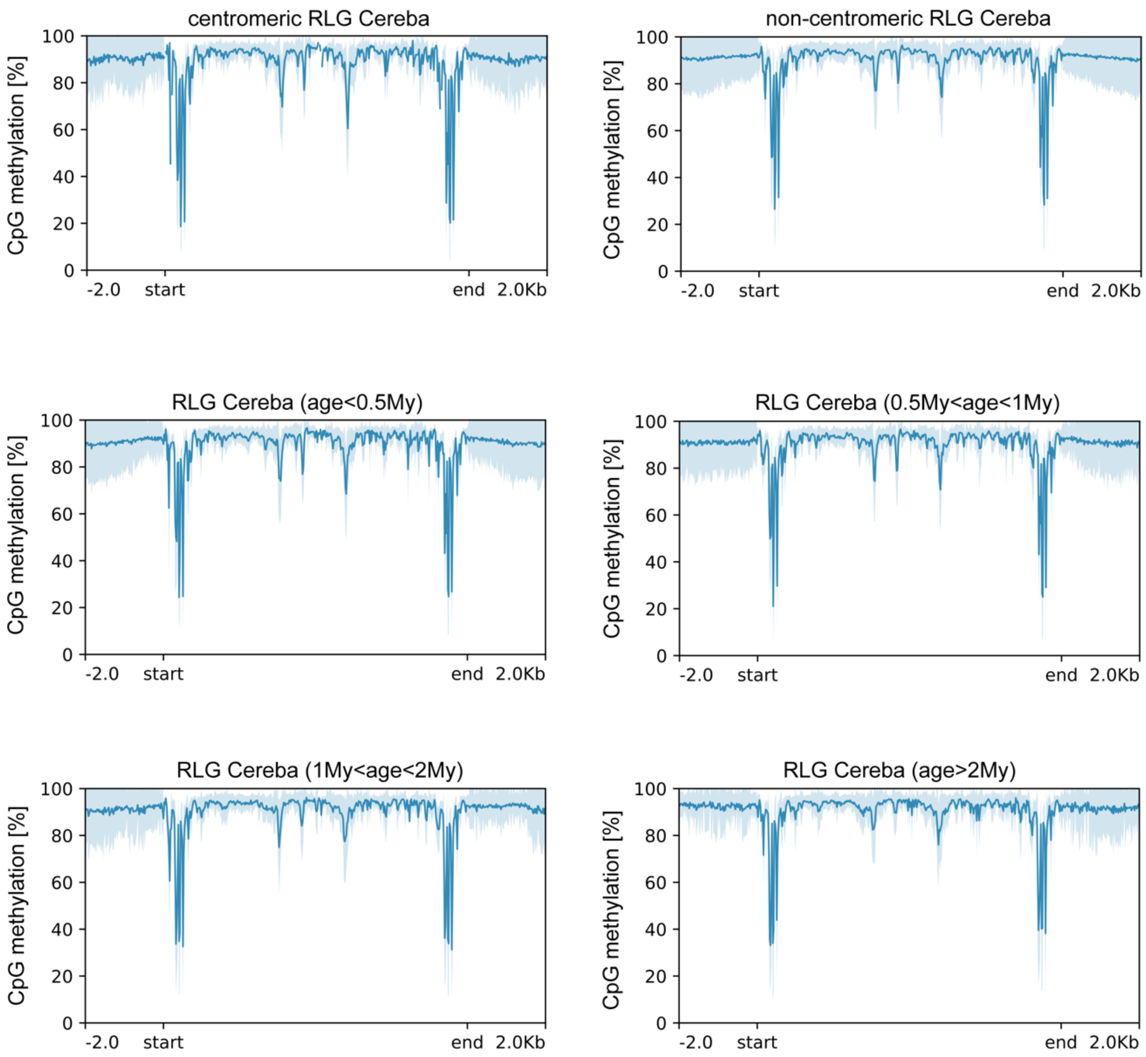

Supplementary Fig. 6. Metaplots of CpG methylation across *RLG_Cereba* elements. *RLG_Cereba* long terminal repeats contain a region of low CpG methylation. *RLG_Cereba* elements were separated into centromeric and non-centromeric localization. We did not detect a difference in CpG methylation levels, when comparing centromeric to non-centromeric *RLG_Cereba* elements, or old with young *RLG_Cereba* copies. Shaded regions indicate ±SD at each interval position.

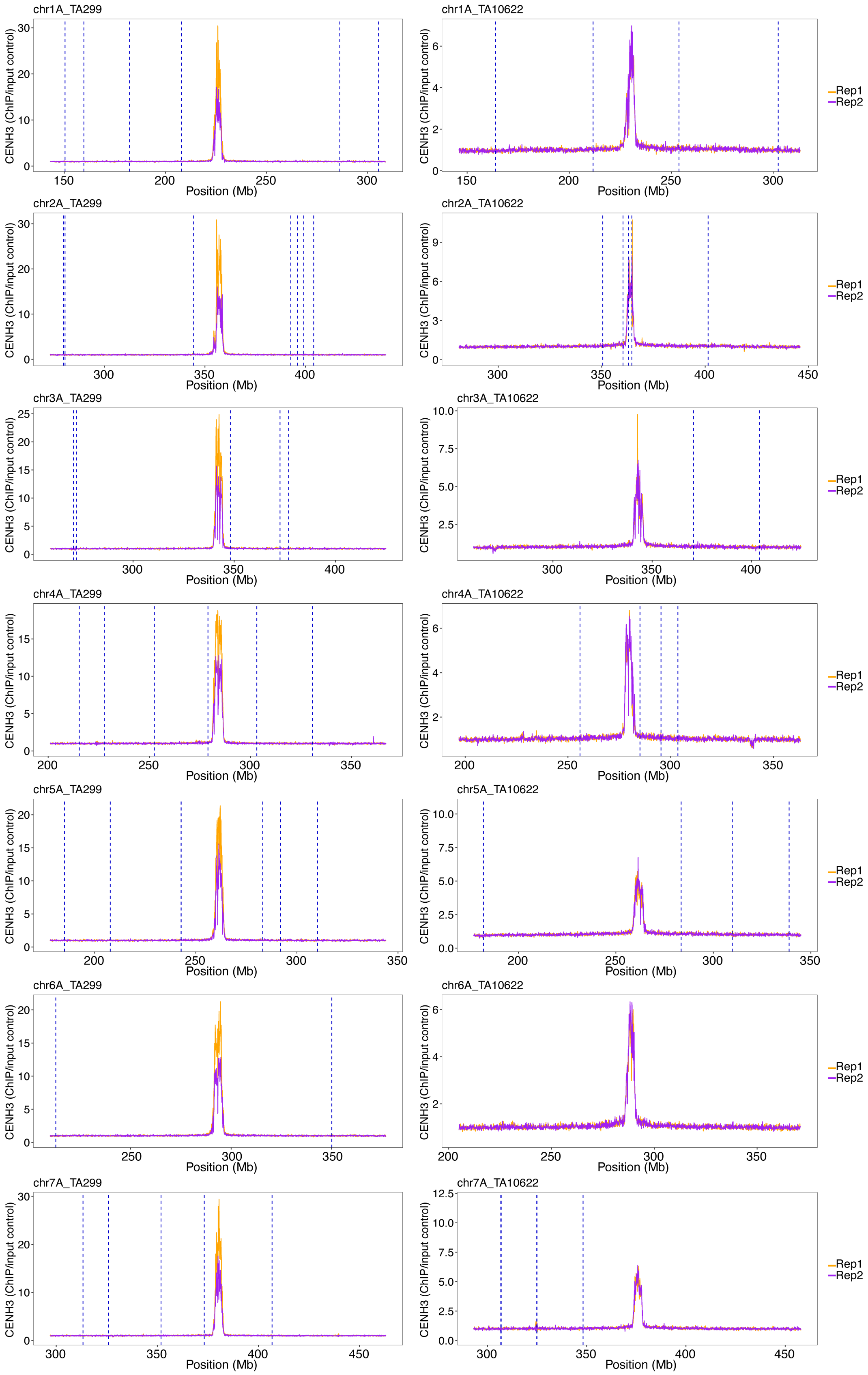

Supplementary Fig. 7. Centromeric regions of TA299 (left column) and TA10622 (right column). The CENH3 ChIP-Seq read depth (calculated in 100 kb windows) are enriched at the centromeric region (the peak in the middle). Purple and orange indicate the two replicates. Blue dashed vertical lines represent the boundaries between individual contigs.

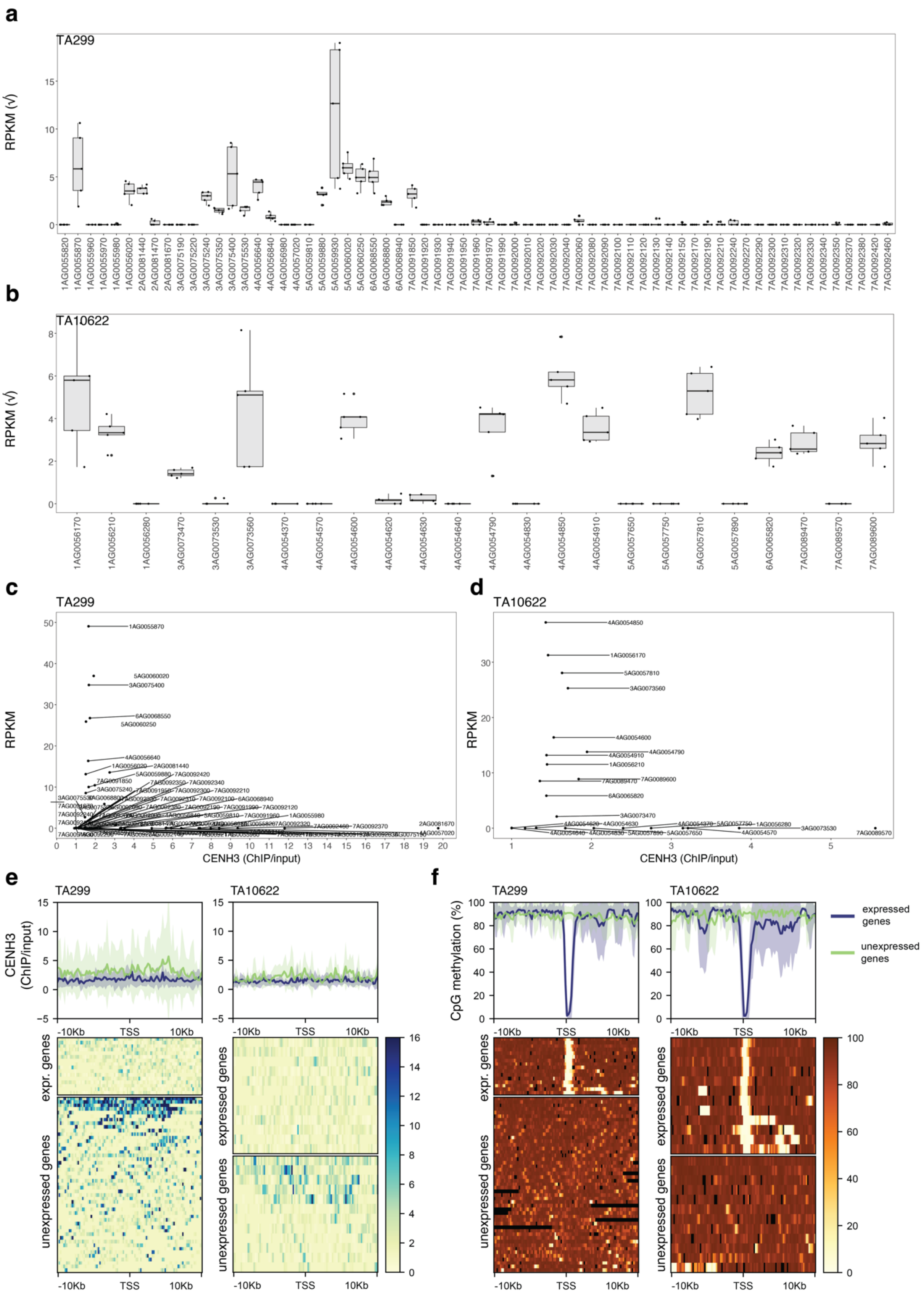

Supplementary Fig. 8. Analysis of expression, CENH3 enrichment of genes and DNA methylation in *T. monococcum* centromeres. a, Expression levels of genes found in functional centromeres of *T. monococcum* accession TA299. Expression data were obtained from five different tissues (n=5 biologically independent plant tissues). RPKM: reads per kb coding sequence (CDS) per million reads.

To better display the wide range of expression levels, the square root of RPKM values is shown. Boxes indicate the inter-quartile range (IQR) with the central line indicating the median and the whiskers indicating the minimum and maximum values without outliers extending beyond - 1.5*IQR and maximum + 1.5*IQR. **b,** Expression levels of genes found in functional centromeres of *T. monococcum* accession TA10622. Expression data were obtained from five different tissues (n=5 biologically independent plant tissues). Boxes indicate the inter-quartile range (IQR) with the central line indicating the median and the whiskers indicating the minimum and maximum values without outliers extending beyond - 1.5*IQR and maximum + 1.5*IQR **c-d,** Average ratio of CENH3 reads in ChIP divided by input control of genes found in functional centromeres plotted against their median gene expression levels for TA299 (**c**) and TA10622 (**d**). **e,** Metaplot showing CENH3 ChIP-Seq coverage divided by input control. Genes are grouped by whether they are expressed in the RNA-Seq data. Shaded regions in blue and green indicate ±SD at each interval position for expressed genes, and unexpressed genes, respectively. **f,** Metaplot showing CpG DNA methylation percentage of centromeric genes, genes are again grouped by whether they are expressed. Shaded regions in blue and green indicate ±SD at each interval position for expressed genes, and unexpressed genes, respectively.

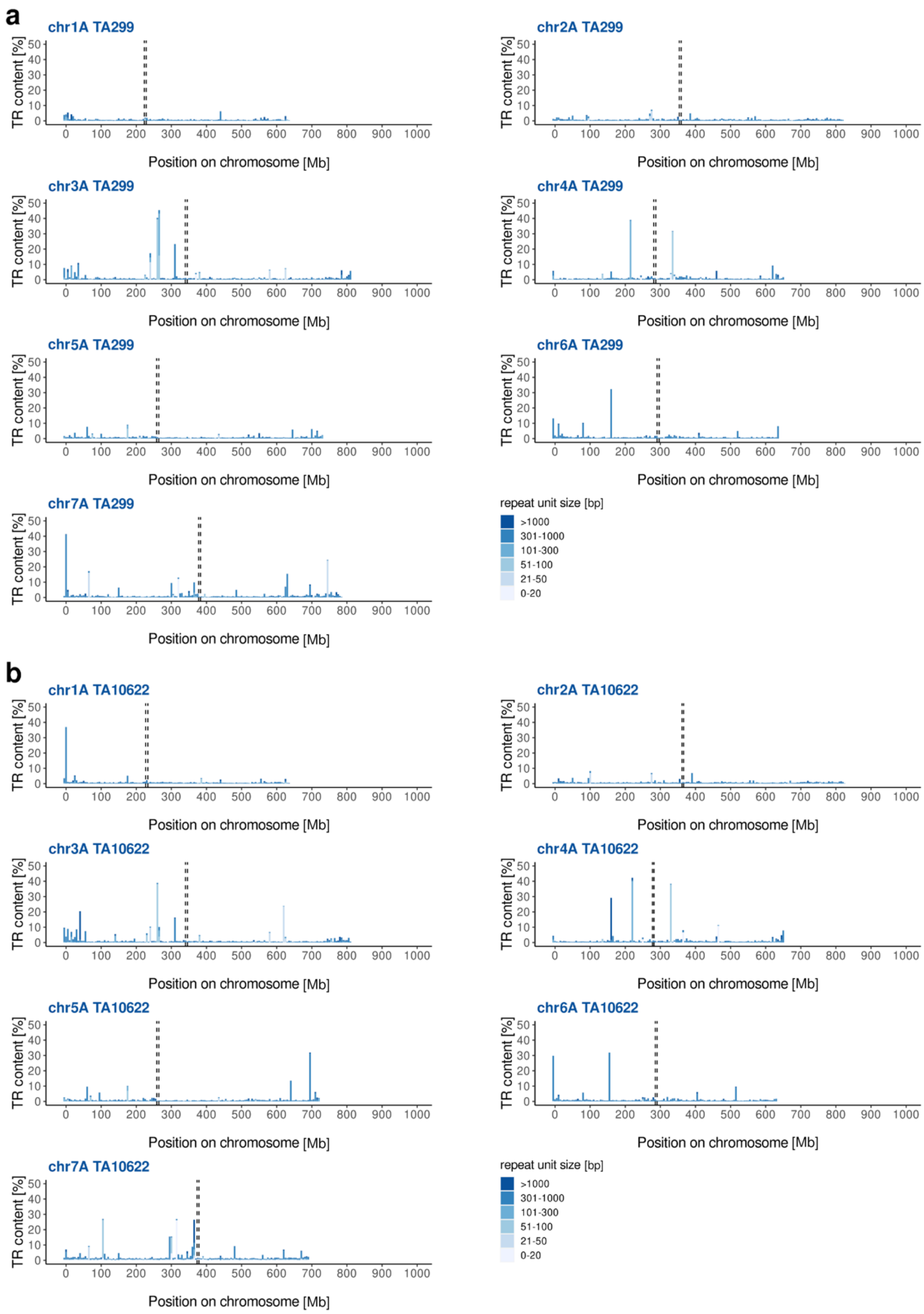

Supplementary Fig. 9. Distribution of tandem repeat (TR) clusters along the chromosomes of TA299 (a) and TA10622 (b) identified by tandem repeats finder. Tandem repeat content is shown in windows of 100 kb. The stacked plots indicate the fraction of DNA that is occupied by tandem repeats of different size ranges (indicated by different colors). Positions of functional centromeres are indicated with vertical dashed lines. Tandem repeats were annotated with the program tandem repeats finder. Overall, the two einkorn assemblies contain 3.67% (TA299) and 3.59% (TA10622) tandemly repeated DNA, slightly higher numbers than were reported for the A subgenome of wheat.

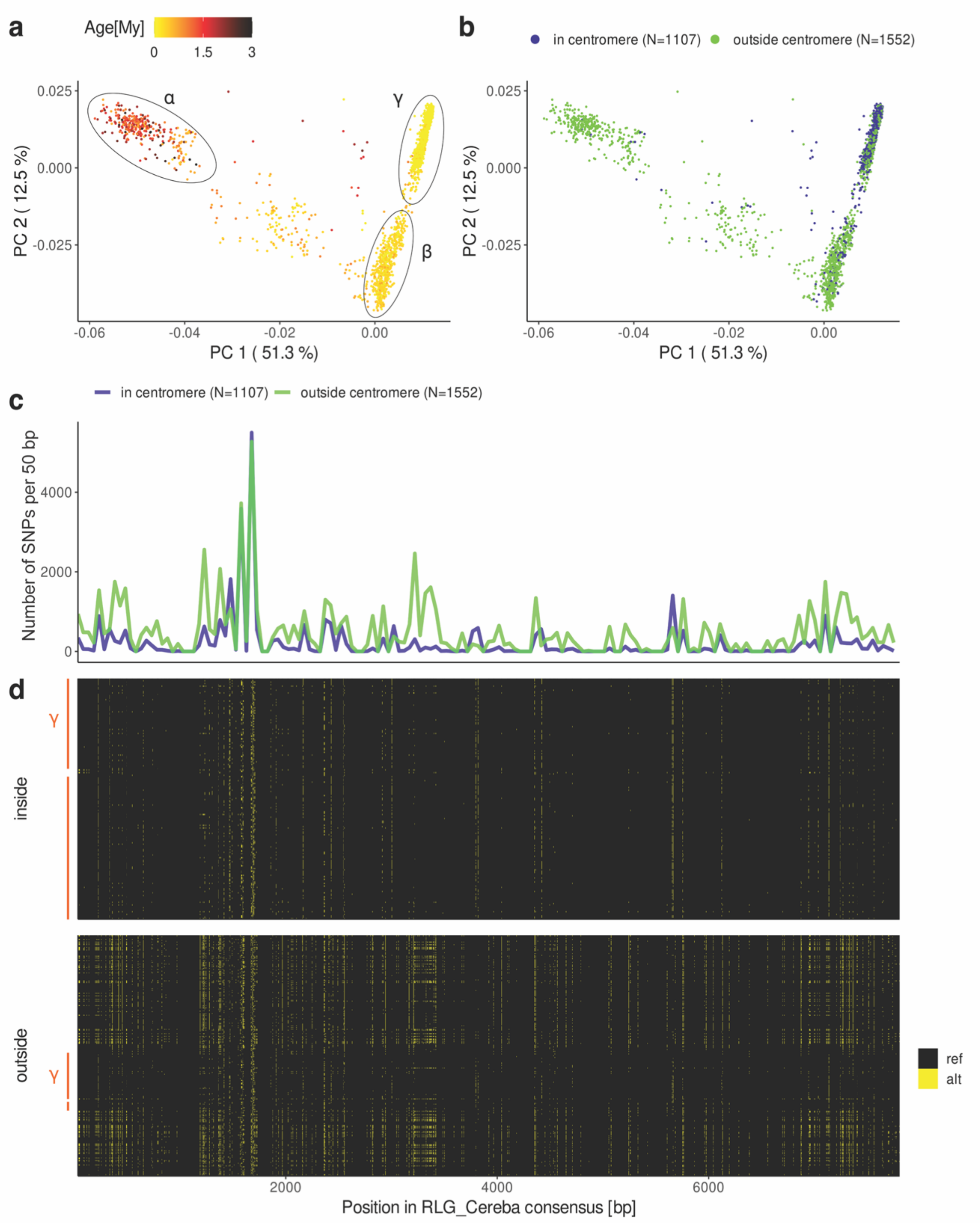

Supplementary Fig. 10. Population analysis of full-length *RLG_Cereba* elements. a, Principal component analysis (PCA) of full length *RLG_Cereba* elements, where each dot corresponds to a single *RLG_Cereba* element. Dots are colored according to TE insertion age. There are three distinct sub-populations, of which the α sub-population represents the oldest TE insertions and the γ sub-population the youngest TE insertions. **b,** PCA with dots colored by location in relation to the functional centromeres. **c,** SNP density of centromeric and non-centromeric *RLG_Cereba* elements compared to the *RLG_Cereba* consensus sequence. **d,** Visualization of SNPs compared to the *RLG_Cereba* consensus sequence for all *RLG_Cereba* elements on chromosome 1A of accession TA299, grouped by location in relation to the functional centromere and ordered within groups by the position on the chromosome. Black lines correspond to the elements carrying the reference variant at this position, whereas yellow lines indicate an alternate base. Members of the γ sub-population are flanked by an orange line.

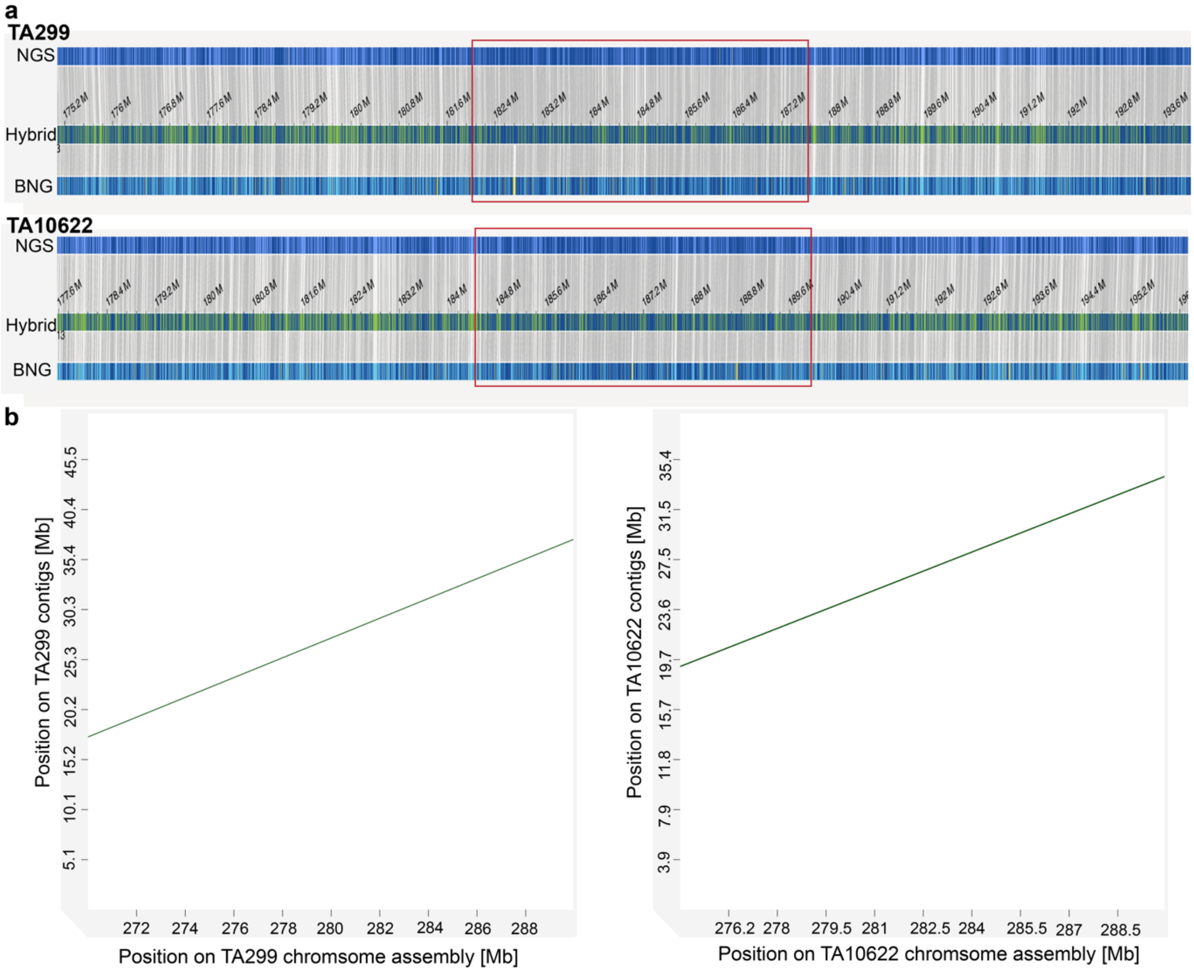

Supplementary Fig. 11. Validation of centromeric region on chromosome 4A of TA299 and TA10622. a, Shown are the hybrid scaffolds of TA299 (upper track) and TA10622 (lower track). The contig-level sequences (NGS) covering the highly rearranged centromere are supported by one continuous bionano optical map (BNG). Functional centromere regions are indicated by red rectangles. **b,** Dot plots between the corresponding contigs in the region and the pseudomolecule assemblies at 270-290 Mb (TA299, on the left) and at 275-290 Mb (TA10622, on the right).

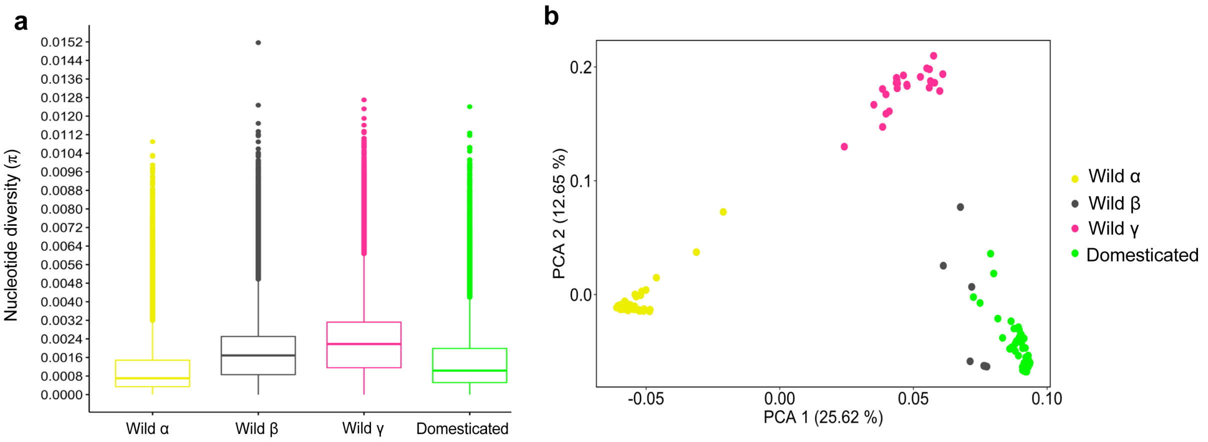

Supplementary Fig. 12. SNP data statistics from SNPs called against the TA299 reference assembly. a, Nucleotide diversity (π) of each einkorn group calculated in sliding windows of 10,000 bp (wild α n=124, wild β n=9, wild γ n=25, domesticated einkorn n=61). Box boundaries indicate the first and third quartile. Lines extending from the boxes (whiskers) indicate variability outside the lower and upper quartiles. The lines in the middle of the boxes represent the median values of π for each group. Outliers are plotted as individual points. **b,** Principal component analysis (PCA) of 218 einkorn accessions using all (121,459,674) SNPs.

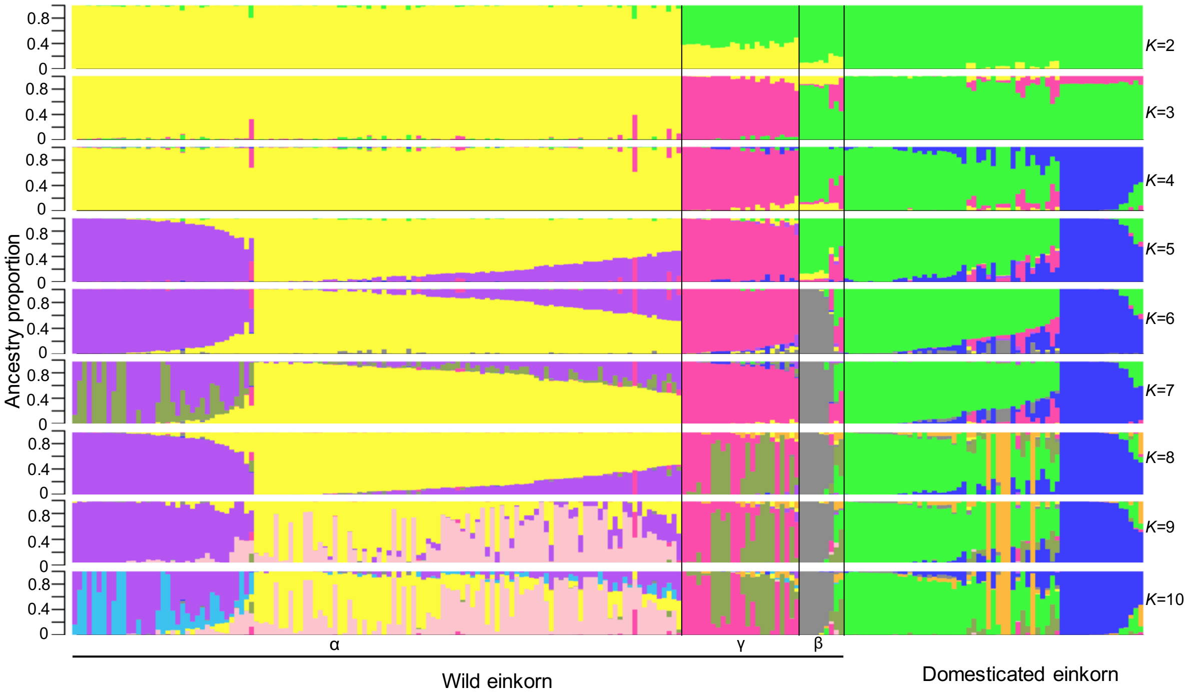

Supplementary Fig. 13. Population structure (from *K*=2 to *K*=10) of einkorn accessions. Each vertical bar represents an accession and the bars are filled by colors representing the proportion of each ancestry.

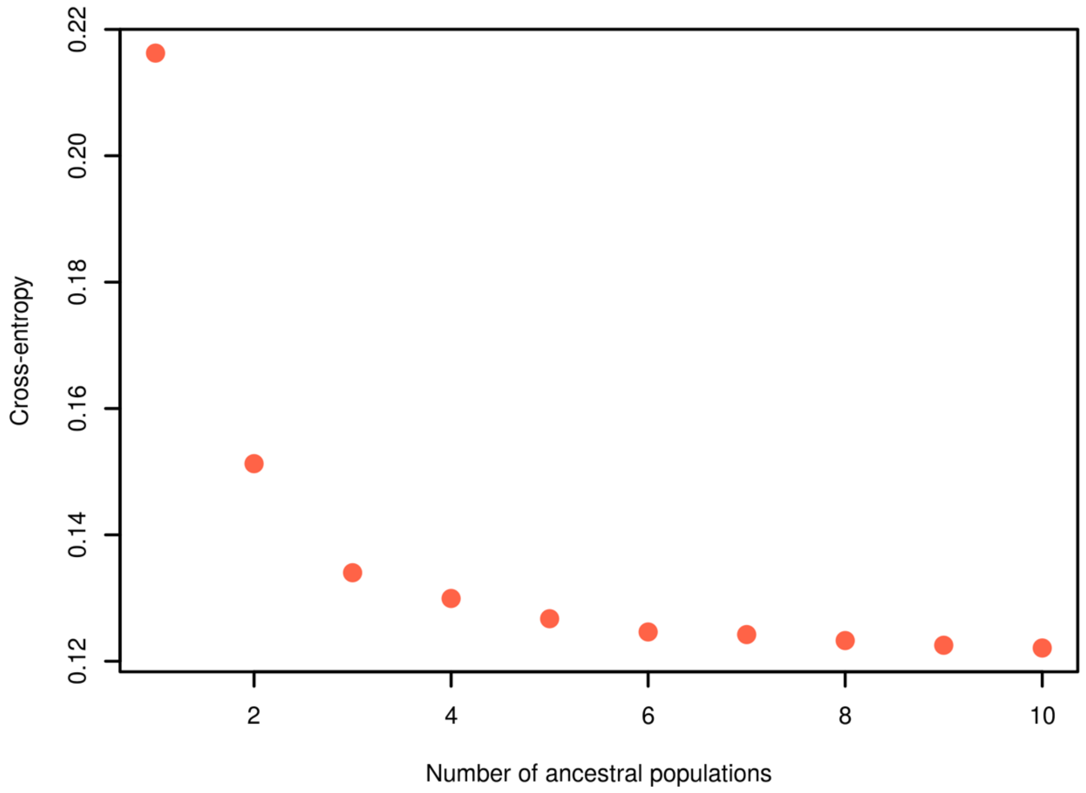

Supplementary Fig. 14. The cross-entropy values for sNMF runs. The cross entropy was calculated for a number of ancestral populations from *K*=1 to *K*=10.

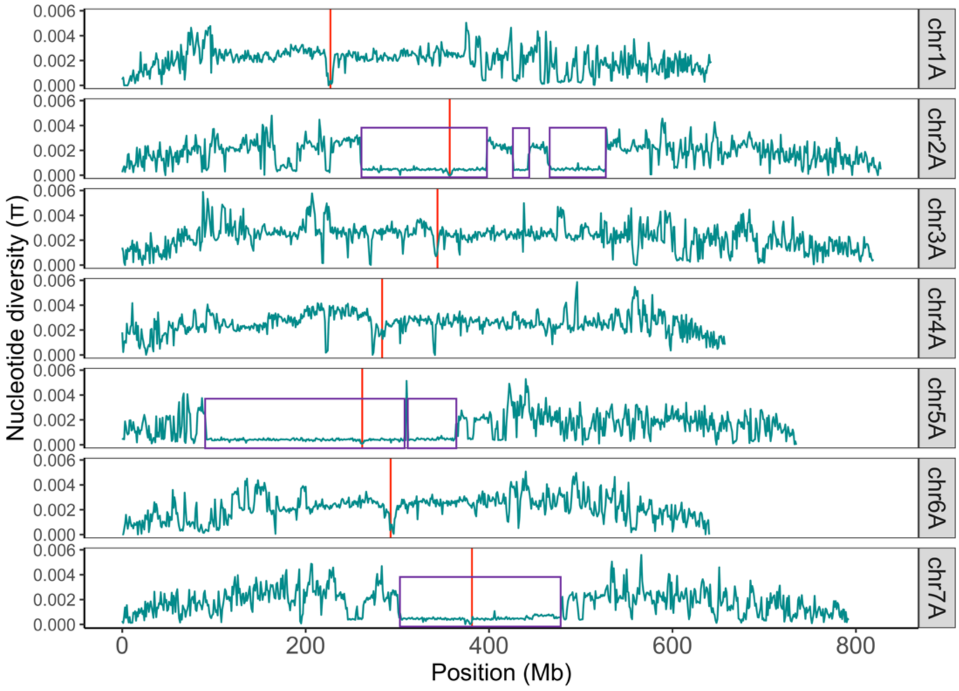

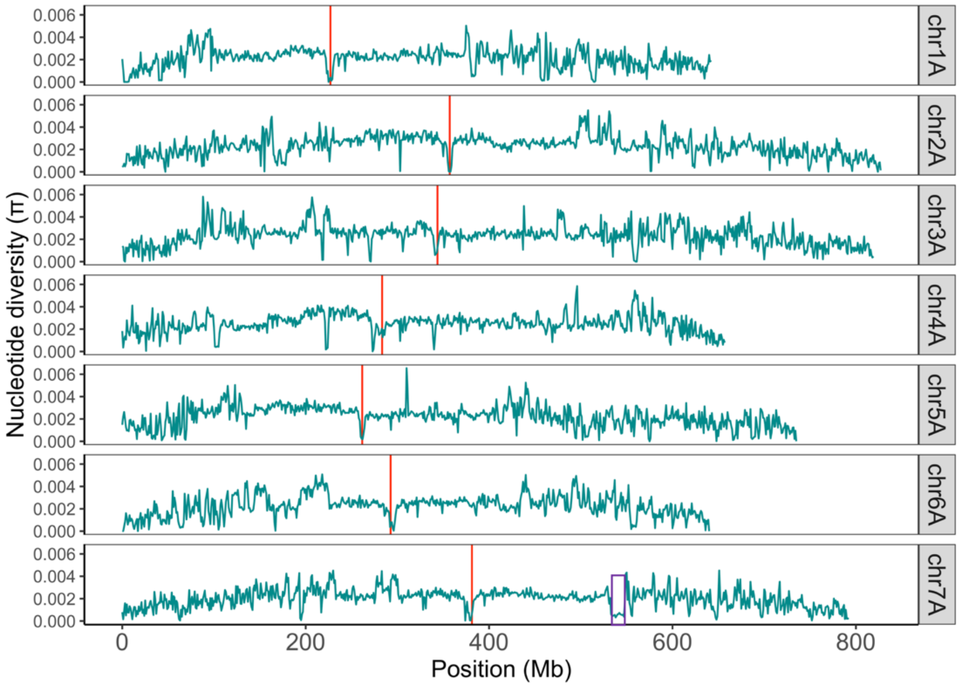

Supplementary Fig. 15. Nucleotide diversity (π) between domesticated einkorn and a wild γ accessions. On the top, nucleotide diversity between TA10588 (domesticated einkorn accession from western Turkey) and TA10600 (wild γ) shows genomic regions with reduced π. On the bottom, nucleotide diversity between TA10604 (domesticated einkorn accession from eastern Turkey) and TA10600 (wild γ). Purple squares indicated the regions with reduced π. See supplementary Table 11.

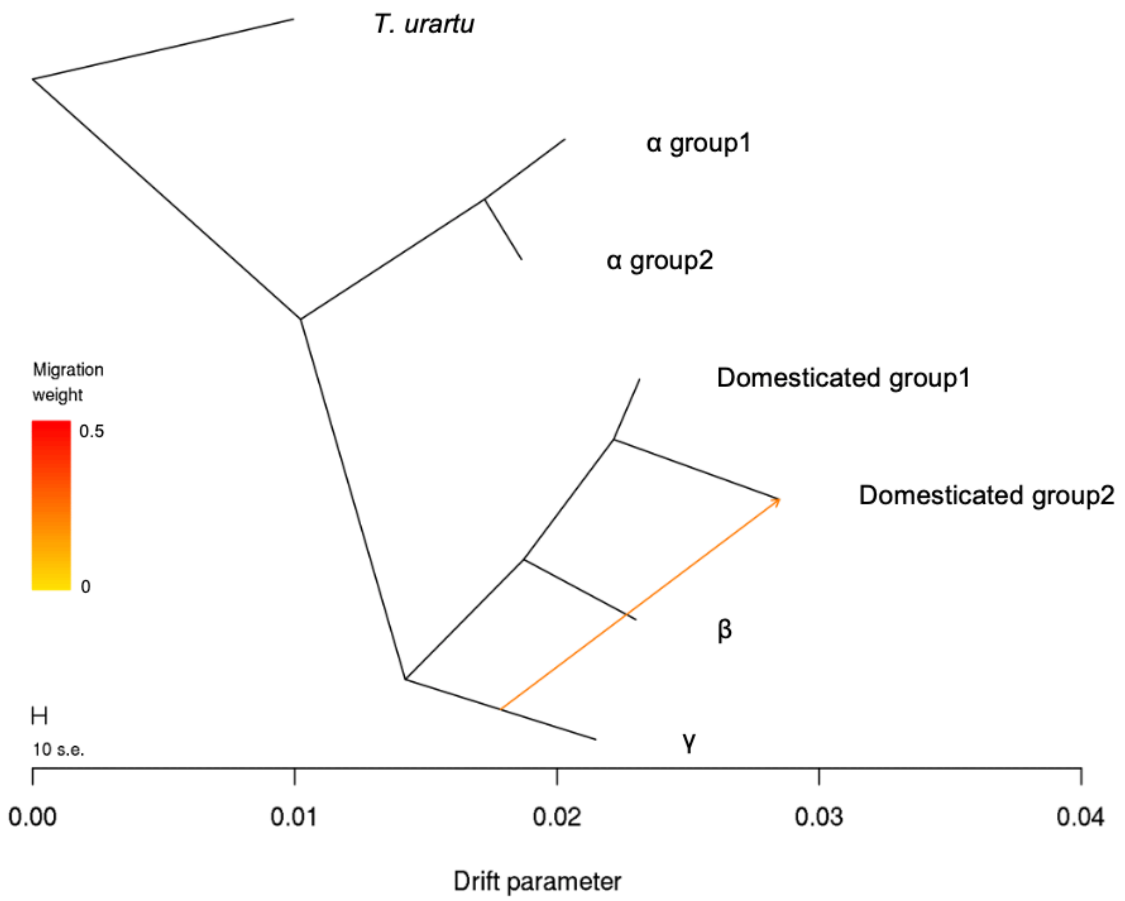

Supplementary Fig. 16. TreeMix analysis between domesticated and wild einkorn groups considering six clusters (*K*=6) identified by sNMF. *T. urartu* was used as an outgroup. The population tree was built assuming one migration event, which explained 99.95% of the variation. The arrow represents an admixture event from γ into domesticated einkorn, colored according to the weight of the inferred edge. *K*=6 was chosen based on the cross-entropy value.

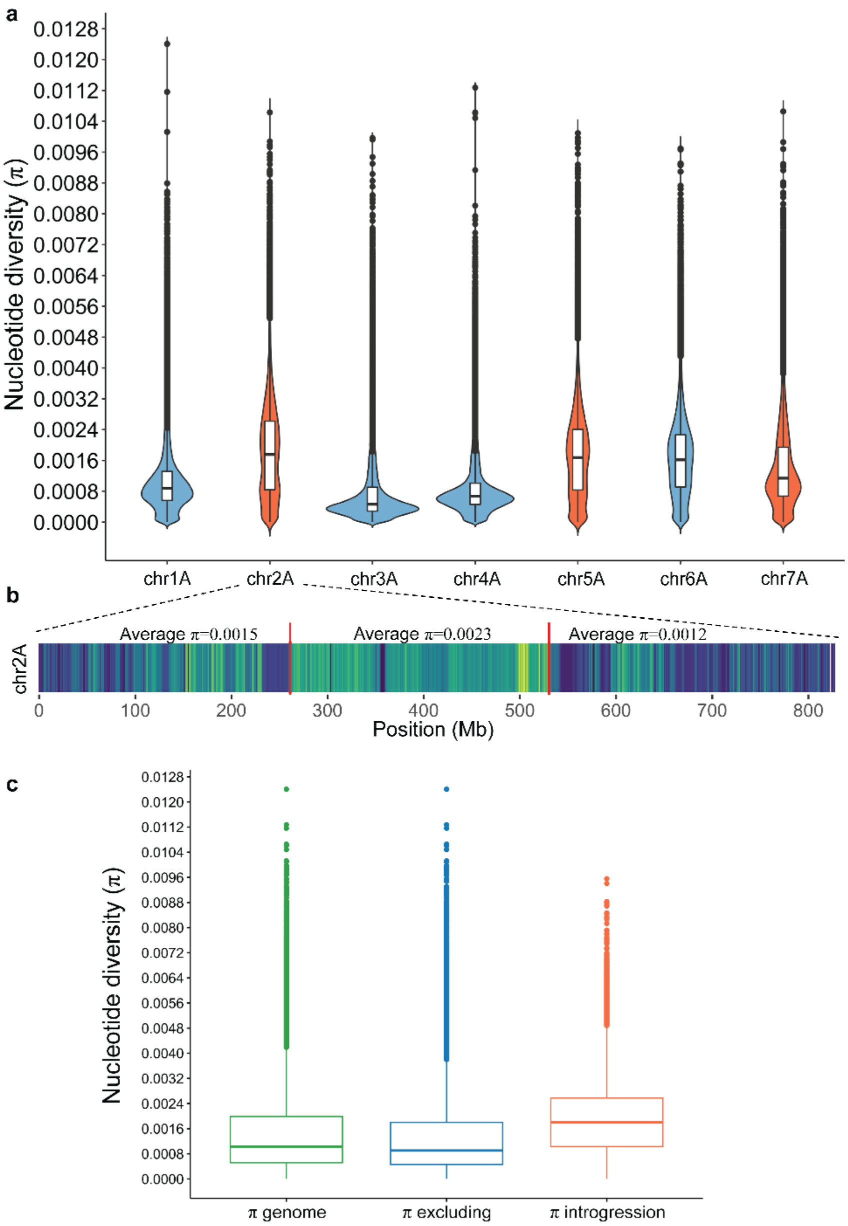

Supplementary Fig. 17. Nucleotide diversity (π) of domesticated einkorn for each chromosome. a, The π was calculated in sliding windows of 10,000 bp (domesticated einkorn n=61). The violin plots show the distribution of the nucleotide diversity (π) of domesticated einkorn accessions per chromosome. Plots colored in orange indicate chromosomes with large introgressed segment from wild einkorn race γ. The center of each plot depicts a boxplot, the box boundaries indicate the first and third quartile. Lines extending from the boxes (whiskers) indicate variability outside the lower and upper quartiles The lines in the middle of the boxes represent the median values of π for each chromosome. Outliers are plotted as individual points. **b,** Nucleotide diversity (π) along chromosome 2A, calculated in 1 Mb non-overlapping windows. The introgressed segment is indicated in the middle between the two red vertical lines. The average π of each of the two regions outside the introgression and the introgression block are indicated above the chromosome heatmap. **c,** Nucleotide diversity (π) calculated in 10-kb non-overlapping windows (n=61) across the entire genome (green; π genome), excluding genomic segments with group γ introgressions, (blue; π excluding), and only including genomics segments with group γ introgressions (orange; π introgression). Box boundaries indicate the first and third quartile. Lines extending from the boxes (whiskers) indicate variability outside the lower and upper quartiles The lines in the middle of the boxes represent the median values of π. Outliers are plotted as individual points.

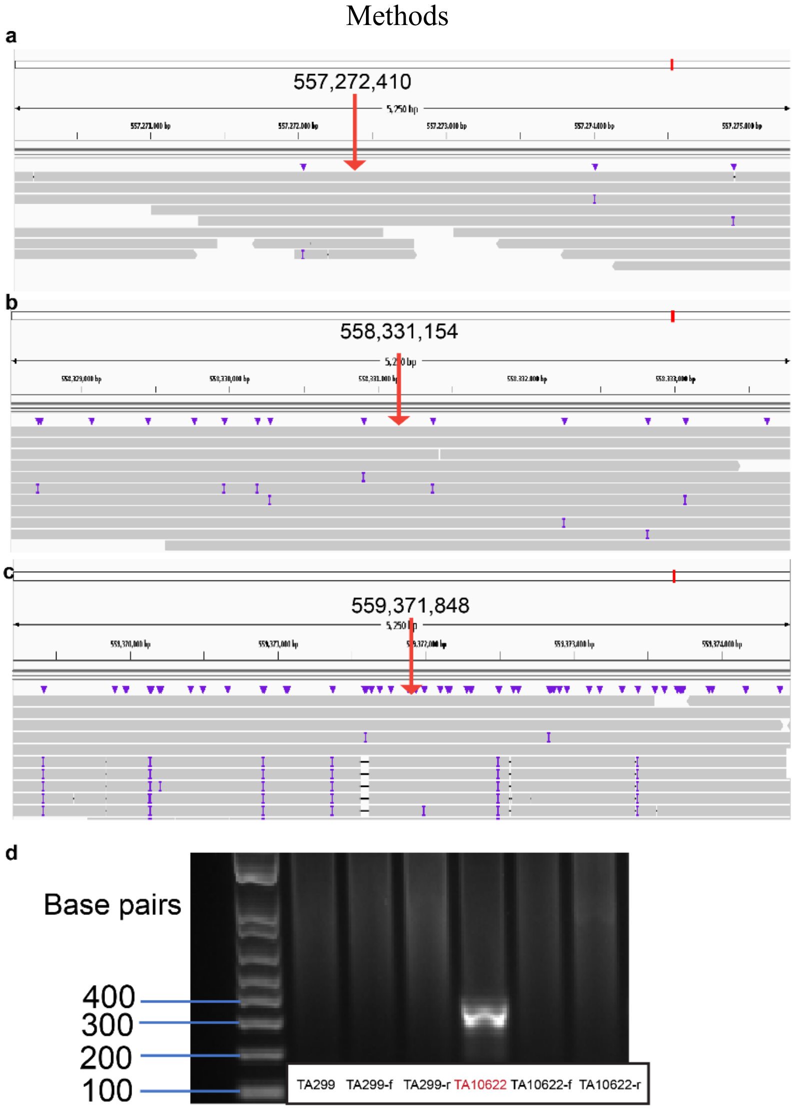

Supplementary Fig. 18. Validation of the 1 Mb tandem duplication. a-c, Mapping of HiFi reads to the TA10622 assembly. Shown are the left breakpoint (**a**), the center breakpoint (**b**), and the right breakpoint (**c**) of the tandem duplication. Only two SMRT cells were used for read mapping. Red arrows indicate the exact location of the breakpoints. **d,** Amplification of a 361 bp PCR fragment spanning the middle breakpoints of the tandem duplication. TA299-f: TA299 using only the forward primer, TA299-r: TA299 using only the reverse primer. The TA10622 sample with both forward and reverse primers shows an amplification at 361 bp. TA10622-f: TA10622 using only the forward primer, TA10622-r: TA10622 using only the reverse primer. Amplification was done with three technical replicates for each primer combination.

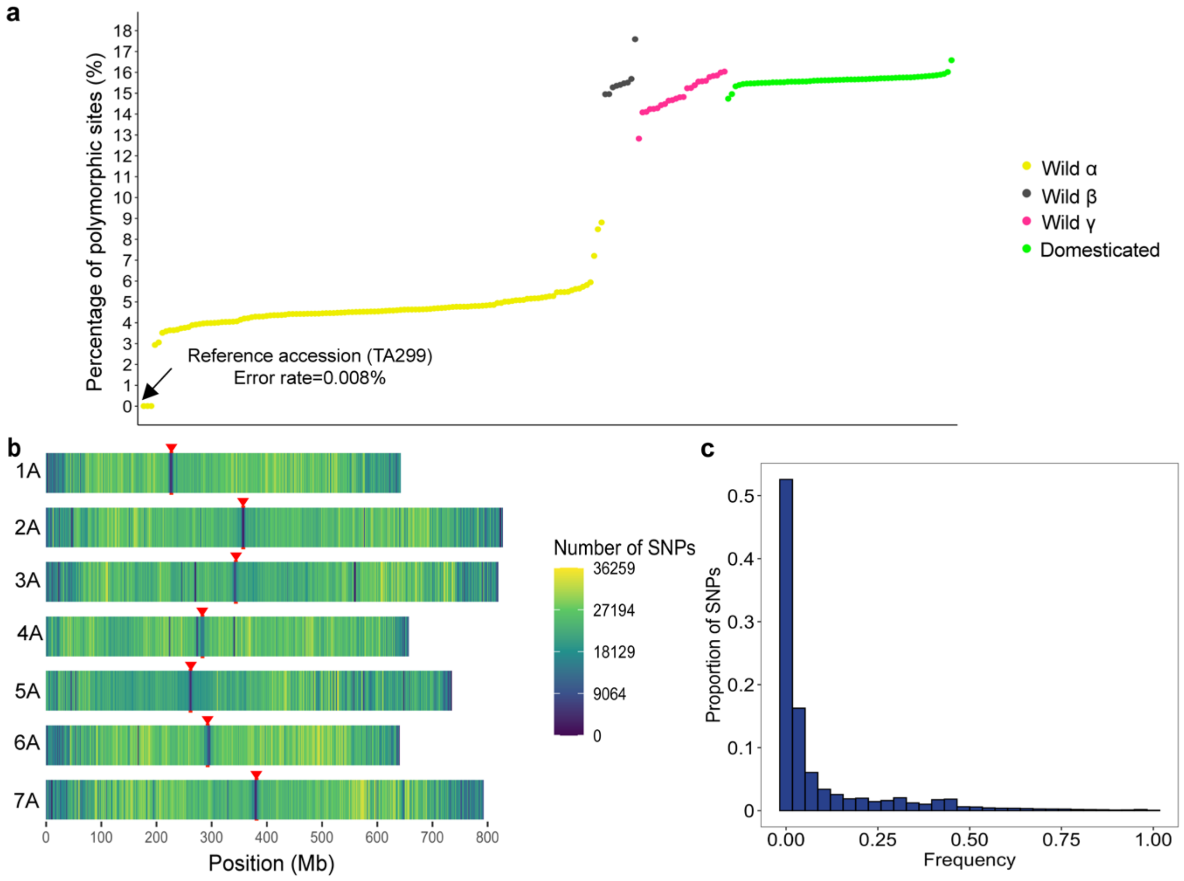

Supplementary Fig. 19. SNP data statistics from SNPs called against the TA299 reference assembly. a, The percentage of polymorphic sites of each einkorn accession compared to the TA299 reference assembly calculated as the proportion of segregating sites. Colors represent the different einkorn groups; race α in yellow, race β in dark gray, race γ in dark pink, domesticated einkorn in green. **b,** SNP density across the seven chromosomes calculated in bin sizes of 1 Mb. Centromeres are indicated by red arrowheads. **c,** Allele frequency of whole-genome sequencing data.

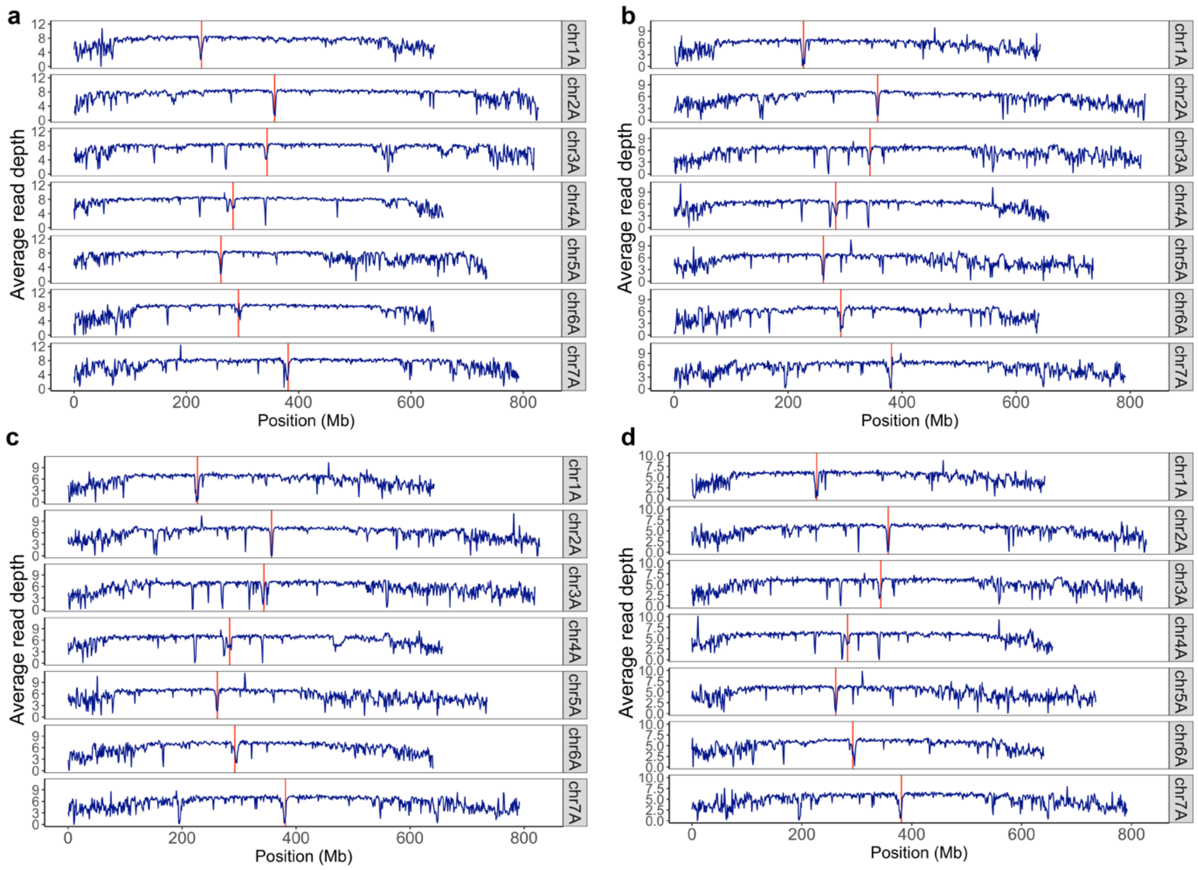

Supplementary Fig. 20. Average read depth in 1 Mb genomic windows. One accession from each group is shown; **a,** Wild einkorn race α (TA10415). **b,** Wild einkorn β race (TA286). **c,** Wild einkorn race γ (TA2005). **d,** Domesticated einkorn accession (TA10604). Centromeres are indicated as vertical red lines. See supplementary Table 16.

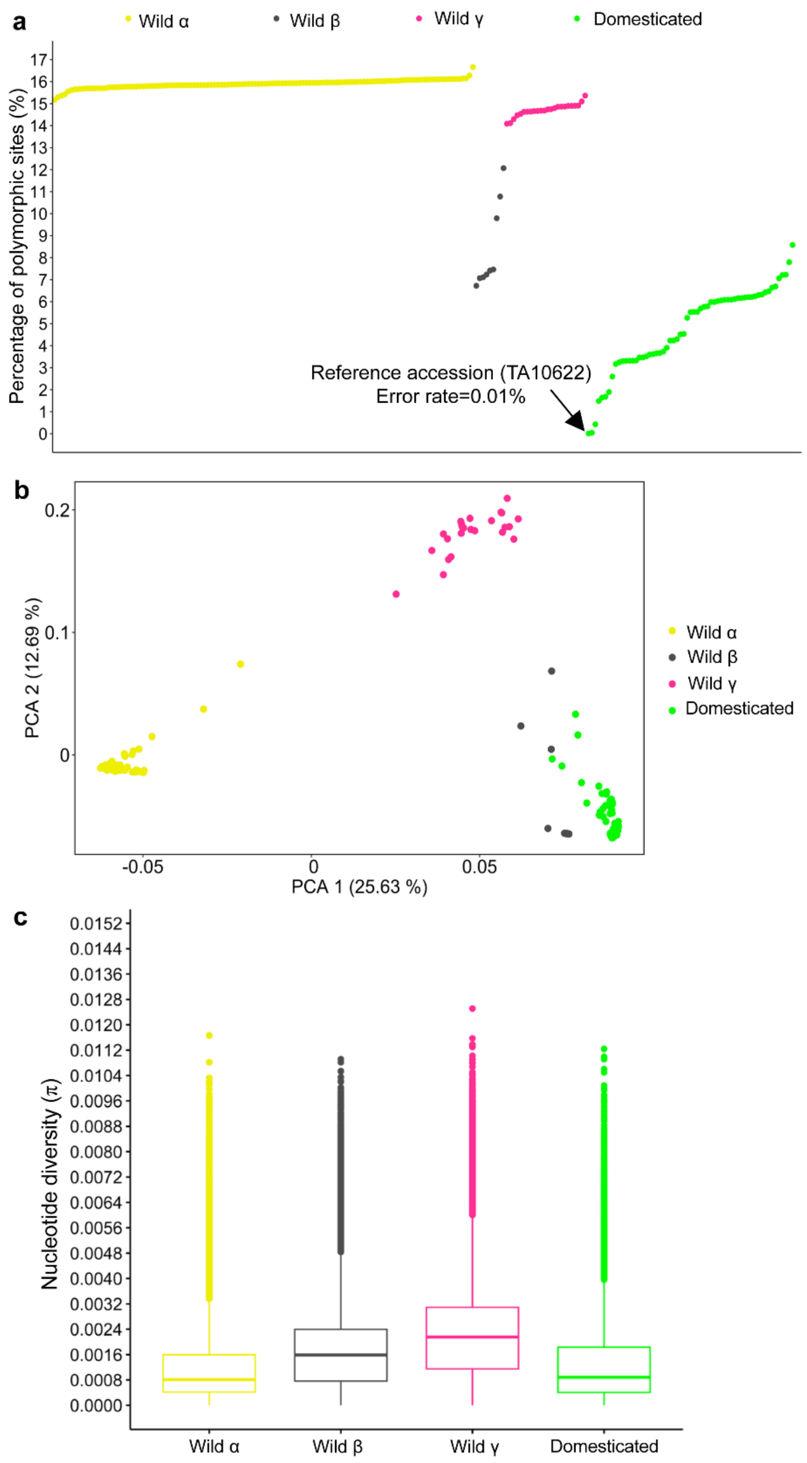

Supplementary Fig. 21. Population genomic analyses of einkorn diversity panel with SNPs called against the TA10622 reference assembly. a, The percentage of polymorphic sites of each einkorn accession compared to the TA10622 reference assembly calculated as the proportion of segregating sites. **b,** Principal component analysis (PCA) of 218 einkorn accessions using all SNPs called against TA10622. Colors represent the different einkorn groups; race α in yellow, race β in dark gray, race γ in dark pink, domesticated einkorn in green. **c,** Nucleotide diversity (π) of each einkorn group calculated in sliding windows of 10,000 bp (wild α n=124, wild β n=9, wild γ n=25, domesticated einkorn n=61). Box boundaries indicate the first and third quartile. Lines extending from the boxes (whiskers) indicate variability outside the lower and upper quartiles The lines in the middle of the boxes represent the median values of π for each group. Outliers are plotted as individual points.

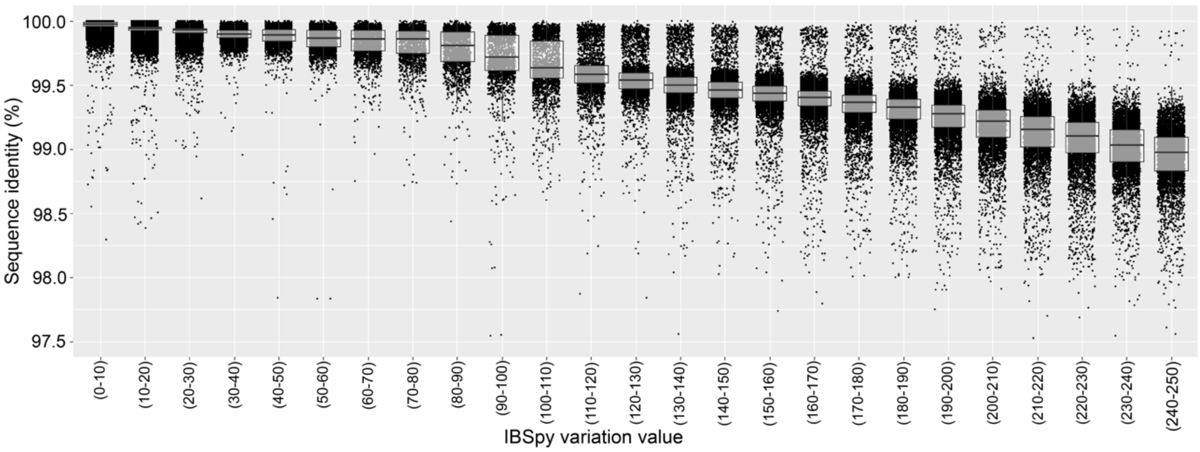

Supplementary Fig. 22. Relationship between IBSpy variation scores and sequence identity. The IBSpy variation scores were grouped in bins of 10 (the x-axis). The y-axis shows the percentage sequence identity of pairwise alignments in the A subgenome chromosomes of hexaploid wheat. The data is filtered for alignments with at least 98% sequence identity across 60% of the 500 kb windows and less than 250 variations per 50 kb. Box boundaries indicate the first and third quartile. The lines in the middle of the boxes represent the median values of the sequence identity for each IBSpy variation value. Number of wheat cultivars included in this analysis (n=10).

Supplementary Table 1. Statistics of the Bionano optical maps and hybrid assemblies.

	TA299	TA10622
**Molecules**		
Filtered data (Gb)	817 Gb	1,255 Gb
Coverage	163x	251x
Molecule N50	234 kb	230 kb
Average label density	15.1 / 100 kb	14.9 / 100 kb
**Optical map assembly**		
Genome map count	123	120
Total genome map length (Mb)	5,124	5,251
Genome map N50 (Mb)	112	164
**Molecules aligned to the assembly**		
Effective coverage of assembly	114x	161x
Average confidence	25.9	26.8
**Hybrid Scaffold assembly**		
Count	22	24
N50 length (Mb)	640.551	522.917
Max length (Mb)	823.635	800.695
Total length (Mb)	5,118.64	5,103.23
NGS in the hybrid scaffold (%)	98.92	99
"N" bases in the hybrid scaffolds (%)	0.02	0.1
Numbers of gaps in the hybrid scaffolds	295	866

Supplementary Table 5. Number of transcripts expressed in the different tissues considering only high-confidence gene models on the seven pseudomolecules. The estimation of expression was done based on TPM (Transcripts Per Million) using the RSEM pipeline.

	TA299		TA10622	
	Expressed	Not expressed	Expressed	Not expressed
Roots	18,826	16,080	19,168	16,622
Aerial part	17,332	17,574	17,624	18,166
Flag-leaf	16,337	18,569	17,192	18,598
Spikes	18,824	16,082	19,700	16,090
Glumes	18,429	16,477	18,805	16,985
Grains	15,107	19,799	15,783	20,007

Supplementary Table 6. Boundaries of functional centromeres as defined by CENH3 sequence read coverage in chromosome assemblies of *T. monococcum* accessions TA299 and TA10622.

**Accession**	**Chromosome**	**Start (Mb)**	**End (Mb)**	Size (Mb)
TA299	chr1A	223.3	228.5	5.2
TA299	chr2A	353.8	359.3	5.5
TA299	chr3A	339.7	345	5.3
TA299	chr4A	281.1	286.8	5.7
TA299	chr5A	258.5	264.3	5.8
TA299	chr6A	290.7	296.4	5.7
TA299	chr7A	377.7	382.7	5
TA10622	chr1A	226.8	232.5	5.7
TA10622	chr2A	361.6	365.6	4
TA10622	chr3A	340.3	345.7	5.4
TA10622	chr4A	277.4	282.7	5.3
TA10622	chr5A	258.8	264.4	5.6
TA10622	chr6A	285.6	291	5.4
TA10622	chr7A	373.1	378.4	5.3

Supplementary Table 14. Corrected genomic regions in both TA299 and TA10622 genome assemblies based on the genetic linkage maps.

Genome	Chromosome	Start (Mb)	End (Mb)	Disagreement description
TA299	chr2A	823	827	Mis-orientation
TA299	chrr4A	1	3	Mis-orientation
TA10622	chr2A	1	3	Mis-orientation
TA10622	chr4A	558	559	Missing genomic segment (was in ChrUn)

Supplementary Table 15. Number of SNPs retained after each filtering step.

Filtering step	Number of SNPs (TA299)	Number of accessions
1- Hard filter (raw SNPs)	208,855,939	219
2- SNP clusters	146,257,318	219
3- Minimum and maximum mean depth	132,348,152	219
4- Bi-allelic SNPs	128,060,262	219
5- SNPs in unanchored chromosome	127,988,888	219
6- Remove accession - TA574	121,459,674	218

References

119. Benson, G. Tandem repeats finder: a program to analyze DNA sequences. *Nucleic Acids Research* **27**, 573-580 (1999).
120. Naish, M. *et al.* The genetic and epigenetic landscape of the *Arabidopsis* centromeres. *Science* **374**, eabi7489 (2021).
121. Cheng, Z. *et al.* Functional rice centromeres are marked by a satellite repeat and a centromere-specific retrotransposon. *The Plant Cell* **14**, 1691-1704 (2002).
122. Schnable, P.S. *et al.* The B73 maize genome: complexity, diversity, and dynamics. *Science* **326**, 1112-1115 (2009).
123. International Brachypodium Initiative. Genome sequencing and analysis of the model grass *Brachypodium distachyon*. *Nature* **463**, 763-768 (2010).
124. Wolfgruber, T.K. *et al.* Maize centromere structure and evolution: sequence analysis of centromeres 2 and 5 reveals dynamic loci shaped primarily by retrotransposons. *PLOS Genetics* **5**, e1000743 (2009).
125. Su, H. *et al.* Centromere satellite repeats have undergone rapid changes in polyploid wheat subgenomes. *The Plant Cell* **31**, 2035-2051 (2019).
126. Cheng, Z.-J. & Murata, M. A centromeric tandem repeat family originating from a part of Ty3/*gypsy*-retroelement in wheat and its relatives. *Genetics* **164**, 665-672 (2003).
127. Walsh, J.B. Persistence of tandem arrays: implications for satellite and simple-sequence DNAs. *Genetics* **115**, 553-567 (1987).
128. Sharma, A., Wolfgruber, T.K. & Presting, G.G. Tandem repeats derived from centromeric retrotransposons. *BMC Genomics* **14**, 142 (2013).
129. Wicker, T., Guyot, R., Yahiaoui, N. & Keller, B. CACTA transposons in Triticeae. A diverse family of high-copy repetitive elements. *Plant Physiology* **132**, 52-63 (2003).
130. Kokot, M., Długosz, M. & Deorowicz, S. KMC 3: counting and manipulating *k*-mer statistics. *Bioinformatics* **33**, 2759-2761 (2017).
131. Walkowiak, S. *et al.* Multiple wheat genomes reveal global variation in modern breeding. *Nature* **588**, 277-283 (2020).
132. Brinton, J. *et al.* A haplotype-led approach to increase the precision of wheat breeding. *Communications Biology* **3**, 712 (2020).
