# Supplementary material for: Einkorn genomics sheds light on history of the oldest domesticated wheat
Source: Nature. 2023 Aug 2;620(7975):830–8. doi: 10.1038/s41586-023-06389-7 (PMC10447253; doi:10.1038/s41586-023-06389-7)
Supplement: Supplementary file 2 — Reporting Summary [file 41586_2023_6389_MOESM2_ESM.pdf]

nature portfolio

Corresponding author(s): Vijay K. Tiwari
Michael Abrouk
Jesse Poland
Simon G. Krattinger

Last updated by author(s): 18 June 2023

Reporting Summary

Nature Portfolio wishes to improve the reproducibility of the work that we publish. This form provides structure for consistency and transparency in reporting. For further information on Nature Portfolio policies, see our Editorial Policies and the Editorial Policy Checklist.

Statistics

For all statistical analyses, confirm that the following items are present in the figure legend, table legend, main text, or Methods section.

n/a	Confirmed	
☐	☒	The exact sample size (*n*) for each experimental group/condition, given as a discrete number and unit of measurement
☐	☒	A statement on whether measurements were taken from distinct samples or whether the same sample was measured repeatedly
☐	☒	The statistical test(s) used AND whether they are one- or two-sided<br>*Only common tests should be described solely by name; describe more complex techniques in the Methods section.*
☒	☐	A description of all covariates tested
☒	☐	A description of any assumptions or corrections, such as tests of normality and adjustment for multiple comparisons
☐	☒	A full description of the statistical parameters including central tendency (e.g. means) or other basic estimates (e.g. regression coefficient) AND variation (e.g. standard deviation) or associated estimates of uncertainty (e.g. confidence intervals)
☐	☒	For null hypothesis testing, the test statistic (e.g. *F*, *t*, *r*) with confidence intervals, effect sizes, degrees of freedom and *P* value noted<br>*Give P values as exact values whenever suitable.*
☒	☐	For Bayesian analysis, information on the choice of priors and Markov chain Monte Carlo settings
☒	☐	For hierarchical and complex designs, identification of the appropriate level for tests and full reporting of outcomes
☒	☐	Estimates of effect sizes (e.g. Cohen's *d*, Pearson's *r*), indicating how they were calculated

Our web collection on statistics for biologists contains articles on many of the points above.

Software and code

Policy information about availability of computer code

Data collection	Whole genome sequencing of 218 einkorn accessions was done using Illumina NovaSeq platforms. Sequencing reads for genome assemblies were generated using PacBio circular consensus sequencing. Optical maps were generated using the Bionano Genomics Saphyr System. Omni-C reads were sequenced with Illumina NovaSeq platforms. RNA-Seq and Iso-Seq for six different tissues for two einkorn accessions were generated with Illumina NovaSeq platforms and PacBio circular consensus sequencing. CENH3 and H3K4me3 data were generated in this study.<br>The TREP database (v19) was used to obtain repeat information.<br>Translated proteins of Triticum urartu, Aegilops tauschii, wild emmer (Zavitan), hexaploid wheat (Kariega and ArinaLrFor), barley (Morex version 3), Brachypodium distachyon, rice, and the Triticeae and Poaceae protein sequences downloaded from the UniProt database (2021_03) were used for gene model prediction.
Data analysis	The software and tools used in this study are as follows:<br>Bionano Solve (v.3.6), hifiasm (v15.1), Juicer (v1.6), 3D-DNA (v180922), Juiecbox (v1.11.08), BUSCO (v5.0.0), Merqury (v1.3), SAMtools (v1.8), BCFtools (v1.9), JoinMap (v5.0), Mapchart (v2.32), STAR (v2.7.0f and v2.5.2a), Stringtie (v2.1.4), minimap2 (v2.21), cDNA_Cupcake (v12.4.0), Transdecoder (v5.5.0), BRAKER2 (v2.1.2), FgeneSH (v8.0.0), EDTA, GenomeThreader (v1.7.1), EVidenceModeler (v1.1.1), PASA pipeline (v2.5.1), DIAMOND (v2.0.9), MCScanX, Circos software (v0.69-9), Gepard, bowtie2, MUMmer (v4.0.0.2), Tandem Repeats Finder (v4.09.1), BLASTn (2.11.0+), trimmomatic (v0.38), BWA mem (v0.7.17), Picard tools, GATK (v4.1.8.0), VCFtools (v0.1.17), PLINK (v1.90), TreeMix (v1.13), jellyfish (v2.2.10), featureCounts (v2.0.0), ccsmeth (v0.3.2), InterproScan (vv5.55-88.0), deepTools, epic2 peak caller<br><br>R packages used in this study are as follows:<br>stats v4.1.3, LEA v2.0, fields v10.3, ggplot2 v3.3.6

Custom pipelines or scripts generated and used in this study:
Centromere comparison: (https://github.com/Wicker-Lab/Monococcum_genome_scripts)
IBSpy pipeline(Identity-by-State python; https://github.com/Uauy-Lab/IBSpy).
Wheat SNP calling scripts (https://github.com/IBEXCluster/Wheat-SNPCaller).
Codes and custom scripts for analyzing einkorn introgressions into bread wheat (https://github.com/Uauy-Lab/monococcum_introgressions).

Custom codes used to process data for genetic linkage maps are as follows:
Perl scripts for demultiplexing of raw FASTQ files (https://github.com/sandeshsth/SkimSeq_Method and https://github.com/sandeshsth/Fastq).
Codes and steps to generate RIL population: (https://datadryad.org/stash/share/v20dkVsSTj3toGn-CHG92eUSgre17uMT5AH_6LE2GDM).

For manuscripts utilizing custom algorithms or software that are central to the research but not yet described in published literature, software must be made available to editors and reviewers. We strongly encourage code deposition in a community repository (e.g. GitHub). See the Nature Portfolio guidelines for submitting code & software for further information.

Data

Policy information about availability of data

All manuscripts must include a data availability statement. This statement should provide the following information, where applicable:

- Accession codes, unique identifiers, or web links for publicly available datasets
- A description of any restrictions on data availability
- For clinical datasets or third party data, please ensure that the statement adheres to our policy

The raw sequence data of T. urartu was downloaded from the Sequence Read Archive (https://www.ncbi.nlm.nih.gov/sra) accession number PRJNA663409.
The ten bread wheat assemblies were downloaded from https://wheat.ipk-gatersleben.de/

Data availability
The raw sequencing data used for de novo genome assemblies, the RNA-Seq and Iso-Seq data for the annotation, the ChIP-Seq reads, and the whole-genome sequencing reads of 218 einkorn accessions are available on EBI_ENA under study number PRJEB61155. The two reference assemblies, annotations, VCF files, and CpG methylation frequencies are available on DRYAD [https://doi.org/10.5061/dryad.v41ns1rxj]. Raw fastq files and demultiplexed fastq files of the RIL population have been deposited at the National Center for Biotechnology Information (NCBI) SRA database with the BioProject accession PRJNA879879. The barcode indices key file with required information for demultiplexing can be obtained at DRYAD [https://doi.org/10.5061/dryad.v41ns1rxj]. CENH3 BED files (CENH3 peaks), and mapped files (bam) of CENH3 and H3K4me3 for all replicates are available through the Dryad database: [https://doi.org/10.5061/dryad.0p2ngf24b]. Whole genome sequencing data of the tin3 mutant bulk has been deposited into GenBank under BioProject PRJNA938447. The source data underlying Supplementary Figures 15, and 20, as well as Extended Data Fig. 9 are provided as a Source Data file. IBSpy variations tables of the 218 einkorn accessions, and MUMer alignments of the two einkorn assemblies against the 10 bread wheat assemblies are available through the following link: [https://opendata.earlham.ac.uk/wheat/under_license/toronto/Uauy_2022-09-24_IBSpy_Triticum_monococcum_introgressions/].
An interactive webpage has been developed for this study to visualize various genome characteristics of TA299 and TA10622. The webpage features a JBrowse 2 explorer allowing visualization of the whole genome, gene models, transposable elements, variants positions and synteny. For the identification of homologous sequences in other wheat varieties, a BLAST server has been set up. This BLAST server allows searches against individual wheat subgenomes and chromosomes independently. Synteny between TA10622 and other wheat genomes can be visualized in the synteny tab located on the webpage. The database can be accessed through the following link: https://avena.pw.usda.gov/genomes/mono

Human research participants

Policy information about studies involving human research participants and Sex and Gender in Research.

Reporting on sex and gender	no human research participants
Population characteristics	N/A
Recruitment	N/A
Ethics oversight	N/A

Note that full information on the approval of the study protocol must also be provided in the manuscript.

Field-specific reporting

Please select the one below that is the best fit for your research. If you are not sure, read the appropriate sections before making your selection.

☒ Life sciences ☐ Behavioural & social sciences ☐ Ecological, evolutionary & environmental sciences

For a reference copy of the document with all sections, see nature.com/documents/nr-reporting-summary-flat.pdf

Life sciences study design

All studies must disclose on these points even when the disclosure is negative.

Sample size	A recombinant inbred line (RIL) population consisting of 827 lines were used to construct the genetic maps. The larger the sample size is, the better in constructing the genetic map. The first set originally developed by Singh et al. (2007) [https://pubmed.ncbi.nlm.nih.gov/17565482/] consisted of 93 samples, including three blanks, and was sequenced at 0.2x coverage. The second set comprised 733 samples, sequenced at 0.03x coverage, with 35 blanks included as controls. In the skim-seq panel, we also included five replicates of each of the two RIL parents along with the RILs.<br>The number of plants (n) for the tin3 analysis were as follows: Jagger (n = 20), tin3A (n=12), tin3B (n=6), tin3D (n=14), tin3AB (n=7), tin3AD (n=8), tin3BD (n=12), and tin3ABD (n=8). These samples were used for Tukey's HSD statistics.<br>No statistical methods were used to establish sample sizes for genome assemblies and whole genome sequencing data for the einkorn diversity panel. Two einkorn accessions were selected for genome assemblies. A total of 218 of wild and domesticated einkorn accessions were used for the population genomics analyses. The chosen accessions cover a wide range of geographic and genetic diversity distribution of einkorn, which allowed to understand crop diversity and population structure in details. The einkorn accessions were selected from a larger diversity panel comprising 733 accessions and they were chosen based on genotyping-by-sequencing data (Reference: Adhikari et al., Genetic characterization and curation of diploid A-genome wheat species, Plant Physiology(2022)). A total of 6 different plant tissues per accession genome were used to extract RNA for RNA-Seq and Iso-Seq.
Data exclusions	218 out of 219 einkorn accessions were used for population genomics analysis. One accession was removed from the analysis due to mis-classification (i.e, this accession was not einkorn).
Replication	For validating the tandem duplication in the reference genome assembly TA10622, three different technical replicates were used for each primer combination.<br>For the positional cloning of tin3 experiment: TA4342-L96 and tin3 mutants have been phenotyped four independent times with 15-20 plants per genotype each time. The SEM experiment was repeated three times.<br>For ChIP-Seq experiment, two replicates have been used, all attempts were successful
Randomization	Randomization were not needed for this study as the study focuses on establishing and analyzing genomic resources and perform population genomics analyses. Our study does not include different treatment groups. Randomization is important to ensure that the allocation of participants or samples to different treatment groups is unbiased and free from systematic biases which in this case is not applicable to our study.
Blinding	Blinding does not apply to this study, as the main focus of our study is on the observational design, were we collect genetic data from individuals or populations without intervening or manipulating any variables. Our study does not invlove treatment or intervention being administered that would require blinding.

Reporting for specific materials, systems and methods

We require information from authors about some types of materials, experimental systems and methods used in many studies. Here, indicate whether each material, system or method listed is relevant to your study. If you are not sure if a list item applies to your research, read the appropriate section before selecting a response.

Materials & experimental systems

n/a	Involved in the study	
☐	☒	Antibodies
☒	☐	Eukaryotic cell lines
☒	☐	Palaeontology and archaeology
☒	☐	Animals and other organisms
☒	☐	Clinical data
☒	☐	Dual use research of concern

Methods

n/a	Involved in the study	
☐	☒	ChIP-seq
☒	☐	Flow cytometry
☒	☐	MRI-based neuroimaging

Antibodies

Antibodies used	ChIP-Seq:<br>Nuclei were isolated from 2-week-old seedlings and digested with micrococcal nuclease (Sigma) to liberate nucleosomes. The digested mixture was incubated overnight with 3 ug of antibody at 4oC. The target antibodies were captured from the mixture using Dynabeads Protein G (Invitrogen, Carlsbad, CA) to obtain ChIP DNA. Mock DNA control was maintained with the input DNA following the same conditions above without antibodies. The ChIP experiments were performed with two biological replications. Library construction was performed using the TruSeq ChIP Sample Prep Kit (Illumina, San Diego, CA) according to the manufacturer’s instructions.<br><br>Antigen with the peptide sequence ‘RTKHPAVRKTKALPKK’ corresponding to the N-terminus of wheat CENH3 was used to produce antibody utilizing the custom-antibody production facility provided by the Thermo Fisher Scientific, Illinois, USA (abs@thermofisher.com). A 0.396 mg of customized antibody was purified and obtained as pellet. The pellet was dissolved in 2 ml of PBS buffer, pH 7.4 resulting in 198 ng/ul of CENH3 antibody. Fifteen microliters of anti-CENH3 antibody was used for chromatin

	immunoprecipitation (ChIP).<br><br>Anti-trimethyl-Histone H3 (Lys4) (H3K4me3) antibody (Cat.# 07-473) was purchased from Sigma (St. Louis, MO). A 3 ul of anti-H3K4me3 antibody was used for ChIP.
Validation	The specificity of anti-CENH3 and anti-H3K4me3 antibodies were validated using immunofluorescence assay on mitotic and meiotic chromosomes of diploid (Triticum monococcum) and hexaploid (Triticum aestivum) wheat.

ChIP-seq

Data deposition

☒ Confirm that both raw and final processed data have been deposited in a public database such as GEO.

☒ Confirm that you have deposited or provided access to graph files (e.g. BED files) for the called peaks.

Data access links<br>*May remain private before publication.*	The raw sequencing data has been deposited to the EBI_ENA database under study number PRJEB61155<br>BED files (CENH3 peaks), and mapped files (bam) for both CENH3 and H3K4me3 are in Dryad database: https://doi.org/10.5061/dryad.0p2ngf24b.
Files in database submission	Samples names in EBI_ENA (project ID: PRJEB61155) are as follow: TA299-C1-CenH3_ChiP_replicat1, TA299-C2-CenH3_ChiP_replicat2, TA299-M1-CenH3_DNA_input_control_replicate1, TA299-M2-CenH3_DNA_input_control_replicate2, TA10622-C1-CenH3_ChiP_replicat1, TA10622-C2-CenH3_ChiP_replicat2, TA10622-M1-CenH3_DNA_input_control_replicate1, TA10622-M2-CenH3_DNA_input_control_replicate2, TA299-C1-H3K4me3_ChiP_replicat1, TA299-C2-H3K4me3_ChiP_replicat2, TA299-M1-H3K4me3_DNA_input_control_replicate1, TA299-M2-H3K4me3_DNA_input_control_replicate2.<br>The Dryad link contains BED files, and mapped files (.bam)
Genome browser session<br>(e.g. UCSC)	No longer applicable

Methodology

Replicates	The ChIP experiments were performed with two independent biological replicates. The ChIP-Seq profile map of the replicates were identical.
Sequencing depth	The CENH3 ChIP-Seq reads represent ~ 4x coverage per genome.
Antibodies	Wheat CENH3 antibody as described here: Koo DH, Sehgal SK, Friebe B, Gill BS (2015) Structure and stability of telocentric chromosomes in wheat. PLoS One 10: e0137747.
Peak calling parameters	The epic2 peak caller was used to identify peaks of CENH3 enrichment with a MAPQ≥30 filtering and with a resolution of 100 kb
Data quality	Quality filtering and adapter sequence removal were done with trimmomatic. Duplicates were removed using SAMtools. Secondary alignments (i.e. multi-mapping reads) were removed using the flag -F 0x0100 with SAMtools. Ratio of ChIP/input coverage was calculated using the deeptools function bamCompare using MAPQ≥30 as a threshold
Software	Trimmomatic, bowtie2, SAMtools, deeptools (function bamCompare), epic2 peak caller
