# Supplementary material for: Einkorn genomics sheds light on history of the oldest domesticated wheat
Source: Nature. 2023 Aug 2;620(7975):830–8. doi: 10.1038/s41586-023-06389-7 (PMC10447253; doi:10.1038/s41586-023-06389-7)
Supplement: Supplementary file 3 — Peer Review File [file 41586_2023_6389_MOESM3_ESM.pdf]

Peer Review File

Manuscript Title: Einkorn genomics sheds light on history of the oldest domesticated wheat

Reviewer Comments & Author Rebuttals

Reviewer Reports on the Initial Version:

Referees' comments:

Referee #1 (Remarks to the Author):

In their manuscript, Ahmed and colleagues describe their high-quality genome assemblies of wild and domesticated einkorn wheat. These assemblies are state-of-the-art, showing high levels of contiguity and completeness. This is a substantial step forward for wheat genomics as previous efforts toward genome assembly have been challenged by the exceptional abundance of repetitive sequences and the large genome sizes of the species. While einkorn wheat is domesticated itself, it is also closely related to a genome donor to both tetraploid and hexaploid domesticated wheat species, making assemblies of einkorn broadly relevant to wheat improvement. Beyond the resources provided by their genome assemblies and gene model/TE annotations, the authors generate resequencing data for hundreds of domesticated and wild einkorn individuals and leverage these data to test hypotheses of einkorn domestication and post-domestication introgression. They also document centromere shifts and the presence of large structural rearrangements within einkorn genomes. This investigation should move the field forward and the high-quality genomics and population genomic analyses will be of interest and serve as an example for researchers working broadly across species. Below I provide suggestions the authors may wish to incorporate into their paper prior to publication.

1) In lines 82-83, the authors indicate that there is "collinearity between the two einkorn assemblies and the bread wheat A subgenome, except for chromosome 4A, which experienced multiple rearrangements in bread wheat25,26 (Supplementary Fig. 3)". It appears, based on Supp. Fig. 3, that the authors have oriented their pseudomolecule for chromosome 4A in the opposite orientation of the bread wheat A subgenome chr. 4A, but largescale rearrangements are otherwise not apparent. Other einkorn chromosomes clearly have large inversions relative to bread wheat, so it is not clear exactly how chr. 4A is distinguished. Some clarification would be helpful here.

2) Have the authors explored whether the wild alpha individual with the chr. 4A tandem duplication appears to be admixed with the wild beta population or with domesticated einkorn? There are a few alpha individuals that appear to be pulled toward the other groups in the PCA and I'm guessing these are the same individuals that show evidence of admixture in the STRUCTURE analysis. Is the alpha individual with the duplication one of these admixed individuals? If so, is there evidence of admixture outside of the chr. 4A duplication?

3) The accession used for the wild genome assembly (TA299) was from the alpha group. Based on data in Supplementary Table 9, there is a substantial drop down in the percentage of reads mapped for individuals outside the alpha group. While the authors report that population structure is not affected by choice of reference for mapping (wild vs. domesticated assembly), I am concerned that nucleotide diversity values may be more affected. I think it would also be useful to report diversity values based on mapping to TA10622. The alpha group is particularly divergent from the beta and domesticated groups, so it would be good to ensure diversity values based on mapping to an alpha genome do not contribute to, for example, the inference of a weak genetic bottleneck during domestication.

4) In lines 299-300 and 325-326, the authors suggest that introgression from the gamma group to

domesticated einkorn wheat may have restored diversity after a domestication bottleneck. The authors have identified putatively introgressed regions in domesticated individuals, so this hypothesis should be testable with their data. How does nucleotide diversity decrease when counting putatively introgressed regions as missing data?

Referee #2 (Remarks to the Author):

The manuscript presents construction of a genome for einkorn (wild and domesticated) and re-sequencing of a population diversity panel. The genomes are good quality and they are an improvement on other Triticeae assemblies currently available, however given that sequencing is becoming much easier and cheaper this isn't now such a significant achievement. There is a lot of detail about how the assembly was produced, including how the duplication was found and fixed, but this takes up a lot of space in the manuscript that could have been used to present more interesting biology. What is important is that the genome is good quality all the detail about how it was achieved is not needed in the main text. The selection of the 2 lines sequenced seems quite arbitrary, why not pick lines with relevance, rather than just representative of diversity from GBS data?

There is much made within the manuscript of how the genome can be used for the improvement of einkorn and wheat, but no information about how the genome will be used. There is no analysis of any useful gene families and given that there is re-sequencing data from >200 lines, there could have been significant analysis of traits and genes controlling these that could potentially be used. The manuscript needs this information to be of any value. The authors state that there is deep sequencing of the population, however the coverage of 10X is not deep sequencing. There is a lot of detail regarding the centromeres, however how is this useful for breeding and improvement?

The SNP data from the population - what genes were the exotic SNPs in - there could have been analysis on the gene families, which would have been of interest, especially if linked to phenotypic information, but none of this is presented. For the rare variants, are there particular lines these are in, or again any that could be biologically interesting? There is no large reduction in nucleotide diversity in the domesticated einkorn - this needs more context - when was einkorn domesticated? How long was the domestication - would there realistically be expected to be a large reduction given the breeding history? How can the similarity of diversity suggest it will be a source for bread wheat improvement, this is very throwaway and without other analyses suggested is not enough information.

Some of the figures would be more suitable for the supplementary, for example the PCA. The discussion is very short and full of hyperbole, it is not really discussing the results, but rather just repeating the main findings.

Referee #3 (Remarks to the Author):

The authors describe comprehensive genome assemblies and annotations of wild and domesticated einkorn, with the intent of interpreting how genomes adapted/evolved during einkorn domestication. The assemblies and annotations themselves are very high quality, and the paper may stand alone on these merits alone (although quality genome assemblies are becoming routine). The assemblies revealed a clear evolutionary interpretation of centromere dynamics during einkorn evolution. They find that einkorn centromeres are strikingly composed by LTR elements in contrast to most commonly found tandem repeat-based centromeres. They also found a high frequency of centromere rearrangements mostly due to inversions, suggesting that wheat centromeres are very dynamic. They nicely describe and interpret the comparative genomic data with well-prepared figures.

Some comments follow below:
A better description of the accessions selected would be nice.
There are no major rearrangements between einkorn chr4a and bread wheat. It is just the orientation of chr4a that is different and a small inversion.
I wonder if the small centromere peak observed in chr4a in TA299 einkorn is real or due to a mapping strategy limitation. I would assume that based on the mapping settings any region enriched with Cereba related will show a peak. Therefore, the sequencing and normalization using an input or mock control should be added. If the secondary peak is real and really presents an ongoing case of centromere shift this is very unique and rare to find in nature. Therefore, I ask the authors to make sure about their findings and interpretation. This is an excellent finding. I would also expect a differential methylation status of the two centromere peaks in this chromosome. Ideally, immunostaining with CENH3 antibody could easily validate that particular centromere structure. However, I understand such experiments might not be feasible depending on the lab's expertise.
The 1mb tandem duplication is present in domesticated bread wheat?
How many centromeres were completely gap free assembled? Were centromeric repeats found in unplaced contigs? This could suggest potential missing sequences at centromeres.
Is there a difference at sequence level among Cereba elements within and out of centromeres besides the age of the element? DNA methylation levels differ? It might be interesting to check the methylation level of functional centromeres compared to neighboring regions and old Cereba elements.
ChIPseq analysis seems very naïve as there was no input or mock control included in the analysis. Although I believe the ChIP-seq worked, a sequencing of either the input chromatin or a mock control like IgG should be included to reduce artifacts while mapping to highly repetitive regions, i.e., centromeres. Also, I would recommend all ChIPseq experiments to be performed using at least 2 replicates. Furthermore, ChIP bam files should be normalized using tools like bamCoverage or bamCompare from deeptools. Metaplots of CENH3 enrichment over Cereba repeats inside and outside centromeres could also be performed. The boundaries of centromere peaks could be facilitated by calling peaks with the epic2 (https://github.com/biocore-ntnu/epic2) peak caller, which is specially designed for regional centromeres.
Mapping strategy should allow only the best match.
Although the study describes a good characterization mapping-based of centromeres, I miss a de novo approach to identify potential new centromeric repeats. This could be, for instance, done with the ChIP-seq mapper tool available on RepeatExplorer (https://repeatexplorer-elixir.cerit-sc.cz/galaxy). Alternatively, the authors could come up with some script to detect the chip enrichment ratio by mapping Cenh3 and input reads to individual consensus sequences all main repeats identified by TRF and EDTA.
There are some regions with clear lower Cenh3 signal inside centromeres. What are those regions? Are they depleted of Cereba repeats? Do genes in centromeres show any enrichment with CENH3 chromatin?
This study is interesting primarily because of the unique centromere structures of einkorn. The findings that centromere sequences differ between cultivated and wild einkorn is interesting. It is unclear what sequence features if any Cereba has that facilitate CENH3 deposition. Also, it would have been nice to detect whether a high order structure of Cereba elements within functional centromeres exist.
So, the paper is primarily an excellent comparative genome analysis of different wild and cultivated einkorn accessions. The centromere analysis included in the study is very interesting and helps to understand the evolution of einkorn genomes. The paper would have been more complete had they carried ChIP-seq using more controls and other histone marks as well to get a more wholistic perspective of the overall genome organization and regulation of LTR-based centromeres in einkorn.

Author Rebuttals to Initial Comments:

Point-by-point responses (Please note that line numbers indicated in the point-by-point responses can vary depending on the operating system on which the word document is opened. The line numbers indicated in the point-by-point responses are based on Windows operating system, letter page size with normal margins and the 'All Markup' view).

Referee #1 (Remarks to the Author):

In their manuscript, Ahmed and colleagues describe their high-quality genome assemblies of wild and domesticated einkorn wheat. These assemblies are state-of-the-art, showing high levels of contiguity and completeness. This is a substantial step forward for wheat genomics as previous efforts toward genome assembly have been challenged by the exceptional abundance of repetitive sequences and the large genome sizes of the species. While einkorn wheat is domesticated itself, it is also closely related to a genome donor to both tetraploid and hexaploid domesticated wheat species, making assemblies of einkorn broadly relevant to wheat improvement. Beyond the resources provided by their genome assemblies and gene model/TE annotations, the authors generate resequencing data for hundreds of domesticated and wild einkorn individuals and leverage these data to test hypotheses of einkorn domestication and post-domestication introgression. They also document centromere shifts and the presence of large structural rearrangements within einkorn genomes. This investigation should move the field forward and the high-quality genomics and population genomic analyses will be of interest and serve as an example for researchers working broadly across species. Below I provide suggestions the authors may wish to incorporate into their paper prior to publication.

<u>Comment 1.1:</u> In lines 82-83, the authors indicate that there is "collinearity between the two einkorn assemblies and the bread wheat A subgenome, except for chromosome 4A, which experienced multiple rearrangements in bread wheat25,26 (Supplementary Fig. 3)". It appears, based on Supp. Fig. 3, that the authors have oriented their pseudomolecule for chromosome 4A in the opposite orientation of the bread wheat A subgenome chr. 4A, but largescale rearrangements are otherwise not apparent. Other einkorn chromosomes clearly have large inversions relative to bread wheat, so it is not clear exactly how chr. 4A is distinguished. Some clarification would be helpful here.
<u>Our response:</u>
The chromosome 4A rearrangements in polyploid wheat are well documented and described, but most likely largely unknown to the non-wheat community. These rearrangements include pericentric inversions ("swap" of long and short arms) and a translocation from chromosome 7BS (The 7BS translocation is visible as a "missing" segment in the dot plot in Supplementary Fig. 3 because we only compare against the bread wheat A subgenome). For clarification, we have modified our statement as follows (lines 90-94): 'Similarly, we observed a high degree of collinearity between the two einkorn assemblies and the bread wheat A subgenome. The most obvious exceptions were the well-described rearrangements of chromosome 4A, which include pericentric inversions and translocations in polyploid wheat (Supplementary Fig. 3).' We now cite a reference that specifically focuses on the chromosome 4A rearrangements. Our orientation of the monococcum chromosome 4A is thus correct because this chromosome was not affected by the pericentric inversions.

Comment 1.2: Have the authors explored whether the wild alpha individual with the chr. 4A tandem duplication appears to be admixed with the wild beta population or with domesticated einkorn? There are a few alpha individuals that appear to be pulled toward the other groups in the PCA and I'm guessing these are the same individuals that show evidence of admixture in the STRUCTURE analysis. Is the alpha individual with the duplication one of these admixed individuals? If so, is there evidence of admixture outside of the chr. 4A duplication?
Our response:
Alpha accession TA316, which carries the duplication based on read coverage, shows no admixture based on the PCA and STRUCTURE plot. TA316 is thus not among the alpha individuals that show putative admixture in the STRUCTURE analysis. We cannot exclude, however, that this accession carries a small introgression from a domesticated or beta accession that includes the duplication.

Comment 1.3: The accession used for the wild genome assembly (TA299) was from the alpha group. Based on data in Supplementary Table 9, there is a substantial drop down in the percentage of reads mapped for individuals outside the alpha group. While the authors report that population structure is not affected by choice of reference for mapping (wild vs. domesticated assembly), I am concerned that nucleotide diversity values may be more affected. I think it would also be useful to report diversity values based on mapping to TA10622. The alpha group is particularly divergent from the beta and domesticated groups, so it would be good to ensure diversity values based on mapping to an alpha genome do not contribute to, for example, the inference of a weak genetic bottleneck during domestication.
Our response:
We have now also calculated the nucleotide diversity based on the mapping to TA10622 and obtained very similar values compared to the read mapping against TA299. The values based on TA10622 are now included in the main manuscript as follows (lines 287-291): 'Variant calling using the TA10622 assembly revealed very similar results via population divergence, PCA, and nucleotide diversity (α; π=0.0012, β; π=0.0017, γ; π=0.0022, domesticated; π=0.0012) (Supplementary Fig. 19 a-c), confirming the high accuracy of variant calling and the independence of population structure analyses from which reference assembly is used.' We included the new supplementary figure 19c to show the nucleotide diversity based on the read mapping against TA10622.

Comment 1.4: In lines 299-300 and 325-326, the authors suggest that introgression from the gamma group to domesticated einkorn wheat may have restored diversity after a domestication bottleneck. The authors have identified putatively introgressed regions in domesticated individuals, so this hypothesis should be testable with their data. How does nucleotide diversity decrease when counting putatively introgressed regions as missing data?
Our response:
The average nucleotide diversity in domesticated einkorn decreased from 0.0014 to 0.0012 when excluding the genomic segments with putative γ introgressions. The average nucleotide diversity was 0.0019 in the genomic segments with putative γ introgressions. This data is now shown in the new Supplementary Fig. 25c and referred to in the main text in lines 322-325: 'Overall, we estimated that the introgressions from race γ accounted for an average of 6.7% (range 0.3–13.1%) of the domesticated einkorn genome (Supplementary Table 12), resulting in an increased nucleotide diversity within the domesticated gene pool (Supplementary Fig. 25 a-c).'

Referee #2 (Remarks to the Author):

Comment 2.1: The manuscript presents construction of a genome for einkorn (wild and domesticated) and re-sequencing of a population diversity panel. The genomes are good quality and they are an improvement on other Triticeae assemblies currently available, however given that sequencing is becoming much easier and cheaper this isn't now such a significant achievement.
Our response:
Sequencing wheat genomes has represented an enormous challenge in the past. Although sequencing has become cheaper and easier, assembling a 5.1-Gb highly repetitive wheat genome to this quality is still a significant achievement in our view. Once published, the two einkorn assemblies will be the most complete and most contiguous Triticeae assemblies in the public domain.

Comment 2.2: There is a lot of detail about how the assembly was produced, including how the duplication was found and fixed, but this takes up a lot of space in the manuscript that could have been used to present more interesting biology. What is important is that the genome is good quality all the detail about how it was achieved is not needed in the main text.
Our response:
This is a valid point. The assembly statistics are summarized in table 1 and we have therefore shortened the corresponding description in the main text. Also, we shortened the description on how the tandem duplication was identified and moved the corresponding text to the methods section.

Comment 2.3: The selection of the 2 lines sequenced seems quite arbitrary, why not pick lines with relevance, rather than just representative of diversity from GBS data?
Our response:
We do not think that the selection of the two accessions was arbitrary. Einkorn is unique among wheat because it has not experienced the same level of breeding and improvement as durum and bread wheat. In contrast to elite durum and bread wheat cultivars, it is thus not possible to judge the 'relevance' of an einkorn accession based on its breeding history or use in agriculture. Our aim here was to produce genomic resources of "typical" representatives of the wild alpha and the domesticated einkorn gene pool. This is why we based our selection on a diversity study using GBS data to avoid selection of admixed lines for sequencing.

Comment 2.4: There is much made within the manuscript of how the genome can be used for the improvement of einkorn and wheat, but no information about how the genome will be used. There is no analysis of any useful gene families and given that there is re-sequencing data from >200 lines, there could have been significant analysis of traits and genes controlling these that could potentially be used. The manuscript needs this information to be of any value.
Our response:
We have added a new paragraph that demonstrates how the assemblies can be used for gene cloning and wheat improvement. The new paragraph describes the genomics-assisted cloning of the tiller inhibition (tin3) gene. Tillering is a key plant architecture trait in cereals that is related to spike number and grain yield. tin3 is a recessive mutant that shows significantly reduced tillering. Using a MutMap-based approach, we show that tin3 encodes a putative co-transcription factor. Based on the knowledge gained in einkorn, we produced a mutant line in hexaploid bread wheat that carries point mutations in all three tin3 homoeologs. The tipple bread wheat mutant

showed a significant reduction in tiller number, while plants having mutations in only one or two tin3 homoeologs showed normal tillering. The genomics-assisted cloning of tin3 demonstrates the usefulness of our genomic resources for gene cloning and provides an example of how knowledge gained from diploid einkorn wheat can be translated to polyploid bread wheat. The cloning of tin3 is summarized in the new paragraph 'Mapping of a plant architecture gene' (lines 391-414) and in the new main figure 5.

Comment 2.5: The authors state that there is deep sequencing of the population, however the coverage of 10X is not deep sequencing.
Our response:
We have replaced the term 'deep whole-genome sequencing' with 'whole-genome sequencing' throughout the manuscript.

Comment 2.6: There is a lot of detail regarding the centromeres, however how is this useful for breeding and improvement?
Our response:
The centromere part describes fundamental new discoveries in genome biology (see also comments by reviewer 3). The architecture, evolution and dynamics of plant centromeres are only poorly understood because of the difficulties in assembling gapless centromeres. This is particularly true for Triticeae species, for which complete centromeres have not been assembled so far. Here, we assembled complete wheat centromeres for the first time, which gave us unprecedented insight into basic centromere biology. The discovery of centromere shifts and the underlying mechanisms (inversions as drivers of centromere shifts) is of high novelty. The analysis of complete centromere structures in the model plant Arabidopsis, as a comparison, was published in Science (Naish et al. (2021) Science 374, 840). The understanding of basic centromere structures is of relevance for synthetic biology, for example for chromosome restructuring and engineering of small chromosomes that carry gene stacks (Dawe et al. (2023) Nature Plants 9: 433-441, Rönspies et al. (2022) Nature Plants 8:1153-1159).

Comment 2.7: The SNP data from the population - what genes were the exotic SNPs in - there could have been analysis on the gene families, which would have been of interest, especially if linked to phenotypic information, but none of this is presented.
Our response:
In total, we identified 317,023 non-synonymous exonic SNPs. These SNPs affected 26,505 high-confidence genes (=82% of all gene models). We do not think that a gene family analysis would have been insightful given the high proportion of genes with exonic SNPs. The number of genes affected by non-synonymous exonic SNPs is now indicated in the main text as follows (lines 266-268): 'Of the exonic SNPs, 317,023 (53.4%) were non-synonymous affecting 26,505 genes, of which 9,145 SNPs resulted in a disruption of coding sequences (premature stop codon) in 5,726 genes.'

Comment 2.8: For the rare variants, are there particular lines these are in, or again any that could be biologically interesting?
Our response:
We have added the percentages of rare variants for each accession to Supplementary Table 9. There was no accession that stood out with a particularly high percentage of rare variants.

Comment 2.9: There is no large reduction in nucleotide diversity in the domesticated einkorn - this needs more context - when was einkorn domesticated? How long was the domestication - would there realistically be expected to be a large reduction given the breeding history? How can the similarity of diversity suggest it will be a source for bread wheat improvement, this is very throwaway and without other analyses suggested is not enough information.

Our response:

This is a good suggestion. We have expanded our explanation on the possible role of the gamma introgressions into domesticated einkorn as follows (lines 350 – 361): 'The apparent lack of a strong domestication bottleneck in domesticated einkorn was explained with a 'dispersed-specific' model of einkorn domestication, including multiple domestication events from geographically dispersed wild β populations[13]. A hallmark of domesticated einkorn is the non-fragile rachis. In our diversity panel, all domesticated einkorn accessions had the same haplotype in the non-brittle rachis1 (btr1) gene, including a critical alanine to threonine amino acid substitution[4], indicating that this key domestication gene has a single origin in domesticated einkorn. The lack of a strong diversity reduction in domesticated einkorn could thus also be the result of gene flow following domestication, as demonstrated for the introgressions from wild γ accessions. Recent population and pan-genome analyses confirmed that hybridizations played an important role in increasing genetic diversity in wheat after domestication[23,51]. The introgression of genetic material from wild γ accessions may have played an important role in the adaptation of domesticated einkorn to new climatic conditions outside the Fertile Crescent.'. As suggested by reviewer 1 (comment 1.4), we have now integrated the new Supplementary Fig. 25c, demonstrating that the gamma introgressions resulted in increased nucleotide diversity (lines 322-325): 'Overall, we estimated that the introgressions from race γ accounted for an average of 6.7% (range 0.3–13.1%) of the domesticated einkorn genome (Supplementary Table 12), resulting in an increased nucleotide diversity within the domesticated gene pool (Supplementary Fig. 25 a-c).'. We have also clarified why einkorn can be a valuable source of genetic diversity for bread wheat improvement (lines 55-58): 'In contrast to T. urartu, wild and domesticated einkorn have a long history of cultivation and human selection in diverse environmental conditions, which makes einkorn a valuable source of genetic variation for wheat breeding.'

Comment 2.10: Some of the figures would be more suitable for the supplementary, for example the PCA. The discussion is very short and full of hyperbole, it is not really discussing the results, but rather just repeating the main findings.

Our response:

As suggested, the PCA from main Fig. 3a has now been moved to Supplementary Fig. 17e. We agree with the reviewer that the discussion was mainly repeating the main findings. In the revised version, we have removed the final discussion paragraph. Instead, we discuss the different main findings in the respective paragraphs.

Referee #3 (Remarks to the Author):

The authors describe comprehensive genome assemblies and annotations of wild and domesticated einkorn, with the intent of interpreting how genomes adapted/evolved during einkorn domestication. The assemblies and annotations themselves are very high quality, and the paper may stand alone on these merits alone (although quality genome assemblies are becoming routine). The assemblies revealed a clear evolutionary interpretation of centromere dynamics during einkorn evolution. They find that einkorn centromeres are strikingly composed by LTR elements in contrast to most commonly found tandem repeat-based centromeres. They also found a high frequency of centromere rearrangements mostly due to inversions, suggesting that wheat centromeres are very dynamic. They nicely describe and interpret the comparative genomic data with well-prepared figures. Some comments follow below:

<u>Comment 3.1:</u> A better description of the accessions selected would be nice.
<u>*Our response:*</u>
We provide additional information about TA10622 and TA299 as follows (lines 78-81): 'TA10622 is a domesticated einkorn landrace (T. monococcum L. subsp. monococcum) with non-brittle rachis that was collected in Albania at the beginning of the 20th century. Wild einkorn accession TA299 (T. monococcum L. subsp. aegilopoides; race α) was collected during an expedition in 1972 in northern Iraq[21] and has a brittle rachis.' We also state in the introduction that rachis brittleness is the most obvious morphological difference between wild and domesticated einkorn (lines 51-54)" 'A noticable morphological difference between wild and domesticated einkorn is the grain dispersal system. Wild einkorn have a fragile rachis, which facilitates seed dispersal, while the rachis in domesticated einkorn accessions is non-brittle.' Also, see our response to reviewer 2's comment (comment 2.3) regarding the relevance of the selected accessions. Einkorn is unique among wheat because it has not experienced the same level of breeding and improvement as durum and bread wheat. In contrast to elite durum and bread wheat cultivars, it is thus not possible to judge the 'relevance' of an einkorn accession based on its breeding history or use in agriculture. Our aim here was to produce genomic resources of "typical" representatives of the wild alpha and the domesticated einkorn gene pool. This is why we based our selection on a diversity study using GBS data to avoid selection of admixed lines for sequencing.

<u>Comment 3.2:</u> There are no major rearrangements between einkorn chr4a and bread wheat. It is just the orientation of chr4a that is different and a small inversion.
<u>*Our response (see also comment 1.1 by reviewer 1):*</u>
The chromosome 4A rearrangements in polyploid wheat are well documented and described, but most likely largely unknown to the non-wheat community. These rearrangements include pericentric inversions ("swap" of long and short arms) and a translocation from chromosome 7BS (The 7BS translocation is visible as a "missing" segment in the dot plot in Supplementary Fig. 3 because we only compare against the bread wheat A subgenome). For clarification, we have modified our statement as follows (lines 90-94): 'Similarly, we observed a high degree of collinearity between the two einkorn assemblies and the bread wheat A subgenome. The most obvious exceptions were the well-described rearrangements of chromosome 4A, which include pericentric inversions and translocations in polyploid wheat (Supplementary Fig. 3).' We now cite a reference that specifically focuses on the chromosome 4A rearrangements. Our orientation of

the monococcum chromosome 4A is thus correct because this chromosome was not affected by the pericentric inversions.

<u>Comment 3.3:</u> I wonder if the small centromere peak observed in chr4a in TA299 einkorn is real or due to a mapping strategy limitation.

<u>Our response:</u>

Based on the reviewer's comment, we revised the analysis using different filtering criteria (see also response to comment 3.5). Namely, we only allowed primary alignments (i.e., discarding multi-mapping reads). Additionally, alignments were filtered for mapping quality (MAPQ ≥ 30), only retaining high-quality alignments and removing ambiguously mapped reads. We discarded multi-mapping reads because alignments with two identical possible mapping positions are assigned randomly, which can lead to CENH3 enrichment being wrongly suggested. After applying this filtering strategy, the secondary CENH3 peak in chromosome 4A is indeed not observable anymore. This is in line with findings that dicentric chromosomes are not stable (Henderson et al. (2023) Nature Plants 9: 379-380). The plots in the main Figure 2b and the respective supplementary figures (Supplementary Figs. 5, 6, 7, 9, 13, 14, and 15) were revised accordingly. The methods section was updated with information on the new data sets and filtering criteria (Lines 778-798). The new, filtered CENH3 ChIP-seq data was used for all analyses throughout the manuscript. Please note that the "secondary" peak was not considered as being part of the functional centromere in our original manuscript version (original Supplementary Fig. 6).

<u>Comment 3.4:</u> I would assume that based on the mapping settings any region enriched with Cereba related will show a peak.

<u>Our response:</u>

The reviewer is correct. As described in the previous response (3.3), after applying more stringent mapping criteria the RLG_Cereba elements outside the functional centromeres do not show any CENH3 enrichment. Our mapping can therefore distinguish between regions enriched in RLG_Cereba elements outside centromeres, and regions that are part of the functional centromere. This can for example be seen in Fig. 2a, where one can see an enrichment of RLG_Cereba elements approximately at position 328 Mb on chromosome 3A that shows now elevated CENH3 coverage.

Comment 3.5: Therefore, the sequencing and normalization using an input or mock control should be added.

<u>Our response:</u>

To address this valid comment, we have performed new ChIP-Seq experiments using input controls. The resulting ChIP-Seq mappings were then normalized against the respective input control using the deepTools function bamCompare. The ChIP-Seq and control experiments were performed in two independent replicates. The new, filtered CENH3 ChIP-Seq data was used for all analyses throughout the manuscript and the respective figures and supplementary figures were revised using the new datasets. (See also our response to comments 3.3 and 3.13). While we believe that the new data greatly increases the robustness of our analyses, all of our major conclusions made in the initial manuscript version could be confirmed, indicating that the first ChIP-Seq experiment worked.

Comment 3.6: If the secondary peak is real and really presents an ongoing case of centromere shift this is very unique and rare to find in nature. Therefore, I ask the authors to make sure about their findings and interpretation. This is an excellent finding.

Our response:

As indicated in our response to comment 3.3, the weak CENH3 signal disappeared with more stringent mapping criteria. The peak was not considered to be part of the functional centromere in our initial manuscript version.

Comment 3.7: I would also expect a differential methylation status of the two centromere peaks in this chromosome. Ideally, immunostaining with CENH3 antibody could easily validate that particular centromere structure. However, I understand such experiments might not be feasible depending on the lab's expertise.

Our response:

To address the reviewer's comment, we determined CpG DNA methylation using the ccsmeth package(https://github.com/PengNi/ccsmeth) for accession TA299. To test whether there is a differential methylation status between the old and the new centromere of chromosome 4A of accession TA299, we calculated the mean methylation frequency in 100 kb windows along each region. A Mann-Whitney U test between the two groups revealed no significant difference between the two regions (p-value = 0.6959). Based on our responses to comments 3.3 and 3.6 we did not include this information in the revised manuscript.

Comment 3.8: The 1mb tandem duplication is present in domesticated bread wheat?

Our response:

No, the 1-Mb duplication is specific to einkorn and was not found in bread wheat. We have included this statement in the revised version in the main text as follows (lines 132-133): 'The tandem duplication was specific to einkorn and was not found in bread wheat.'

Comment 3.9: How many centromeres were completely gap free assembled?

Our response:

Out of the 14 centromeres (TA299 and TA10622), 13 were assembled gap-free and validated by the optical map. We emphasize this point more clearly in the revised manuscript as follows (lines 167-169): 'Crucial for our analysis was that centromeric regions in both accessions were assembled contiguously without sequence gaps (Supplementary Fig. 9) and validated by optical map data. The only exception was the chromosome 2A centromere of TA10622, which carried two small gaps.'

Comment 3.10: Were centromeric repeats found in unplaced contigs? This could suggest potential missing sequences at centromeres.

Our response:

TA299, in which all the centromeres were assembled gap-free, only has one RLG_Cereba element in the unplaced contigs, with an estimated insertion age of 0.6 million years. There is also no enrichment of CENH3 in the unplaced contigs of the TA299 assembly (Supplementary Fig. 5). By contrast, 17 RLG_Cereba and 7 RLG_Quinta elements, as well as some enrichment of CENH3 were found in the unplaced contigs of the TA10622 assembly (Supplementary Fig. 5). We have evidence that these sequences correspond to the two gaps in the assembly of the chromosome 2A centromere. Even in TA10622, the number of RLG_Cereba and RLG_Quinta elements in the unplaced contigs is very low compared to the total number of RLG_Cereba and RLG_Quinta

elements in the einkorn genome (corresponding to 0.7% of RLG_Cereba and RLG_Quinta full-length elements, respectively), demonstrating the very high quality of our centromere assemblies. By comparison, in the Chinese Spring bread wheat reference assembly (IWGSC (2018) Science 361:eaar6089), ~25% of the centromere sequences were estimated to be located on the unplaced contigs.

Comment 3.11: Is there a difference at sequence level among Cereba elements within and out of centromeres besides the age of the element?

Our response:

To address this question, we used the recently published TEpop pipeline, which analyzes populations of full-length LTR retrotransposons (Wicker et al. (2022) Advanced Genetics 3, 2100000; https://doi.org/10.1002/ggn2.202100022). Here, individual full-length RLG_Cereba elements are aligned to a reference (a RLG_Cereba consensus sequence). This allows the identification of polymorphic sites, which can then be used for principal component analysis (PCA) of RLG_Cereba populations. With this approach, we identified three main sub-populations of RLG_Cereba elements, hereafter referred to as alpha, beta and gamma. The gamma population contains the youngest (i.e., the most recently inserted) retrotransposon copies. The majority of RLG_Cereba elements located in the centromeres are part of this gamma sub-population (92%). In total, ~63% of the RLG_Cereba elements belonging to the gamma sub-population are found in the functional centromeres. This is in contrast to the alpha and beta sub-populations, whose members are predominantly found outside of the functional centromeres (0.3% and 13.4% inside functional centromeres, respectively). This reflects the fact that RLG_Cereba elements target centromeres for insertions, which results in older elements (i.e., alpha and beta sub-populations) being "pushed" outside of the centromeres over time. This detailed information is now provided in the new Supplementary Fig. 12 and referred to briefly in the main text (lines 195-196): The evolutionary youngest TE sub-population is thus almost exclusively found in functional centromeres (Supplementary Fig. 12 and 13).' However, we did not detect a specific sequence difference that would distinguish the gamma RLG_Cereba elements inside and outside functional centromeres.

Comment 3.12: DNA methylation levels differ? It might be interesting to check the methylation level of functional centromeres compared to neighboring regions and old Cereba elements.

Our response: As decribed in response to comment 3.7, CpG DNA methylation data was inferred from PacBio reads. In addition, we performed a new ChIP-Seq experiment targeting H3K4me3 histone modification. This analysis revealed that centromeres indeed have lower levels of CpG methylation compared to the surrounding regions. This is now mentioned in the main text (line 165-167) 'Einkorn centromeres are also local minima of CpG methylation and H3K4me3 histone modification (Supplementary Figs. 7 and 8).' and shown in the new Supplementary Figure 7. The overall lower methylation levels of centromeric regions are in part explained by low methylation levels in the LTRs of RLG_Cereba elements. However, we did not find differences in methylation levels between RLG_Cereba elements from inside and outside functional centromeres, or between young and old RLG_Cereba copies. This is now shown in the new Supplementary Figure 8. The methods were updated accordingly (Lines 748-798).

Comment 3.13: ChIPseq analysis seems very naïve as there was no input or mock control included in the analysis. Although I believe the ChIP-seq worked, a sequencing of either the input chromatin or a mock control like IgG should be included to reduce artifacts while mapping to highly repetitive

regions, i.e., centromeres. Also, I would recommend all ChIPseq experiments to be performed using at least 2 replicates. Furthermore, ChIP bam files should be normalized using tools like bamCoverage or bamCompare from deeptools.
Our response:
The ChIP-Seq experiments were repeated, including an input control against which the ChIP-Seq mappings were normalized. The new data was used consistently for all analyses (see also our response to comment 3.5 above for details).

Comment 3.14: Metaplots of CENH3 enrichment over Cereba repeats inside and outside centromeres could also be performed.
Our response:
Metaplots of RLG_Cereba elements show that there is a clear enrichment of CENH3 in centromeric RLG_Cereba elements, whereas the CENH3 levels of non-centromeric RLG_Cereba elements is indistinguishable from their surrounding sequences, further confirming that the revised mapping strategy allows for differentiation between regions enriched in RLG_Cereba elements and regions that are indeed part of the functional centromeres. However, our mapping strategy removes ambiguous reads (i.e., reads mapping to conserved regions in RLG_Cereba elements). This results in the metaplots showing lower CENH3 levels inside RLG_Cereba elements compared to the areas surrounding the elements (a bias resulting from the exclusion of multi-mapping and reflecting the high sequence conservation). We see this data as mostly confirmatory and therefore would prefer to not show it, also in order to not inflate the number of supplementary figures unnecessarily.

Comment 3.15: The boundaries of centromere peaks could be facilitated by calling peaks with the epic2 (https://github.com/biocore-ntnu/epic2) peak caller, which is specially designed for regional centromeres.
Our response:
As suggested, we used epic2 to identify CENH3 peaks for each CENH3 ChIP-Seq replicate using the respective input control. This is now reflected in all new main and supplementary figures showing centromere peaks. The methods were updates accordingly (Line 748-798).

Comment 3.16: Mapping strategy should allow only the best match.
Our response:
Based on the reviewer's comments, our mapping strategy was adjusted to only allow for the best matching alignment. (See also responses to comments 3.3 and 3.6).

Comment 3.17: Although the study describes a good characterization mapping-based of centromeres, I miss a de novo approach to identify potential new centromeric repeats. This could be, for instance, done with the ChIP-seq mapper tool available on RepeatExplorer (https://repeatexplorer-elixir.cerit-sc.cz/galaxy). Alternatively, the authors could come up with some script to detect the chip enrichment ratio by mapping Cenh3 and input reads to individual consensus sequences all main repeats identified by TRF and EDTA.
Our response:
As suggested by the reviewer, we used the ChIP-Seq mapper tool as an independent approach to complement our homology and annotation-based repeat analysis. The software identified only 2 repeat clusters that passed the threshold of >5-fold enrichment. Both sequence clusters were derived from RLG_Cereba or RLG_Quinta elements, and thus did not identify any additional

repeat sequences. We have added this information to the methods section as follows (lines 835-848): 'To complement our homology and annotation-based repeat analysis, we used the ChIP-Seq mapper tool, which is part of the RepeatExplorer2 software collection. First, repeats were identified by clustering short sequencing reads with RepeatExplorer2, which does not depend on the reference genome and would therefore also identify repetitive elements missing from the reference. For this, ~20x coverage Illumina short reads were down-sampled to the recommended coverage equivalent of 0.5x using seqkit (https://github.com/shenwei356/seqkit). CENH3-ChIP and control reads were then mapped onto the identified repeat clusters using the ChIP-Seq mapper tool. Two repeat clusters passed the threshold of >5-fold enrichment. The unique sequences contained in these two clusters were then queried using BLASTN searches against the nrTREP20 repeat database. In one of the clusters, all 38 sequences showed very high homology with either RLG_Cereba or RLG_Quinta consensus sequences, whereas in the other cluster 37 out of 42 sequences were RLG_Cereba or RLG_Quinta sequences. Thus, ChIP-Seq mapper did not identify any additional repeat clusters enriched in CENH3 that were not found in the homology and annotation-based repeat analysis.'

Comment 3.18: There are some regions with clear lower Cenh3 signal inside centromeres. What are those regions? Are they depleted of Cereba repeats? Do genes in centromeres show any enrichment with CENH3 chromatin?
<u>Our response:</u>
The regions low in CENH3 inside the functional centromeres largely coincide with the presence of genes and therefore show lower density of RLG_Cereba repeats. Inside the centromeres, genes that are actively expressed (as measured by RNA-seq) are not enriched in CENH3, whereas genes that are not actively expressed tend to show enrichment with CENH3. The findings can be seen in Supplementary Figure 10. We added two new panels to this supplementary figure, showing that expressed and non-expressed genes differ in their methylation levels.

<u>Comment 3.19:</u> This study is interesting primarily because of the unique centromere structures of einkorn. The findings that centromere sequences differ between cultivated and wild einkorn is interesting. It is unclear what sequence features if any Cereba has that facilitate CENH3 deposition. Also, it would have been nice to detect whether a high order structure of Cereba elements within functional centromeres exist.
<u>Our response:</u>
Based on the reviewer's comment, we additionally searched for instances of tandemly repeated Cereba elements (e.g. Cereba elements with three long terminal repeats and two internal domains that resulted from unequal recombination between LTRs) using BLASTN queries against the TA299 genome assembly. In this way, 61 instances of tandemly repeated Cereba elements were identified. As a control, we also searched for such recombinant RLC_Angela elements, which are ~10 times more abundant than RLG_Cereba elements, but largely absent from centromeres. This revealed 620 tandemly repeated RLC_Angela elements. From this analysis we conclude that while there are tandemly repeated RLG_Cereba elements, the number is in the range of what could be expected from other non-centromeric TEs. This also matches our results from the tandem repeats finder software where we did not identify high-copy tandem repeat arrays in centromeres. Since these additional results mainly confirmed the existing analysis, we now provide the information in the methods section as follows (line 801-810): 'To complement the analysis with tandem repeats finder, we also searched for instances of tandemly repeated RLG_Cereba elements (e.g. RLG_Cereba elements with three long terminal repeats and two internal domains, which resulted

from unequal recombination between LTRs) using BLASTN queries against the TA299 genome assembly. In this way, 61 instances of tandemly repeated RLG_Cereba elements were identified. As a control, we also searched for such recombinant RLC_Angela elements, which are ~10 times more abundant than RLG_Cereba elements, but largely absent from functional centromeres. This revealed 620 tandemly repeated RLC_Angela elements. From this analysis, we conclude, that while there are tandemly repeated RLG_Cereba elements, the number is in the range of what could be expected from other non-centromeric TEs, revealing no higher order structure of RLG_Cereba elements.'

Comment 3.20: So, the paper is primarily an excellent comparative genome analysis of different wild and cultivated einkorn accessions. The centromere analysis included in the study is very interesting and helps to understand the evolution of einkorn genomes. The paper would have been more complete had they carried ChIP-seq using more controls and other histone marks as well to get a more wholistic perspective of the overall genome organization and regulation of LTR-based centromeres in einkorn.

Our response:

We thank the reviewer for appreciating the value of our centromere analyses. As detailed in our responses above, we have strengthened the centromere analyses using additional controls and other histone marks / methylation analyses. This also allowed us to get a more complete picture of centromere structures in the context of the overall genome organization (see the new Supplementary Figure 7c, d.).

Reviewer Reports on the First Revision:

Referees' comments:

Referee #1 (Remarks to the Author):

I appreciate the authors' efforts to address my concerns and to explore, with additional analyses, some of the possibilities I had suggested based on their previous results. As I mentioned previously, this study is a substantial step forward for wheat genomics and the analysis is thorough and impactful. I support publication.

Referee #2 (Remarks to the Author):

They didn't really address my comment:

There is no analysis of any useful gene families and given that there is re-sequencing data from >200 lines, there could have been significant analysis of traits and genes controlling these that could potentially be used. The manuscript needs this information to be of any value.

Or

For the rare variants, are there particular lines these are in, or again any that could be biologically interesting?

These should be addressed for the manuscript to be suitable for publication

The real focus of this paper is the centromere variation, and while I acknowledge it is difficult to sequence centromeres, this in itself doesn't make them of interest to a broad readership. As the authors don't seem to want to add information on variability of genes of agronomic interest, and consider such analysis as not providing insights, I personally think the paper in its current form is mostly of interest to more specialised researchers. Perhaps the title should be modified to make it clear that the focus is on centromere analysis?

Referee #3 (Remarks to the Author):

I thank the authors for all the revisions made regarding the centromere analysis. In my opinion, this has largely strengthened the study. They have added new information about control and replicate ChIP experiments in their supplementary files, that support their findings.
The results and discussion have been improved based on the added data regarding the centromere structure and evolution in Einkorn.

I still did not get the pericentric inversion on Chr4a, as I did not see in the dotplots any region in the same orientation between bread wheat and einkorn. I would suggest the authors show the synteny in a SyRI (plotsr) plot (https://github.com/schneebergerlab/plotsr), which will make the comparison between the two genomes much clear that just a simple dotplot.

I would be happy to recommend the manuscript for publication.

Author Rebuttals to First Revision:

Point-by-point responses

Referee #1 (Remarks to the Author): I appreciate the authors' efforts to address my concerns and to explore, with additional analyses, some of the possibilities I had suggested based on their previous results. As I mentioned previously, this study is a substantial step forward for wheat genomics and the analysis is thorough and impactful. I support publication.

> Our response: We thank this reviewer for the constructive comments, which helped to significantly improve our manuscript.

Referee #2 (Remarks to the Author): They didn’t really address my comment: There is no analysis of any useful gene families and given that there is re-sequencing data from>200 lines, there could have been significant analysis of traits and genes controlling these that could potentially be used. The manuscript needs this information to be of any value.

> Our response: As described in the first version of our rebuttal letter, we have included a new paragraph highlighting the value of our einkorn genomic resources to clone useful genes. We demonstrated how this knowledge can be transferred to bread wheat.

Or, for the rare variants, are there particular lines these are in, or again any that could be biologically interesting? These should be addressed for the manuscript to be suitable for publication.

> Our response: We have added the percentages of rare variants for each accession in the Supplementary Table 9 (now Supplementary Table 15). As described in our first version of the rebuttal letter, there was no particular einkorn accession with a particularly high percentage of rare variants, indicating that the rare variants are distributed across all einkorn accessions. Please note that we found over 55 million rare variants and we do not think that there is a straightforward way to determine if these are biologically interesting.

The real focus of this paper is the centromere variation, and while I acknowledge it is difficult to sequence centromeres, this in itself doesn’t make them of interest to a broad readership. As the authors don’t seem to want to add information on variability of genes of agronomic interest, and consider such analysis as not providing insights, I personally think the paper in its current form is mostly of interest to more specialised researchers. Perhaps the title should be modified to make it clear that the focus is on centromere analysis?

> Our response: We agree that the centromere analysis represents a substantial part of our manuscript. As indicated by reviewer 3, the analysis of complete wheat centromeres if of high novelty and of interest to a broad readership.

Referee #3 (Remarks to the Author): I thank the authors for all the revisions made regarding the centromere analysis. In my opinion, this has largely strengthened the study. They have added new information about control and replicate ChIP experiments in their supplementary files, that support their findings. The results and discussion have been improved based on the added data regarding the centromere structure and evolution in Einkorn. I still did not get the pericentric inversion on Chr4a, as I did not see in the dotplots any region in the same orientation between bread wheat and einkorn. I would suggest the authors show the synteny in a SyRI (plotsr) plot

(https://github.com/schneebergerlab/plotsr), which will make the comparison between the two genomes much clear that just a simple dotplot. I would be happy to recommend the manuscript for publication.

Our response: We thank the reviewer for the constructive suggestions and feedback, which helped to greatly improve our manuscript. We understand the reviewer's confusion regarding the orientation of chromosome 4A. Intuitively, one might argue that the orientation of chromosome 4A needs to be flipped based on the dot plot alignment. The rearrangements affecting chromosome 4A in polyploid wheat, however, have been extensively studied using cytogenetics and comparative genomics. The introduction of citation 22 provides a beautiful summary of the history that led to the chromosome 4A designation and orientation in polyploid wheat. We have tried to further clarify this in the text. We have moved the reference to Supplementary Fig. 3 to clarify that this figure shows a whole-genome alignment and does not aim to reconstruct the history of the chromosome 4A rearrangements in bread wheat. We also clarify that the chromosome rearrangements occurred in bread wheat and not in einkorn.
